# High false sign rates in transcriptome-wide association studies

**DOI:** 10.1038/s41588-026-02796-4

**Published:** 2026-10-22

**Authors:** Peter A. Gerlach, Nikhil Milind, Jeffrey P. Spence, Jonathan K. Pritchard

**Affiliations:** 1 Department of Genetics, Stanford University, Stanford, CA USA; 2 Institute for Human Genetics, University of California, San Francisco, CA USA; 3 Department of Epidemiology and Biostatistics, University of California, San Francisco, CA USA; 4 Department of Biology, Stanford University, Stanford, CA USA

**Keywords:** Genetic association study, Gene expression

## Abstract

Transcriptome-wide association studies (TWAS) are widely used to identify genes involved in complex traits and to infer the direction of gene effects on traits. However, despite their popularity, it remains unclear how accurately TWAS recover the true direction of a gene’s effect on a trait. Here we estimate the false sign rate of TWAS for plasma proteins, leveraging the expectation that increased gene expression should generally increase protein expression. We then extend this framework to complex traits, where loss-of-function burden tests provide the expected direction of effect. In both analyses, we observe high discordance with expectations, with TWAS showing a false sign rate of 23% for plasma proteins and 33% for complex traits. We propose that much of the sign discordance arises when expression quantitative trait loci in non-trait-relevant tissues tag haplotypes associated with traits in genome-wide association studies via distinct, tightly linked regulatory variants, yielding spurious TWAS associations with the correct genes but with unreliable direction of effect. These findings show that TWAS-based direction-of-effect estimates should be interpreted with caution and raise concerns about the reliability of TWAS more broadly.

## Abstract (web summary)

This study finds that the direction of effect captured by transcriptome-wide association studies is often reversed relative to the true biological mechanism, suggesting that caution is needed when interpreting these studies and using the results for downstream applications.

## Main

**Fig. 1:**
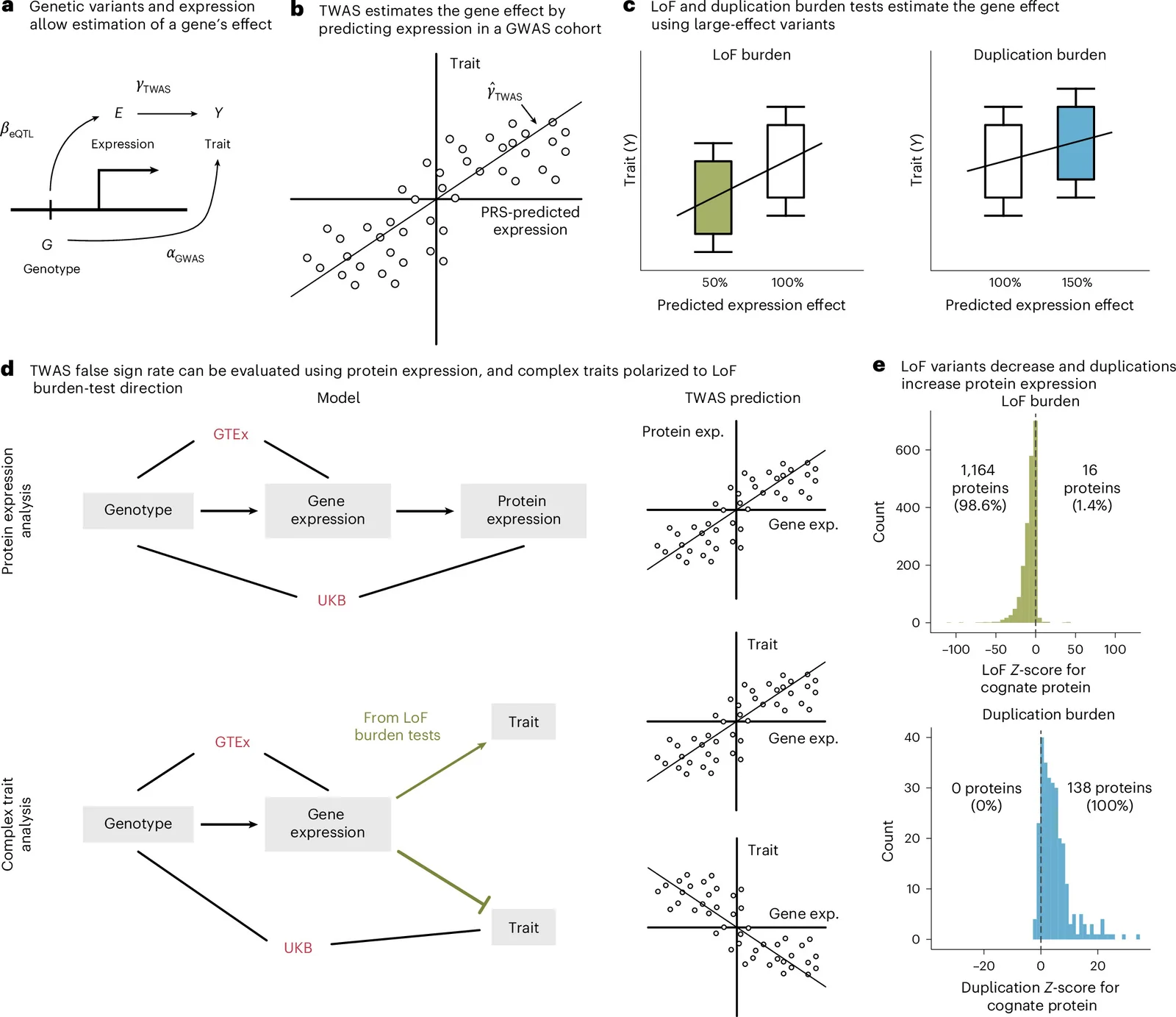
Workflow to evaluate the false sign rate of TWAS. **a**, TWAS estimates the sign and magnitude of effect of a gene on a trait. **b**, TWAS works by using SNPs in the *cis* region to build a polygenic risk score (PRS) for gene expression, and deploying it in a GWAS cohort to estimate the correlation between gene expression and trait value. **c**, Illustration of burden tests. Rare variants provide a gold-standard method to estimate the effect of a gene. LoF variants reduce gene expression, while duplications increase gene expression. We can measure their effect on a trait using burden tests. **d**, We used PRS models for gene expression trained in GTEx and deployed them in the UKB. We expect protein levels to have positive correlations with gene expression. For complex traits, we used the LoF burden test to determine which direction we expected from TWAS. We used these expectations to compute the FSR. **e**, For plasma proteins in the UKB, LoF variants decrease protein levels, and duplications increase protein levels. We report the number of LoF and duplication burden tests with significant effects on their cognate protein (two-sided test for *Z*-score, Bonferroni-corrected *P* value less than 0.05) on either side of the dashed line. This makes plasma protein levels an attractive phenotype to assess the FSR.

Genome-wide association studies (GWAS) have uncovered thousands of genetic associations with complex traits, but it remains challenging to connect these trait-associated variants with their underlying causal mechanisms. Transcriptome-wide association studies (TWAS), including FUSION^1^, PrediXcan^2^ and related methods such as SMR^3^, are widely used to identify genes that may be driving the GWAS signal at significant loci^4–17^ (Fig. 1a). By aggregating variant effects into gene-level signals, these methods provide more mechanistically interpretable information about the gene’s effect on a trait.

Conceptually, TWAS leverages genetic variants associated with gene expression known as expression quantitative trait loci (eQTLs) to infer each individual’s genetically predicted expression level for a gene, and then tests whether variation in the genetically predicted expression is associated with variation in the trait. In practice, this involves first building an expression prediction model using a dataset with both genotypes and expression data (for example, GTEx^18^), and then deploying this model in a dataset where genotypes and traits are available (for example, UK Biobank (UKB)^19^). By estimating the association between genetically predicted expression and the trait of interest, TWAS estimates both the magnitude and direction of effect of gene expression on the trait (Fig. 1b). Estimating the direction of gene effects distinguishes TWAS from other methods that aggregate GWAS signal at the gene level^20,21^ and is critical for downstream tasks, such as inferring coherent gene regulatory networks or interpreting the mechanisms of trait-associated genes for use in drug target discovery^22^.

Multiple independent evaluations of TWAS have been conducted, but all of them have focused on the false discovery rate for causal genes^23–27^. Here we instead focus on characterizing how well TWAS recovers the sign of the gene’s effect on a trait. As we will show, this allows us to detect many instances of spurious associations with the correct genes.

To evaluate the direction-of-effect estimates from TWAS, we assessed the false sign rate (FSR), which is the rate of disagreement between the TWAS-estimated effect direction and the biologically expected direction. Plasma proteomics provides a unique opportunity to evaluate TWAS direction-of-effect accuracy, because gene expression is expected to increase the abundance of its encoded plasma protein (Fig. 1d). Furthermore, we evaluated TWAS performance on predicting the direction of effect for various complex traits. Here we used the loss-of-function (LoF) effect direction as the ground truth to determine our expected gene effect direction for TWAS (Fig. 1d).

Our results show that even in the favorable setting of plasma proteins, TWAS frequently assigns the wrong direction, and that discordance is even more pronounced for complex traits benchmarked against LoF burden tests. We also show that colocalization-based filtering and focusing on trait-relevant tissues can partially reduce the FSR, but many loci still show confidently incorrect directions that are not well explained by simple genomic or gene-level features. Together, these findings demonstrate that current TWAS implementations can be highly unreliable for directional inference, and raise questions about whether TWAS and colocalization methods behave as they are commonly understood.

## Results

### Discordant TWAS direction-of-effect estimates for plasma proteins

To evaluate the FSR of TWAS direction-of-effect estimates, we used plasma protein expression as a positive-control GWAS phenotype, since higher genetically predicted expression of a gene is generally expected to increase the abundance of its encoded protein. While RNA and protein expression are imperfectly correlated, this reflects attenuation or buffering of RNA-level regulatory effects at the protein level, rather than reversal of their direction^28,29^. As a result, imperfect RNA–protein correlation would be expected to reduce power to detect TWAS associations between the expression of a gene and its protein abundance, but not reverse the estimated direction of effect. We therefore expect genetically predicted expression of the encoding gene to be positively associated with its protein expression (Fig. 1d).

To test this expectation, we used LoF and duplication burden tests, which are considered a ‘gold standard’ approach to determine the expected gene effect direction (Fig. 1c). Using protein expression measurements of 2,923 plasma proteins from the UK Biobank Pharma Proteomics Project (UKB-PPP)^30^, we tested whether LoF variants or duplications in the encoding gene were associated with altered protein expression of that same protein. Because LoF variants and duplications are expected to decrease and increase gene dosage, and consequently effective gene expression, they are expected to decrease and increase protein expression of the encoded protein, respectively. Indeed, we observed that 98.6% of proteins that are significant in LoF burden tests show decreased protein expression, and 100% of proteins that are significant in duplication burden tests show increased protein expression (Fig. 1e). Together, these results support the expectation that gene expression is generally positively associated with protein expression.

**Fig. 2:**
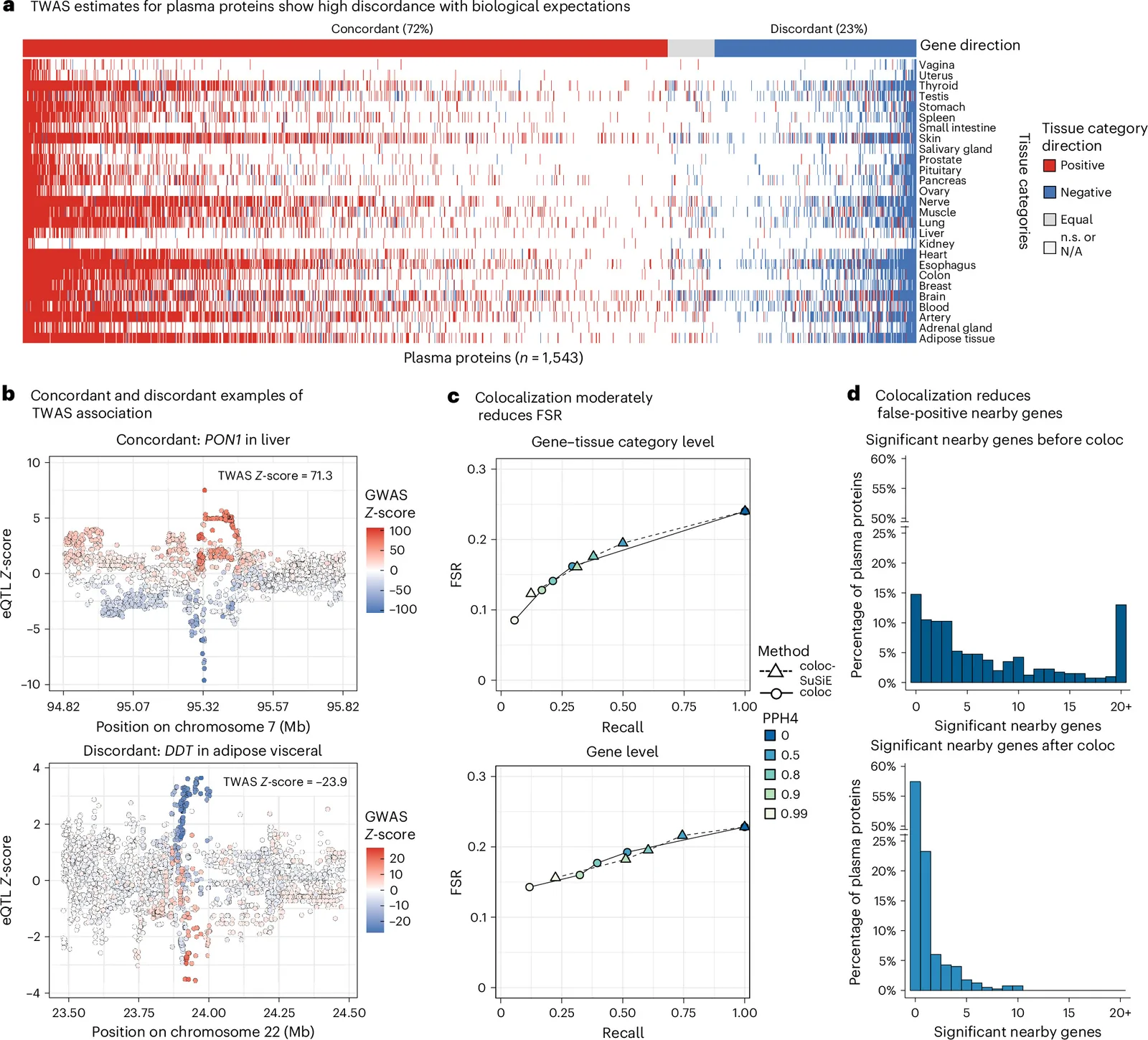
High discordance for TWAS direction-of-effect estimates for plasma proteins. **a**, Heatmap of TWAS direction-of-effect estimates for 1,543 plasma proteins (columns) across 27 tissue categories (rows). For each gene × tissue category, we assigned a direction by majority vote of significant (Bonferroni-corrected) *Z*-scores across tissues within that category. Tissue categories with a majority of positive *Z*-scores are colored red, majority negative blue, equal numbers of significant positive and negative gray, and categories with no significant *Z*-scores or no expression prediction model are white. Gene-level directions were assigned by majority vote across tissue categories, and genes are ordered by concordant (left) versus discordant (right) directions. **b**, Example loci illustrating concordant and discordant associations. Points show eQTL *Z*-scores (*y*-axis) versus genomic position, with variants colored by their GWAS *Z*-score. **c**, FSR versus recall for gene × tissue-category directions (top) and gene-level directions (bottom) after filtering by PPH4 using coloc (circles, solid line) or coloc-SuSiE (triangles, dashed line). **d**, Distribution of the number of TWAS-significant genes within ±2 Mb of each protein-encoding gene before (top) and after (bottom) filtering on PPH4 ≥ 0.5, among proteins with at least one colocalizing TWAS association.

To quantify the FSR of TWAS direction-of-effect estimates for plasma proteins, we applied the FUSION TWAS framework^1^ with expression prediction models trained in GTEx across 47 tissues. We applied these models to the same 2,923 plasma proteins from the UKB-PPP^30^, to test whether genetically predicted expression of each gene was associated with the protein expression of its encoded plasma protein. For the TWAS analysis, we excluded 16 proteins owing to either poor quality or multiple genes encoding the protein, resulting in 2,907 plasma proteins for further analysis (Methods). For each plasma protein, we estimated the direction of effect across 47 tissues based on a Bonferroni-corrected significance threshold, with each tissue assigned to one of 27 tissue categories (Supplementary Table 1). Of the 2,907 proteins, 383 (13%) had no FUSION association statistic produced in any tissue and 981 (34%) had no significant associations after Bonferroni correction, leaving 1,543 proteins for FSR estimation (Fig. 2a). First, we assigned a direction of effect for each gene–tissue category by taking a majority vote of significant associations across tissues within that category, and then assigned a gene-level direction by taking a majority vote across tissue categories. We then calculated the FSR across all genes that had at least one significant TWAS association in any tissue. Notably, the estimated FSR was 23% (95% confidence interval (CI), 20–25%), implying that, for roughly one in four genes, higher genetically predicted expression is associated with lower plasma protein levels, contrary to the expectation that increased gene expression typically increases protein abundance. Requiring stronger directional consistency across tissue categories modestly reduced but did not eliminate high FSR, indicating that the observed FSR is not driven solely by ambiguous majority-vote assignments (Supplementary Note 1).

To illustrate these patterns, we highlight examples of one concordant and one discordant gene–tissue association (Fig. 2b). In the concordant case of *PON1*, we observe that variants that increase expression of *PON1* in the liver also increase plasma protein levels of paraoxonase 1 (PON1) in the blood, as would be expected. By contrast, for the discordant case of *DDT*, we observe that variants that increase *DDT* expression are associated with lower plasma D-dopachrome tautomerase (DDT) levels.

Previous studies have proposed using colocalization-based filtering of TWAS associations to reduce false positives^31^, prioritizing genes whose eQTL and GWAS signals are consistent with a shared causal variant. To test whether adding a colocalization filter improved the FSR, we used coloc^32^ to calculate the posterior probability that the eQTL and GWAS associations are driven by a shared causal variant, known in coloc as the posterior probability of hypothesis 4 (PPH4). Furthermore, because the standard coloc model assumes a single causal variant per locus, we additionally used coloc-SuSiE, which couples coloc with sum of single effects (SuSiE) fine-mapping to allow multiple causal variants^33^. This allows colocalization to be tested separately for each fine-mapped signal^33^, increasing the chance of retaining TWAS associations when the eQTL colocalizes with one of several GWAS signals. We show the expected behavior of these filters using simulations of three distinct causal architectures: (i) coloc-based filtering retains associations when the GWAS and eQTL signals share a causal variant; (ii) coloc-based filtering removes associations when the signals are driven by distinct causal variants in moderate linkage disequilibrium (LD); and (iii) coloc-SuSiE-based filtering can improve recall when the eQTL colocalizes with one of multiple GWAS signals (Supplementary Note 2).

We calculated recall and FSR for different thresholds of PPH4 from coloc and coloc-SuSiE at the gene–tissue-category level and at the gene level (Fig. 2c). Recall was defined as the proportion of significant TWAS associations retained after coloc/coloc-SuSiE PPH4 filtering, relative to the unfiltered set of significant associations with available PPH4 estimates (excluding associations without PPH4). We found that filtering on PPH4 improved the FSR at both the gene–tissue-category and gene levels, with stricter thresholds yielding lower FSR (Fig. 2c). However, even at the strictest threshold (PPH4 = 0.99), the gene-level FSR remained above 0.1. Moreover, filtering on PPH4 led to a substantial drop in recall, and although coloc-SuSiE improved recall relative to coloc, it also yielded higher FSR at matched PPH4 thresholds. These estimates are largely consistent with a previous study integrating *cis*-Mendelian randomization methods with colocalization, which likewise reported a pronounced loss of recall under stringent colocalization thresholds^34^. Thus, colocalization-based filtering only partially resolves discordant TWAS directions for plasma proteins, and a substantial fraction of associations still appear to have incorrect signs even under stringent PPH4 thresholds.

To further characterize how different genetic architectures affected FSR and recall after colocalization filtering, we performed different simulations with varying minor allele frequency, effect sizes, eQTL sample sizes and LD between causal variants (Supplementary Notes 3 and 4). We found that high LD between the causal GWAS and causal eQTL was the strongest contributor to high FSR after colocalization filtering (Supplementary Fig. 3). Meanwhile, we found that stringent colocalization filtering also substantially reduced recall in scenarios with a true shared GWAS/eQTL causal variant (Supplementary Fig. 4).

We further assessed whether more recent TWAS approaches that explicitly model local genetic confounding could reduce FSR (Supplementary Note 5). To test this, we repeated the protein-expression analysis using cTWAS^35^, which jointly models molecular traits and nearby genetic variants within a locus. Applying the same tissue-category and gene-level majority-vote strategy as for FUSION, unfiltered cTWAS gene *Z*-scores showed similar discordance to FUSION: 24% (95% CI, 22–27%) versus 23% (95% CI, 20–25%) for cTWAS and FUSION, respectively (Supplementary Fig. 5a). While filtering associations by cTWAS posterior inclusion probability (PIP) modestly reduced FSR, it was accompanied by a substantial loss of recall (Supplementary Fig. 5b). Next, to assess whether multi-tissue modeling improved FSR, we applied multigroup cTWAS (M-cTWAS)^36^, an extension of cTWAS designed to jointly analyze multiple quantitative trait locus datasets. Because M-cTWAS produces tissue-specific gene PIPs rather than a single gene-level direction, we assigned each gene’s direction using the effect estimate in the tissue where that gene had the highest PIP. M-cTWAS showed similar FSR and recall to single-tissue cTWAS and FUSION (Supplementary Fig. 5c), indicating that integrating tissue-specific expression models did not substantially reduce the high FSR observed in the protein-expression analysis. These results suggest that the high FSR is not specific to FUSION and persists with newer TWAS approaches.

TWAS has been shown to often identify multiple nearby genes at a locus, many of which are likely non-causal ‘tagging’ genes whose predicted expression is correlated with that of the true causal gene owing to LD or co-regulation^23,37^. Because plasma levels of a given protein are primarily determined by its encoding gene, expression variation in nearby genes is generally not expected to directly influence that protein’s abundance. This setting therefore provides a convenient ground truth to quantify how many nearby TWAS-significant genes are likely false-positive tagging genes. To evaluate how colocalization filtering affects nearby signals, we compared the number of TWAS-significant genes within ±2 Mb of each protein-encoding gene before and after applying coloc filtering at PPH4 ≥ 0.5 (Fig. 2d). We observed that before colocalization filtering, 85.3% of protein-encoding genes showed at least one TWAS-significant nearby gene, suggesting that non-causal tagging signals are highly prevalent (Fig. 2d, top). After colocalization filtering we observed a substantial decrease in nearby significant genes (Fig. 2d. bottom), with 43.3% of protein-encoding genes showing at least one TWAS-significant nearby gene. These results indicate that colocalization filtering can markedly reduce nearby TWAS signals that are likely to be non-causal, although many nearby associations persist even after filtering.

### Complex traits show higher discordance of TWAS direction-of-effect estimates compared with plasma proteins

After assessing the FSR on plasma protein levels, we performed the same evaluation on complex traits by identifying 85 quantitative traits (Supplementary Table 2) for which GWAS summary statistics from the UKB were publicly available. For these traits, we curated ‘gold standard’ gene–trait pairs from exome-wide significant LoF burden tests, using the sign from the burden test to assign the true sign label to each gene–trait pair. We ascertained 730 gene–trait pairs using the LoF burden tests, which we evaluated using TWAS. While a few genes might have non-monotone gene dosage response curves (that is, both higher and lower expression of the gene have the same effect on the trait), we expect such curves to dilute the TWAS signal rather than confidently assign the incorrect direction^38^.

**Fig. 3:**
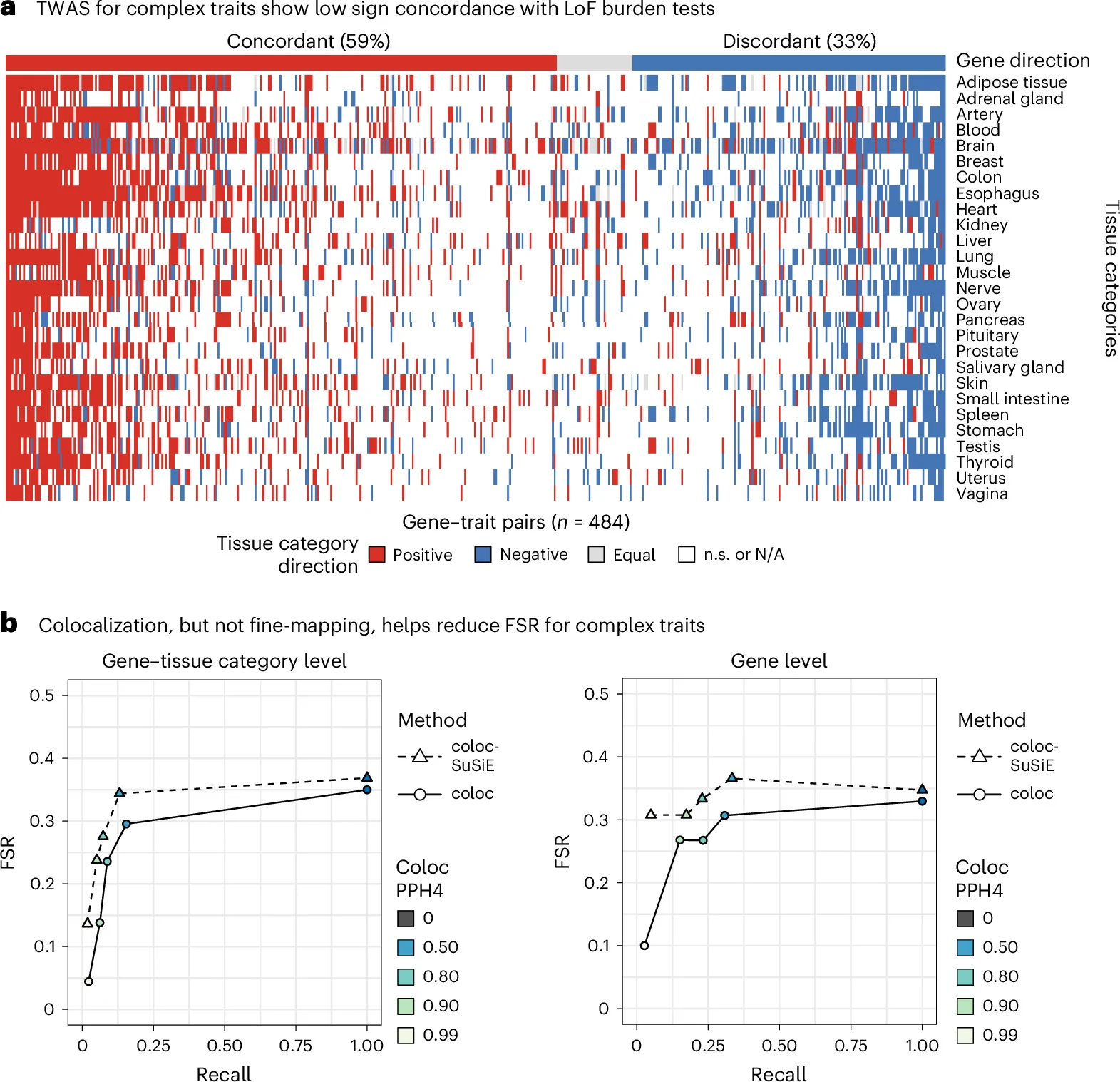
TWAS has a high FSR for complex traits. **a**, Heatmap of TWAS direction-of-effect estimates for 484 gene–trait pairs (columns) across 27 tissue categories (rows). For each gene–trait pair and tissue category, we assigned a direction by majority vote of significant (Bonferroni-corrected) *Z*-scores across tissues within that category. Tissue categories with a majority of positive *Z*-scores are colored red, majority negative blue, equal numbers of significant positive and negative gray, and categories with no significant *Z*-scores or no expression model are white. Gene-level directions were assigned by majority vote across tissue categories, and genes are ordered by concordant (left) versus discordant (right) directions. **b**, FSR versus recall for gene–trait pairs and tissue category directions (left) and gene-level directions (right) after filtering by PPH4 using coloc (circles, solid line) or coloc-SuSiE (triangles, dashed line).

For 484 of these gene–trait pairs, TWAS identified at least one significant association in a tissue (Fig. 3a). Since it is unclear which tissue is causal for any given complex trait or gene, we used the same majority-vote strategy as the plasma proteins to summarize the sign for each tissue category and gene. We found that 33% (95% CI, 29–38%) of gene–trait pairs had gene effects across tissue categories in the discordant direction. Strikingly, for 12% of the total gene–trait pairs (Fig. 3a, far right of the heatmap), TWAS was confident in the incorrect direction in greater than 90% of tissues with available expression-prediction models.

We again evaluated the use of colocalization and fine-mapping to improve the FSR (Fig. 3b). As with the proteins, using colocalization as a filtering strategy did reduce the FSR, although with a substantial loss of recall. Fine-mapping again resulted in a higher FSR, but with no concomitant increase in recall.

We built a simple linear model to explore why some gene–trait pairs had a substantially higher FSR than others (Fig. 3a) using gene density, local recombination rate, gene constraint measured by *s*_het_, mean *cis*-heritability of the gene and estimates of trait monotonicity. We accounted for the fact that some genes appear in multiple gene–trait pairs by using a random intercept for the trait and including the sample size for the trait’s GWAS as a fixed effect to account for power differences. Although the model fit the data well based on a likelihood ratio test against a null model with only a random intercept and the sample size (*P* = 3.6 × 10^−4^), implying that the predictors explained some of the variance in FSR, none of the individual predictors were convincingly associated with FSR (Supplementary Note 7). This suggests that there is no single gene-level factor among those that we tested that explains why some loci demonstrate striking discordance between TWAS and LoF burden test results.

### Misleading TWAS signals in non-trait-relevant tissues

**Fig. 4:**
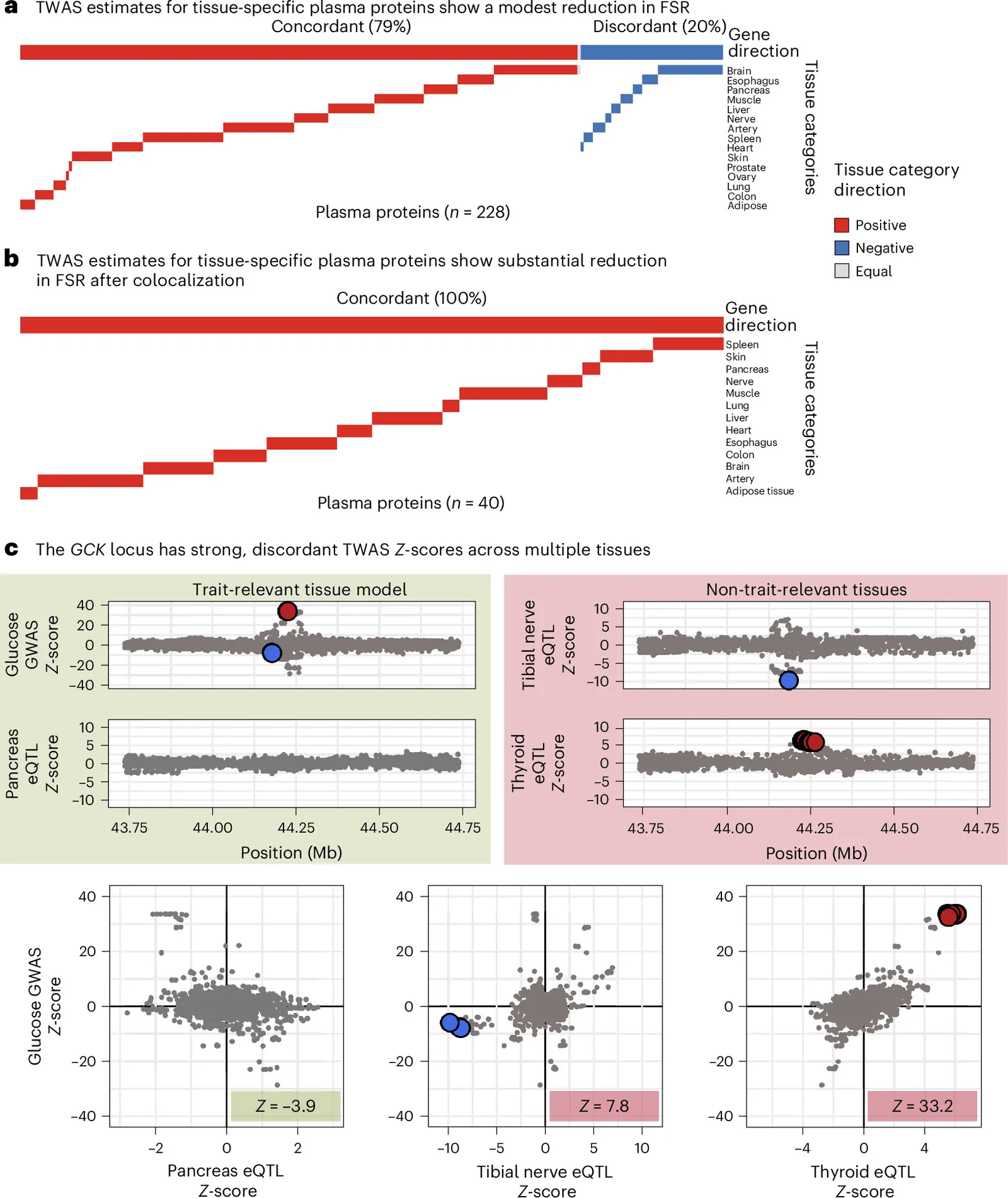
TWAS shows improved direction-of-effect estimates in trait-relevant tissues. **a**,**b**, Heatmap of TWAS direction-of-effect estimates for tissue-specific plasma proteins (columns), restricted to their assigned tissues (rows) based on annotations from Malmström et al.^39^. Tissue categories with a majority of positive *Z*-scores are colored red, majority negative blue and equal numbers of significant positive and negative gray. Genes are ordered by overall sign (positive, neutral and negative), and tissues (rows) are ordered by descending tissue FSR (highest FSR at the top). TWAS estimates are shown with no colocalization filtering (**a**, *n* = 228 genes) and after coloc filtering at PPH4 ≥ 0.5 (**b**, *n* = 40 genes). **c**, *GCK* is an example locus that shows strong discordance across multiple tissues. We expect a negative *Z*-score in pancreas but see little evidence of eQTL signal there. By contrast, we see colocalization with eQTL in tibial nerve and thyroid, which are strong but in the wrong direction. Blue and red points are 95% credible sets that colocalize between the eQTL and GWAS data.

**Extended Data Fig. 1:**
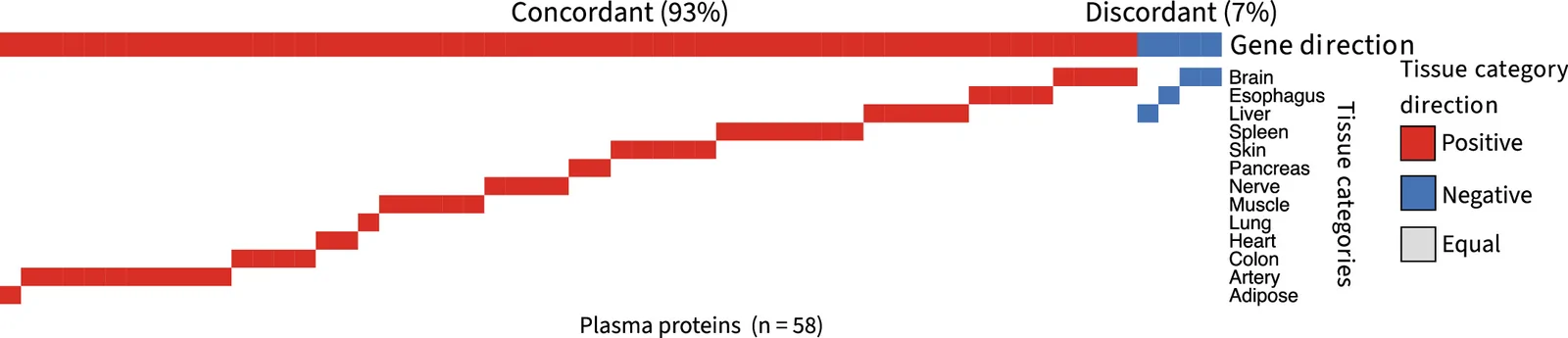
TWAS direction-of-effect estimates in trait-relevant tissues filtered by coloc-SuSiE. Heatmap similar to Fig. 4a and b of TWAS direction-of-effect estimates for tissue-specific plasma proteins (columns), restricted to their assigned tissues (rows). Tissue categories with a majority of positive Z-scores are colored red, majority negative blue, equal numbers of significant positive and negative grey. Genes are ordered by overall sign (positive, neutral, negative), and tissues (rows) are ordered by descending tissue FSR (highest FSR at the top). The heatmap shows TWAS estimates after coloc-SuSiE filtering at PPH4 ≥0.5 (n = 58 genes).

Previous work has shown that gene–trait associations are often tissue specific, motivating us to ask whether focusing on a trait-relevant tissue for each plasma protein could improve the direction-of-effect FSR compared with a majority vote across tissue categories^23,31^. Using tissue-of-origin labels from the Human Proteome Distribution Atlas^39^, we restricted to proteins specific to a single tissue, resulting in 228 proteins for which we defined gene direction using the TWAS *Z*-score in that tissue. Overall, restricting to the trait-relevant tissue only modestly reduced discordance, with 20% (95% CI, 15–26%) of proteins discordant using this approach (Fig. 4a), compared with 23% (95% CI, 20–25%) when using a majority-vote strategy across tissues (Fig. 2a). However, discordance was particularly high for brain-specific proteins, and excluding these associations reduced the FSR to 14% (95% CI, 9–20%). Notably, applying coloc filtering at PPH4 ≥ 0.5 further reduced discordance to 0% (95% CI, 0–9%), albeit for a limited number of genes (*n* = 40 genes) (Fig. 4b). Using coloc-SuSiE at the same threshold yielded an FSR of 7% (95% CI, 2–17%), but with higher recall (*n* = 58 genes) (Extended Data Fig. 1). Together, these results suggest that combining trait-relevant tissue information with colocalization-based filtering provides complementary evidence to improve the directional accuracy of TWAS effect estimates: tissue relevance supports the biological context of the association, while colocalization supports a shared genetic signal between the eQTL and GWAS. However, we recognize that identifying the relevant tissues for complex traits remains an open challenge^23,31,40^.

To illustrate these tissue-specific discrepancies in complex traits, we examined the association between *GCK* and blood glucose levels as an example. *GCK* encodes glucokinase (GCK), an enzyme that catalyzes the first step in glycolysis and acts as a glucose sensor in various tissues. LoF variants in *GCK* are associated with increased blood glucose levels^41–43^. The mechanism is thought to occur via pancreatic beta cells, where higher *GCK* increases insulin secretion and lowers blood glucose^44,45^. Therefore, for this gene, we expect TWAS to have a negative *Z*-score in pancreas, since increased *GCK* expression should be associated with reduced glucose levels. We found that even though there is a strong GWAS signal present at the *GCK* locus, there was no eQTL signal in the pancreas, resulting in no significant TWAS association in the tissue. However, visually, the direction of association in the pancreas appears to be correct (Fig. 4c), and using the top eQTL as the TWAS model results in a nominally significant association in the correct direction (*Z* = −3.9).

Surprisingly, *GCK* showed significant positive TWAS associations in 17 other tissue categories, even after coloc filtering at PPH4 ≥ 0.5. Paradoxically, the most prominent signals arose in tissues with no obvious mechanistic link to glucose homeostasis. The strongest *GCK* eQTL signal was in tibial nerve, where variants produced a significant positive TWAS association (*Z* = 7.8) (Fig. 4c). In addition, we observed a striking positive TWAS association between thyroid eQTL and glucose levels (*Z* = 33.2). We detected a single credible set in both tissues, which colocalized with two separate glucose GWAS credible sets (PPH4 = 0.51 and PPH4 = 0.99 in tibial nerve and thyroid, respectively). Taken together, the locus-level patterns in these tissues provide strong evidence that the same variants underlie both the eQTL and GWAS signals, but with an inferred direction of effect opposite to that expected from *GCK* biology (Fig. 4c). We explored some additional loci with similarly discordant associations across tissue groups, with varying colocalization evidence (Supplementary Note 6), many showing similarly strong evidence in the wrong direction.

Previous studies have suggested prioritizing tissues with the largest eQTL sample sizes, arguing that *cis*-regulated gene expression levels exhibit high genetic correlation across tissues and that well-powered proxy tissues can therefore substitute for the causal tissue when it is difficult to assay^37^. By contrast, our results show that relying on non-trait-relevant tissues in this way can yield highly significant but directionally misleading TWAS signals, with loci such as *GCK* displaying strong, colocalizing associations in the wrong direction. Given these results, we hypothesize that these discordant signals in non-trait-relevant tissues arise when GWAS and eQTL signals are driven by distinct variants in very high LD, which neither TWAS nor colocalization can reliably distinguish. In such settings, genes with little or no causal effect in a non-trait-relevant tissue can nonetheless inherit large TWAS *Z*-scores, simply because their expression tags the same underlying genetic variation that drives the true causal mechanism in an unobserved or underpowered trait-relevant tissue. We expect such patterns to arise if expression in the trait-relevant tissue is constrained relative to non-trait-relevant tissues, resulting in fewer discoverable eQTLs^46^. The eQTL in unconstrained tissues can be in high LD with the relevant eQTL but have a random sign relative to the trait.

To explore whether this mechanism could contribute to FSR, we simulated GWAS summary statistics for 1,000 genes under a model in which blood was the known causal tissue (Supplementary Fig. 9a; Supplementary Note 8). We then ran TWAS across all tissues and evaluated the FSR of non-blood associations as a function of their co-regulation with blood (Supplementary Fig. 9b). As expected, FSR was highest for strongly anti-correlated tissues and decreased with increasing co-regulation with blood, indicating that tissues with anti-correlated genetic effects on expression were most likely to produce false-sign associations. Notably, after restricting to gene–tissue associations with coloc PPH4 > 0.5, we observed enrichment not only for tissues positively correlated with blood, but also for strongly anti-correlated tissues. This pattern is consistent with a model in which non-causal tissue eQTLs can pass colocalization filtering when they tag the same GWAS-associated haplotype through very high LD. In such cases, the non-blood eQTL may have the opposite direction of effect from the causal blood eQTL, while still appearing to colocalize with the GWAS signal because of high LD.

## Discussion

Taken together, we show that TWAS direction-of-effect estimates show high discordance with biological expectations for both plasma proteins (23%) and complex traits (33%). While we found colocalization-based filtering to reduce the FSR, a substantial discordance still remained after filtering. We found that restricting gene-direction assignments to trait-relevant tissues and applying colocalization-based filtering markedly improved the FSR for plasma proteins, yielding no discordant cases among the remaining genes, but at the cost of retaining only a limited number of genes.

These results are broadly consistent with a failure mode in which the causal eQTL is in high LD with distinct eQTL variants in non-trait-relevant tissues, such that TWAS and colocalization correctly implicate the underlying haplotype but misattribute the tissue and direction of effect. We expect these patterns in part because regulatory variants that affect a gene’s expression only in non-trait-relevant tissues are likely to be under weaker purifying selection and can therefore attain larger effect sizes or higher frequencies than variants acting in trait-relevant tissues^46^. When such relaxed-constraint eQTLs are in high LD with the true causal variant, they can generate significant TWAS signals in non-relevant tissues. By contrast, focusing on trait-relevant tissues restricts attention to the eQTLs that plausibly mediate the biological effect on the trait, thereby testing the relevant eQTLs against the GWAS signal. Moreover, because non-trait-relevant tissues usually far outnumber trait-relevant tissues, these spurious but significant signals could easily end up dominating TWAS results for a gene tested across all tissues. The same issue may arise for cell-type-specific TWAS using single-cell data^47,48^—these models may improve direction-of-effect estimation when they capture the causal cell type, but testing many non-causal cell types can still produce discordant associations through linked eQTLs.

In addition to this LD-driven failure mode, other biological mechanisms could also contribute to the observed discordance. First, tissue-specific eQTL effects may play a role: while eQTL effect sizes are generally concordant across tissues^49^, some variants can exhibit opposite effects in different tissues^50^, potentially leading to mismatched gene-level directions when non-relevant tissues are included. Second, non-monotone gene–trait relationships^38^ may cause LoF and common-variant effects to differ in sign, though we expect this to be rare. Third, co-regulation of nearby genes can propagate the signal from a truly causal gene to its neighbors, yielding statistically significant but misleading TWAS associations^23,37^. Finally, a gene may have opposite effects on the trait in different tissues, so that LoF variants, which perturb all tissues at once, produce a net phenotypic effect whose sign can differ from tissue-specific eQTL or TWAS estimates.

Furthermore, technical factors may also partly explain the observed discordance. Because eQTL and GWAS are typically measured in different cohorts, TWAS often relies on an external LD reference panel. At loci where abundant GWAS signal is present, the TWAS model may be confident that an association is present (resulting in a large absolute *Z*-score), but subtle mismatches in LD patterns may cause sign flipping. In addition, most TWAS frameworks treat predicted expression as fixed, ignoring uncertainty in the expression imputation model, which can induce spurious associations with essentially random sign at strongly associated loci^51^.

We note that the observed discordance should not be interpreted as evidence that TWAS itself introduces erroneous signs, but rather that the discordance can arise from the underlying eQTL data used by TWAS and other eQTL-based methods. Nevertheless, it remains a practical limitation when TWAS signs are interpreted as biological direction-of-effect estimates. We recommend that current and future TWAS methods should be evaluated for their FSR in addition to their false discovery rate. Our analyses show that protein quantitative trait locus (pQTL) datasets are an attractive source of phenotypes to test the FSR, since we rarely expect to see a negative relationship between the genetic component of gene expression and protein levels.

While LoF burden tests often act as a ‘gold standard’ for gene sign, ascertainment of burden tests prioritizes genes by their specificity to the trait, suggesting that the genes we considered may not be representative of all genes^52^. Common-variant signals from GWAS can be more context specific, which would allow us to infer the sign of genes that may be too constrained to be discovered by burden tests. TWAS methods are invaluable for summarizing the evidence for the sign of a gene’s effect from common variants. Continued development and refinement of these methods will be crucial for understanding the causal, mechanistic role of genes in various traits and diseases.

## Methods

### Burden test summary statistics

We retrieved burden test summary statistics from our prior analysis in the UKB^38^. For the protein burden tests, we retrieved the test results for only the cognate gene.

To identify ‘top hits’ from the LoF burden tests for complex traits, we used a Bonferroni threshold based on the number of genes and number of traits. At the 5% significance level, the corrected *P*-value threshold was 3.32 × 10^−8^. At this threshold, we identified 730 significant gene–trait pairs in 73 of the 85 chosen complex traits.

### TWAS

For protein expression analyses, we used pQTL summary statistics for participants of European ancestry (*n* = 34,557) from the UKB-PPP^30^. Of the 2,923 plasma proteins, 1 protein (GLIPR1) was excluded owing to data failing quality control^30^, and 15 plasma proteins were excluded owing to multiple genes encoding the proteins (CKMT1A_CKMT1B, DEFA1_DEFA1B, DEFB4A_DEFB4B, EBI3_IL27, FUT3_FUT5, IL12A_IL12B, LGALS7_LGALS7B, MICB_MICA, AMY1A_AMY1B_AMY1C, BOLA2_BOLA2B, CGB3_CGB5_CGB8, CTAG1A_CTAG1B, DEFB103A_DEFB103B, DEFB104A_DEFB104B, SPACA5_SPACA5B), resulting in 2,907 plasma proteins used for TWAS associations.

For complex traits, we used summary statistics from the Neale Lab for 85 traits measured in the UKB (Supplementary Table 2).

We used FUSION to perform TWAS^1^. We performed TWAS between all genes and traits across all GTEx tissues for which expression models were available. We used the GTEx expression models and 1000 Genomes Project LD reference panel provided with the FUSION software.

### cTWAS and M-cTWAS

For the protein expression analysis, we used the same UKB-PPP pQTL summary statistics^30^ as in the FUSION analysis, together with GTEx v8 MASHR PredictDB expression models^2,31,53^ across tissues. The pQTL summary statistics were harmonized and imputed with the summary-gwas-imputation pipeline (https://github.com/hakyimlab/summary-gwas-imputation) to align variants, alleles and *Z*-scores with the PredictDB expression models. We used European ancestry hg38 LD block annotation and UKB LD matrices provided with cTWAS^35^.

We ran cTWAS^35^ for all proteins across all available GTEx tissues. For each protein, we restricted the analysis to a local region defined as the LD block containing the corresponding protein-coding gene and two LD blocks on either side for computational feasibility. Tissues with fewer than 30 usable molecular traits after preprocessing were excluded. For multigroup M-cTWAS^36^, we jointly analyzed all available tissue-specific PredictDB expression models for each protein. As in the single-tissue analysis, tissue groups with fewer than 30 usable molecular traits after preprocessing were excluded. From both cTWAS and M-cTWAS, we extracted gene *Z*-scores and PIPs for downstream FSR analyses.

### Annotating gene direction

To determine the overall direction of gene effects, we performed a hierarchical majority vote. First, for each tissue category, we determined the direction by majority vote across its tissues. Next, we performed a majority vote across all tissue categories to assign the final direction for each gene. The grouping of GTEx tissues into categories is provided in Supplementary Table 1. For each FSR estimate, we calculated exact binomial CIs using the binom.test function from the stats package in R version 4.5.1 (ref. ^54^), with discordant signs as successes and the total number of evaluated associations as trials.

### Colocalization and fine-mapping

We used the susieR package^55,56^ to perform fine-mapping of the eQTL and GWAS summary statistics. We performed fine-mapping on 1 Mb regions centered at the transcription start site for all genes in GENCODE v39 (ref. ^57^). We used genotypes from individuals assigned to the ‘EUR’ superpopulation in the 1000 Genomes Project^58^ to construct the LD reference panel. We required that at least ten variants needed to be present in both the summary statistics and the LD panel to proceed with fine-mapping. We used the susie_rss function to fit the SuSiE regression model to the summary statistics and LD panel.

We tested for colocalization using the coloc package^32,33^. To perform colocalization only, we used the approximate Bayes factor approach implemented in the original package^32^, which assumes one causal variant at each locus. To perform colocalization with SuSiE, we tested all pairwise combinations of the independent signals detected between eQTL and GWAS summary statistics using the coloc.bf_bf function. We excluded any loci that did not have at least 100 overlapping variants between the eQTL and GWAS summary statistics from the colocalization analysis.

### Simulation of GWAS summary statistics

All GWAS summary statistics were simulated using the sim_mv_determined function from the GWASBrewer package^59,60^ in R version 4.5.1 (ref. ^54^). To approximate the correlation structure observed in UKB-PPP, we used LD matrices and allele frequencies from the UKB computed in ref. ^35^. For each simulated gene, we restricted the simulation to a local genomic window centered on the causal eQTL, using only variants and LD information from UKB LD blocks overlapping that region. The sample size was fixed at *N* = 30,000, chosen to approximate the sample size of the European-ancestry pQTL summary statistics. One or two causal variants (depending on the scenario) were specified, with their per-allele effect parameterized to achieve pre-specified *Z*-scores, while all other variants were assigned zero direct effect size.

For larger-scale simulations (Supplementary Notes 3, 4 and 8), for each simulated gene, the causal eQTL was defined as the top1 eQTL variant from the GTEx expression model. To ensure consistency between the simulated causal architecture and the TWAS model being evaluated, we forced FUSION to use the top1 prediction model in all simulation analyses. Furthermore, we restricted to genes whose expression models passed the FUSION *cis*-heritability filter (passes_hsq = TRUE), ensuring that each simulated gene had evidence of genetically regulated expression.

### Cross-tissue correlation of predicted expression

To quantify similarity between tissue-specific expression prediction models for the same gene, we calculated the correlation between genetically predicted expression across tissues using the SNP weights from the FUSION models and the corresponding LD matrix. For tissues *i* and *j*, with weight vectors *w*_*i*_ and *w*_*j*_, respectively, the correlation was calculated as

$$\mathrm{cor}\left({{\hat{E}}_{i}},{{\hat{E}}_{j}}\right)=\frac{{w}_{i}^{{\top }}R{w}_{j}}{\sqrt{\left({w}_{i}^{{\top }}R{w}_{i}\right)\left({w}_{j}^{{\top }}R{w}_{j}\right)}}$$

where *R* denotes the SNP LD correlation matrix and ${\hat{E}_{\mathrm{i}}}$ and ${\hat{E}_{j}}$ are the genetically predicted expression for the gene in tissues *i* and *j*, respectively^37^.

### Tissue specificity

To assess tissue specificity of TWAS signals (Fig. 4a,b), we filtered to genes annotated as tissue specific to a single tissue and with a global label score ≥1 in ref. ^39^.

### Reporting summary

Further information on research design is available in the Nature Portfolio Reporting Summary linked to this article.

## Online content

Any methods, additional references, Nature Portfolio reporting summaries, source data, extended data, supplementary information, acknowledgements, peer review information; details of author contributions and competing interests; and statements of data and code availability are available at https://doi.org/10.1038/s41588-026-02796-4.

## Supplementary information

Supplementary Information Supplementary Notes 1–8, Figs. 1–9 and Tables 1 and 2.

Reporting Summary

Peer Review File

## Data Availability

The data generated are available via Zenodo at https://doi.org/10.5281/zenodo.17990927 (ref. ^61^). The data processed by this study can be accessed via the following URLs: Protein QTL Summary Statistics (https://www.synapse.org/Synapse:syn51364943/files/); Neale Lab’s GWAS Summary Statistics (http://www.nealelab.is/uk-biobank); Tissue Weights from TWAS Website derived from GTEx v8 (http://gusevlab.org/projects/fusion/); LD from 1000 Genomes Project for FUSION (https://alkesgroup.broadinstitute.org/FUSION/); 1000 Genomes Project VCFs for colocalization and fine-mapping LD Panel (https://ftp.1000genomes.ebi.ac.uk/vol1/ftp/release/20130502/, http://ftp.1000genomes.ebi.ac.uk/vol1/ftp/data_collections/1000_genomes_project/release/20190312_biallelic_SNV_and_INDEL/); GTEx eQTL Summary Statistics for Coloc and SuSiE from the eQTL Catalogue (https://www.ebi.ac.uk/eqtl/); LoF Burden Test Summary Statistics (https://zenodo.org/records/16800547); LD and AF summary from UKB for GWAS simulations (https://uchicago.app.box.com/s/jqocacd2fulskmhoqnasrknbt59x3xkn/folder/234629250877).

## Code availability

The code is available via Zenodo at https://doi.org/10.5281/zenodo.17990927 (ref. ^61^).
