# Supplementary material for: High false sign rates in transcriptome-wide association studies
Source: Nat Genet. 2026 Oct 22;58(11):3004–12. doi: 10.1038/s41588-026-02796-4 (PMC13754616; doi:10.1038/s41588-026-02796-4)
Supplement: Supplementary file 1 [file 41588_2026_2796_MOESM1_ESM.pdf]

nature genetics

Supplementary information https://doi.org/10.1038/s41588-026-02796-4

High false sign rates in transcriptome-wide association studies

In the format provided by the authors and unedited

Contents

Supplementary Note 1: Increasing the majority-vote threshold modestly decreases FSR **2**

Supplementary Note 2: coloc filtering reduces false positives and coloc-SuSiE retains recall in simulations **4**

Supplementary Note 3: Effects of genetic architectures on FSR **6**

Supplementary Note 4: Effects of genetic architectures on recall **8**

Supplementary Note 5: cTWAS shows similarly high levels of FSR **11**

Supplementary Note 6: Discordant biological examples for complex traits **13**

GCK and Blood Glucose . . . 13

CXCL2 and Neutrophil Percentage . . . 13

GMPR and Mean Corpuscular Hemoglobin . . . 15

ADAMTS17 and Height . . . 16

Supplementary Note 7: Linear model of FSR across gene-trait pairs **17**

Supplementary Note 8: Strong anti-correlation between tissues may create false-positive evidence of colocalization **19**

List of Figures

1 Stricter majority-vote thresholds modestly decrease FSR while highlighting variability in direction across tissue categories. . . . 3

2 Simulations of TWAS and colocalization under different causal architectures. . . . 5

3 The effect of genetic architecture on FSR. . . . 7

4 The effect of genetic architecture on recall. . . . 10

5 cTWAS and M-cTWAS show similarly high levels of false sign rates. . . . 12

6 TWAS associations at the CXCL2 Locus. . . . 14

7 TWAS associations at the GMPR Locus. . . . 15

8 TWAS associations at the ADAMTS17 Locus. . . . 16

9 Anti-correlated tagging tissues can produce false-positive evidence of colocalization. 20

List of Tables

1 Tissues from GTEx grouped into tissue categories. . . . 21

2 Complex traits used for analysis. . . . 22

Supplementary Note 1: Increasing the majority-vote threshold modestly decreases FSR

In the main protein expression analysis, we assigned each gene an overall direction using a simple majority vote across tissue categories. To assess whether requiring stronger directional consistency would improve FSR, we repeated the analysis using more stringent majority thresholds for assigning direction to genes. Specifically, we compared the default 50% threshold with thresholds of 60%, 70%, 80%, 90%, and 100%, and evaluated the FSR across these thresholds (Figure S1A). In addition, to quantify gene-level variability in direction across tissue categories, we plotted the proportions of tissue categories showing positive, negative, or equal direction for each gene (Figure S1A, bottom). This analysis showed that 36.4% of genes had both positive and negative tissue-category directions, indicating substantial variability in direction across tissue categories within genes. Among these genes, 56.8% were assigned an overall positive direction, 29.0% an overall negative direction, and 14.2% were assigned as equal under the 50% majority-vote threshold.

Next, we combined the gene-direction assignments defined by the different majority thresholds with colocalization filtering to evaluate FSR and recall (Figure S1B). We first calculated FSR and recall using all retained genes in the denominator, including genes classified as equal (Figure S1B, left). Because a substantial fraction of genes become equal at higher majority thresholds, this representation can be somewhat misleading, as it makes stricter majority thresholds appear to achieve low FSR while retaining much higher recall. To address this, we also calculated FSR using only positively and negatively classified genes in the denominator (Figure S1B, right). This representation shows that increasing the majority threshold modestly decreases FSR while having only a small effect on recall. Together, this highlights that a stricter majority-vote threshold can modestly improve sign calibration. However, it does not eliminate the broader problem that TWAS direction-of-effect estimates may vary across tissue categories when non-trait-relevant tissue signals tag the underlying GWAS signal through distinct regulatory variants in very high linkage disequilibrium, making direction inferred from non-trait-relevant tissues potentially misleading.

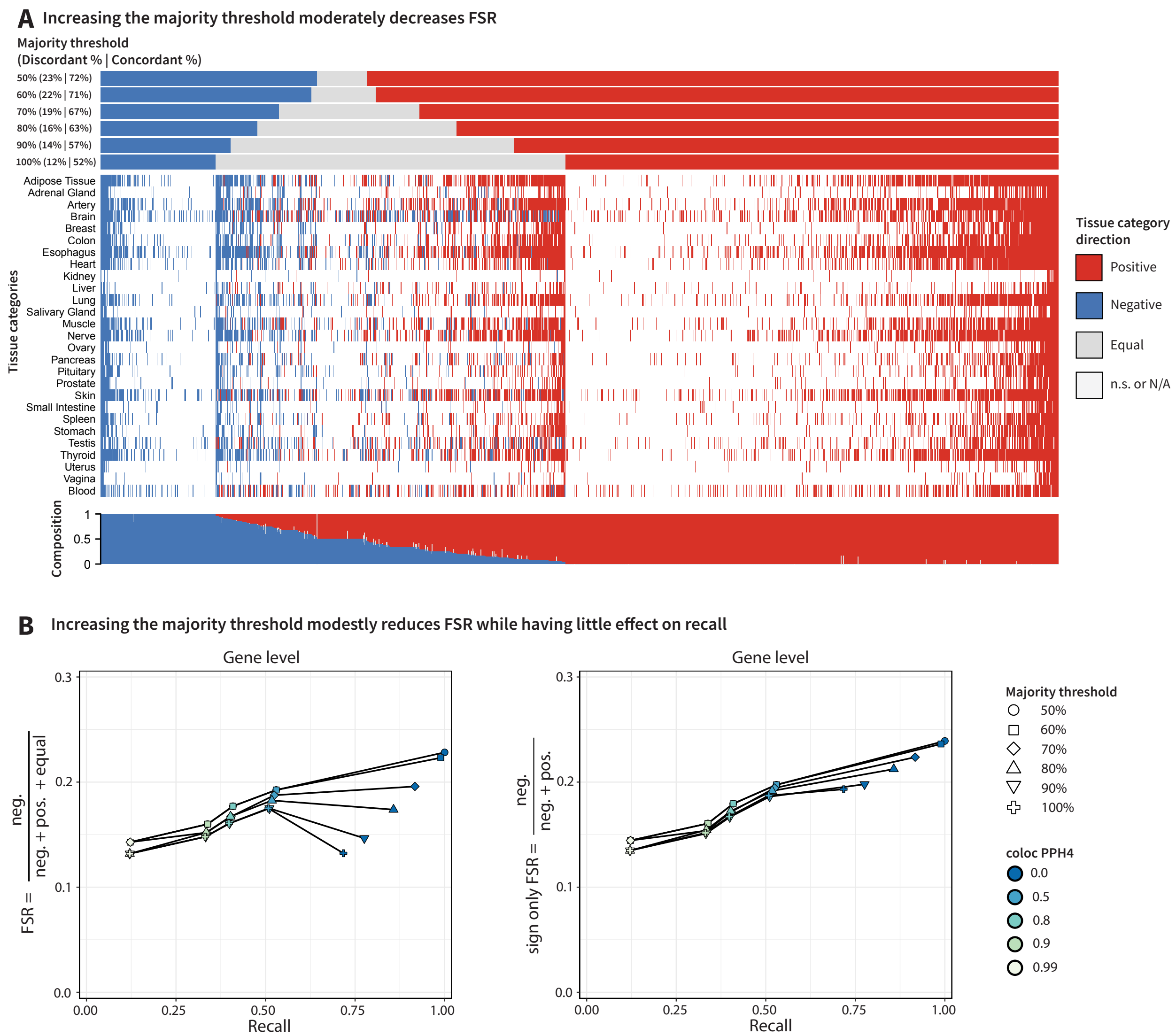

Supplementary Figure 1. Stricter majority-vote thresholds modestly decrease FSR while highlighting variability in direction across tissue categories. A. Heatmap of TWAS direction-of-effect estimates for 1,543 plasma proteins (columns) across 27 tissue categories (rows), shown together with gene-level direction assignments under majority-vote thresholds of 50%, 60%, 70%, 80%, 90%, and 100%. Gene-level directions were assigned across tissue categories using the indicated majority-vote thresholds. The bottom composition bar shows, for each gene, the proportions of tissue categories with positive, negative, or equal directional evidence. **B.** FSR versus recall for gene-level directions after filtering by coloc PPH4 and assigning gene direction using majority-vote thresholds of 50%, 60%, 70%, 80%, 90%, or 100%. In the left panel, FSR was calculated as the proportion of negative genes among all retained genes, including neutral genes. In the right panel, FSR was calculated as the proportion of negative genes among retained signed genes only.

Supplementary Note 2: coloc filtering reduces false positives and coloc-SuSiE retains recall in simulations

To assess how coloc and coloc-SuSiE affect false positives and recall, we simulated TWAS data under different scenarios. To most realistically simulate the plasma protein TWAS associations from Figure 2, we simulated GWAS summary statistics with the GWASBrewer package [1, 2] using LD matrices and allele frequencies from the UK Biobank computed in ref [3] (Figure S2A-C). Because LD matrices and allele frequencies from GTEx were unavailable, we used liver eQTL summary statistics at the MBL2 locus, which exhibited a clear signal, and assumed that the top eQTL variant was causal (Figure S2D-F). Simulations were restricted to a 1 Mb window centered on the MBL2 locus, using variants, allele frequencies, and LD from the corresponding UK Biobank LD block. We then calculated TWAS Z-scores with FUSION using the provided 1000 Genomes LD reference, as done in the plasma protein analyses. Finally, we computed PPH4 using both coloc and coloc-SuSiE, reporting the maximum coloc-SuSiE PPH4 across all SuSiE credible-set pairs (Figure S2G-I).

In the first scenario, we simulated a "true positive" case in which the causal variant was the same in both eQTL and GWAS summary statistics (Figure S2A, S2D, and S2G). As expected, we observe a highly significant TWAS Z-score and a high colocalization probability from both coloc and coloc-SuSiE.

In the second scenario, we simulated the GWAS causal variant to differ from the causal eQTL variant but to be in modest LD with it ($R^2 = 0.35$) (Figure S2B, S2E, and S2H). Although the TWAS Z-score remained significant, both coloc and coloc-SuSiE provided no evidence of colocalization. This indicates that coloc-based methods can correctly filter out “false positive” TWAS signals when the causal variants are in moderate LD.

In the final scenario, we simulated two distinct GWAS causal variants in which the smaller-effect variant was the same as the top eQTL variant and the larger-effect GWAS variant being effectively unlinked with the eQTL causal variant ($R^2 = 0.002$) (Figure S2C, S2F, and S2I). In this scenario we still observe a strong significant TWAS signal. However, because coloc assumes a single causal variant per trait, it attributes the GWAS signal to the larger-effect variant and consequently detects no colocalization with the eQTL causal variant (Figure S2I). Meanwhile, coloc-SuSiE provides strong evidence of colocalization, as it can resolve multiple distinct association signals at the locus and assess colocalization for each pair of signals between traits. Thus, coloc-SuSiE should be able to retain TWAS associations in which smaller effect causal variants colocalize to improve recall.

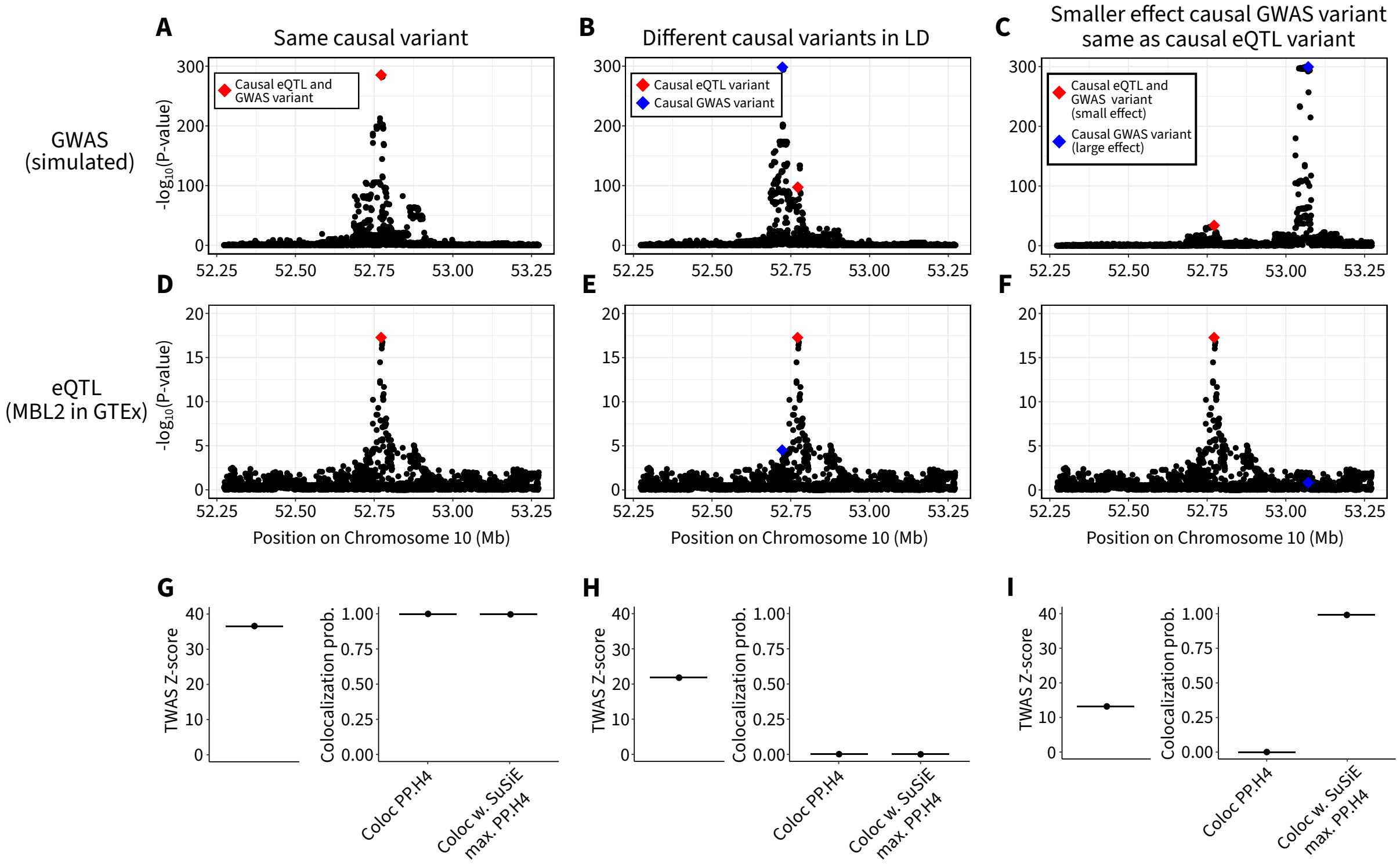

Supplementary Figure 2. Simulations of TWAS and colocalization under different causal architectures. A-C. Simulated GWAS association statistics at the *MBL2* locus for three scenarios: (A) a shared causal variant between GWAS and eQTL, (B) distinct GWAS and eQTL causal variants in LD ($R^2 = 0.35$), and (C) two GWAS causal variants where the smaller-effect variant is the same variant as the eQTL causal variant and the larger-effect variant is unlinked to the eQTL causal variant ($R^2 = 0.002$). **D–F,** eQTL summary statistics for the *MBL2* locus in liver tissue. **G–I,** TWAS Z-scores and colocalization posterior probability PPH4 from coloc and coloc-SuSiE for each scenario; for coloc-SuSiE we show the maximum PPH4 across SuSiE credible sets. Lines in **G–I** represents the mean value across 50 simulations.

Supplementary Note 3: Effects of genetic architectures on FSR

To evaluate how colocalization filtering is expected to reduce FSR under different genetic architectures, we simulated a non-causal tissue architecture in which the causal GWAS variant was distinct from the causal eQTL (Figure S3A) using the GWASBrewer package [1, 2]. For each gene, simulations were restricted to an 8 Mb window centered on the causal eQTL, and the causal GWAS variant was sampled to vary in LD with the causal eQTL, minor allele frequency (MAF), and effect size. In total, we simulated 1,500 genes across three tissues with different sample size, with 500 genes each in Whole Blood (n = 558), Colon Transverse (n = 294), and Spleen (n = 179), and then applied the same TWAS and colocalization filtering procedure used in the main protein expression analysis. Because the simulated GWAS causal variant was always distinct from the causal eQTL, all significant TWAS associations were treated as false positives, and assuming false-positive signs are random, we calculated FSR as FDR/2 (Figure S3B-F).

Across the genetic architecture variables considered, LD between the causal GWAS variant and the causal eQTL was the strongest determinant of FSR after colocalization filtering (Figure S3B). In particular, when the causal GWAS variant was in high LD with the causal eQTL ($R^2 = 0.8 - 1.0$; rightmost bin in Figure S3B), elevated FSR persisted even after colocalization filtering. Increased GWAS signal strength was also associated with higher FSR, although this effect was mitigated by colocalization filtering (Figure S3F). In contrast, we observed no clear relationship between FSR, and the other genetic architecture variables considered, including the MAF of the causal GWAS variant, eQTL sample size, and top eQTL Z-score (Figure S3C-E). However, because genes included in these simulations were required to pass the FUSION cis-heritability filter, this analysis is restricted to genes with detectable genetically regulated expression, which may limit our ability to observe the effects of different eQTL sample sizes.

Together, these findings support our hypothesis that FSR is driven by non-causal tissues whose eQTLs tag the true causal GWAS signal through very high LD, creating association patterns which neither TWAS nor colocalization can reliably distinguish.

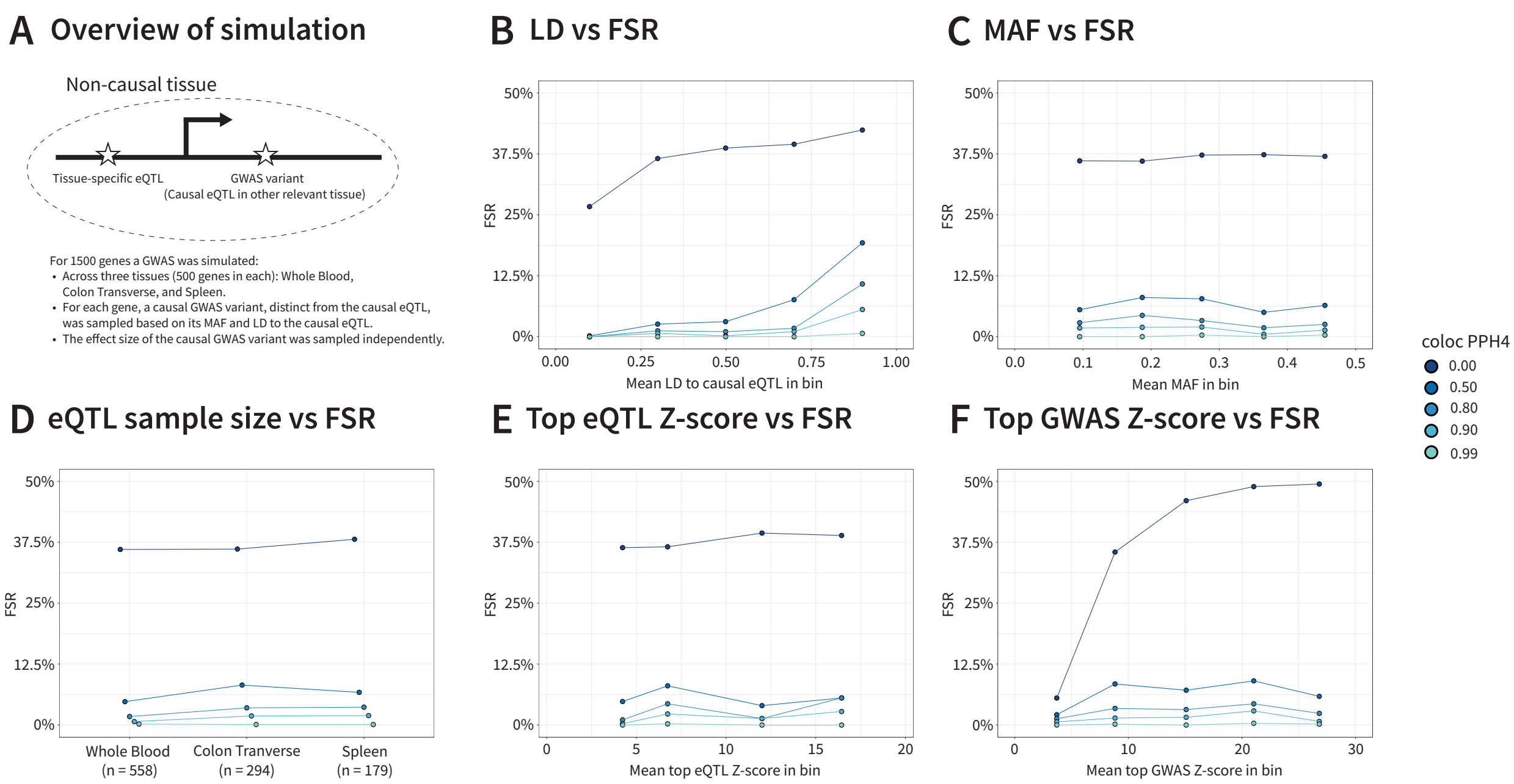

Supplementary Figure 3. The effect of genetic architecture on FSR. A. Simulation framework. For each gene, a causal GWAS variant was sampled to be distinct from the causal eQTL, with varying LD to the causal eQTL, MAF, and effect size. Simulations were performed across Whole Blood, Colon Transverse, and Spleen. **B-F.** FSR was estimated after binning gene-tissue pairs by mean LD to the causal eQTL (**B**), MAF (**C**), eQTL sample size (**D**), top eQTL Z-score (**E**), and top GWAS Z-score (**F**). Colors indicate coloc PPH4 filtering thresholds.

Supplementary Note 4: Effects of genetic architectures on recall

To evaluate how colocalization filtering affects recall under different genetic architectures when a true colocalizing signal is present alongside an additional GWAS signal, we simulated a two-causal-variant GWAS architecture using the GWASBrewer package [1, 2]. Specifically, one GWAS causal variant was fixed to be the causal eQTL, (assumed to be the top eQTL), while a second, non-shared GWAS causal variant was sampled to vary in LD with the causal eQTL and vary in MAF (Figure S4A). For each gene, simulations were restricted to an 8 Mb window centered on the causal eQTL. We considered three effect-size scenarios for the simulated causal GWAS variants: equal strength of the shared and non-shared GWAS variants (target GWAS Z-score = 10 for both), a scenario in which the shared causal eQTL/GWAS variant had the stronger effect (target GWAS Z-score = 15 for the shared eQTL/GWAS variant and 5 for the non-shared variant) , and a scenario in which the non-shared GWAS variant had the stronger effect (target GWAS Z-score = 5 for the shared eQTL/GWAS variant and 15 for the non-shared variant). For each of these three scenarios, we simulated 1,500 genes across three tissues, with 500 genes each in Whole Blood, Colon Transverse, and Spleen, and evaluated recall after applying the same TWAS and colocalization filtering procedure used in the main protein expression analysis. Together, this setup allowed us to test how different variables of genetic architectures affect recall when the eQTL-colocalized GWAS signal is accompanied by an additional GWAS signal.

Overall, we observed that stringent colocalization filtering substantially reduced recall. Specifically, filtering at coloc PPH4 ≥ 0.99 resulted in recall below 10% in most scenarios, except when the eQTL signal was strong, where recall improved (Figure S4B-E). This highlights that even when one GWAS causal variant is simulated to be shared with the causal eQTL, colocalization filtering can remove true-positive TWAS associations under stringent PPH4 thresholds, particularly when the shared eQTL signal is modest or accompanied by an additional GWAS signal. One limitation is that the simulated causal eQTL was defined from the top1 FUSION model, whereas colocalization used separate unpruned eQTL summary statistics as in the main protein expression analysis. Thus, the top FUSION eQTL may not always correspond exactly to the variant most strongly supported in the colocalization analysis, which may contribute to reduced recall.

The relationship between LD and recall depended strongly on which GWAS signal was stronger (Figure S4B). In the first scenario, where the shared and non-shared GWAS variants had equal strength, recall showed a U-shaped pattern, with higher recall at low and high LD and lower recall at intermediate LD. This likely reflects two different ways a TWAS association can be retained: at low LD, the shared eQTL/GWAS variant contributes a distinct signal that matches the expression model, whereas at high LD, the shared and non-shared GWAS variants become difficult to distinguish and can resemble a single colocalized signal. At intermediate LD, the combined GWAS association pattern may be less well matched to the eQTL weights, reducing recall. In the second scenario, where the non-shared GWAS variant was stronger, recall increased with LD. In this scenario, the dominant GWAS association is driven by the non-shared variant, so high evi-

dence of colocalization can arise when this stronger GWAS signal tags the eQTL through LD (like the scenario in Supplementary Note 3 with a single causal GWAS variant distinct from the causal eQTL in Figure S3B), even though this GWAS variant and the causal eQTL variant are distinct. Thus, increased recall in this setting may partly reflect colocalization between the stronger non-shared GWAS signal and the eQTL, rather than clean recovery of the weaker shared eQTL/GWAS signal. In contrast, in the last scenario when the shared eQTL/GWAS variant was stronger, recall was higher and independent of LD, consistent with the primary GWAS signal corresponding to the causal eQTL.

We observed no clear relationship between recall and either MAF or eQTL sample size (Figure S4C-D), although this should be interpreted considering our restriction to genes that passed the FUSION cis-heritability filter, which may limit variation in eQTL detectability across tissues. In contrast, the relationship between recall and top eQTL Z-score depended on the effect-size scenario (Figure S4E). When the shared eQTL/GWAS signal was equal in strength to, or stronger than, the non-shared GWAS signal, higher eQTL Z-scores were associated with higher recall, consistent with stronger eQTL evidence improving recovery of the true colocalized signal. However, when the non-shared GWAS variant was stronger, higher eQTL Z-scores were associated with lower recall after colocalization filtering, likely because a stronger eQTL signal makes it easier to distinguish the eQTL from the dominant non-shared GWAS signal and therefore reduces apparent colocalization between distinct causal variants.

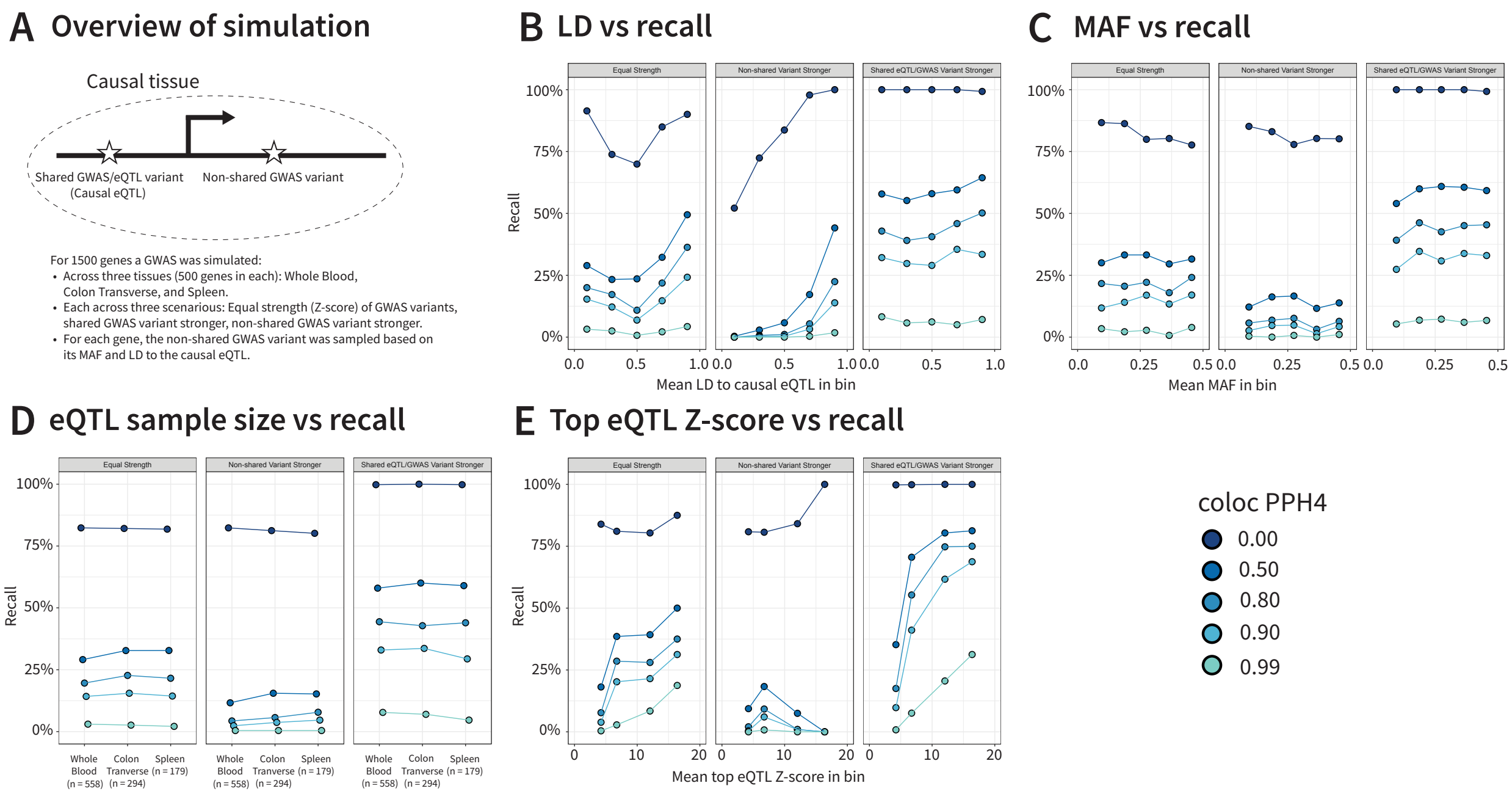

Supplementary Figure 4. The effect of genetic architecture on recall. A. Simulation framework. For each gene, we simulated two causal GWAS variants: one variant shared with the causal eQTL, assumed to be the top eQTL, and one non-shared GWAS variant sampled to vary in LD with the causal eQTL, MAF, and effect size. Simulations were performed across Whole Blood, Colon Transverse, and Spleen under three effect-size scenarios: equal strength of the shared and non-shared GWAS variants, stronger signal of the non-shared GWAS variant, and stronger signal of the shared eQTL/GWAS variant. **B-E.** Recall was estimated after binning gene-tissue pairs by mean LD between the non-shared GWAS variant and the causal eQTL (**B**), MAF of the non-shared GWAS variant (**C**), eQTL sample size (**D**), and top eQTL Z-score (**E**). Columns show the three strength scenarios. Colors indicate coloc PPH4 filtering thresholds.

Supplementary Note 5: cTWAS shows similarly high levels of FSR

To explore whether more recent TWAS methods designed to adjust for local genetic confounding reduce FSR, we repeated the protein expression analysis using causal TWAS (cTWAS) [3]. In contrast to FUSION, cTWAS combines TWAS with statistical fine-mapping to jointly model molecular traits and nearby genetic variants within a genomic region. This allows cTWAS to distinguish putative gene-mediated effects from associations driven by correlated local variants or nearby genes, and to assign each gene-tissue pair a posterior inclusion probability (PIP).

We quantified the FSR of cTWAS similar to our previous analysis, by first taking a majority vote within tissue categories and subsequently taking the majority vote across tissues to assign the direction for each gene. For the unfiltered cTWAS gene Z-scores of the proteins (Figure S5A), we observed similar levels of discordance to the FUSION estimates (24% (95% CI: 22-27%) vs. 23% (95% CI: 20-25%) for cTWAS and FUSION, respectively).

Next, we filtered each gene-tissue association from cTWAS by the PIP, in a similar way to how we filtered FUSION estimates by coloc PPH4. We then calculated FSR and recall for the associations at the gene-tissue category and gene level (Figure S5B). We found that filtering of the PIP moderately reduced FSR but was accompanied by a substantial drop in recall. Together, these results show that the high FSR observed in our protein-expression analysis is not specific to FUSION but persists even when using a more recent TWAS method designed to account for local genetic confounding.

Finally, to test if TWAS methods that model all tissues jointly improve FSR, we ran multigroup cTWAS (M-cTWAS) [4]. M-cTWAS extends cTWAS to jointly analyze multiple QTL datasets across tissues and modalities (e.g., expression and splice QTLs), using PIPs to prioritize the gene-tissue associations or variants that best explain the GWAS association. To evaluate FSR, we ran M-cTWAS jointly across all available tissue expression models for each protein-coding gene in our analysis. Since M-cTWAS produces tissue-specific gene PIPs, we assigned the gene's direction using the effect estimate by the tissue with the highest PIP. We then varied the PIP threshold used to retain genes and calculated the corresponding FSR and recall at each threshold (Figure S5C). We found that M-cTWAS showed similar levels of FSR and recall compared to FUSION and cTWAS, showing that high FSR persists even when tissue-specific expression models are analyzed jointly.

A limitation of this analysis is that we did not estimate cTWAS prior parameters using loci across the genome. Instead, for computational feasibility across the 3,000 protein encoding genes, we restricted each run to a local region containing the focal gene's LD block and two LD blocks on either side (Methods), corresponding to regions of 2-4 Mb. Because cTWAS estimates prior parameters from the regions included in the analysis, restricting to local regions may lead to less accurate priors, which could affect PIP estimation.

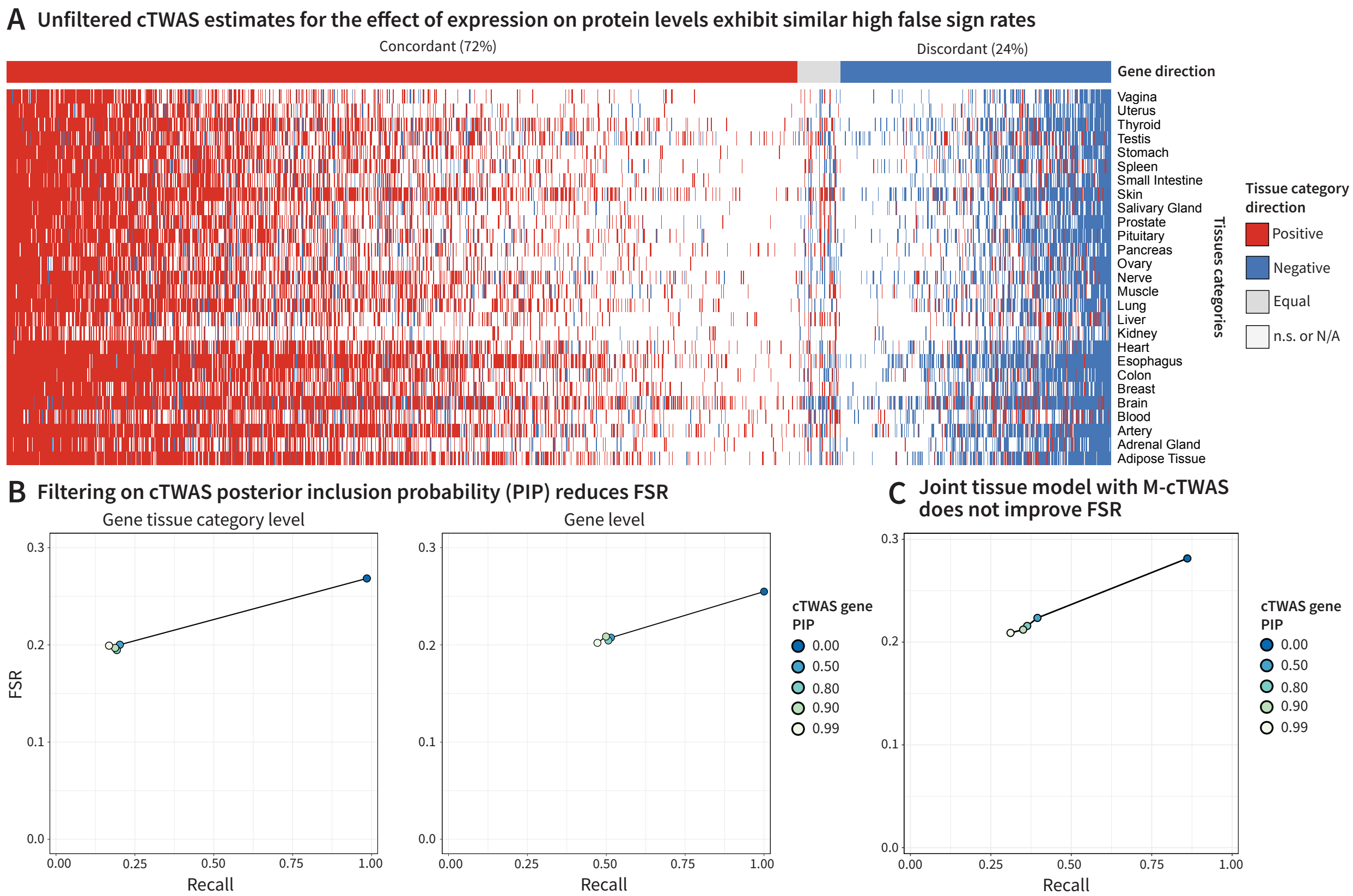

Supplementary Figure 5. cTWAS and M-cTWAS show similarly high levels of false sign rates. A. Heatmap of direction-of-effect estimates from unfiltered cTWAS gene Z-scores for protein expression associations. Columns represent protein-coding genes and rows represent tissue categories. For each gene-tissue category, directions were assigned by majority vote across tissues within the category, with positive effects shown in red, negative effects in blue, equal evidence in gray, and non-significant or unavailable estimates in white. The top annotation shows the final gene-level direction assigned by majority vote (50%) across tissue categories. **B.** FSR versus recall for cTWAS after filtering gene-tissue associations by cTWAS PIP. Results are shown at the gene-tissue category level (left) and gene level (right) across PIP thresholds of 0, 0.5, 0.8, 0.9, and 0.99. **C.** FSR versus recall for M-cTWAS. For each gene, direction was assigned using the tissue-specific effect estimate with the highest PIP, and results are shown across the same PIP thresholds as for **B**.

Supplementary Note 6: Discordant biological examples for complex traits

We explored some of the discordant associations we observed between LoF burden tests and TWAS.

GCK and Blood Glucose

The first example was the association between GCK and blood glucose levels, which had discordant TWAS signs in all tissues. GCK encodes glucokinase (GCK), an enzyme that catalyzes the first step in glycolysis and acts as a glucose sensor in various tissues. LoF variants in GCK are associated with increased blood glucose levels [5–7]. The mechanism is thought to occur via pancreatic beta cells, where the inability to detect glucose levels results in lower insulin secretion and hyperglycemia [8]. Activating mutations in GCK cause hypoglycemia due to increased insulin secretion [9]. Therefore, for this gene, we expect TWAS to have a negative Z-score, since increased GCK expression should be associated with reduced glucose levels. There is a strong GWAS signal present at the GCK locus, but no eQTL signal in the pancreas, resulting in no significant TWAS association in the tissue.

There were significant TWAS associations in 17 tissue groups for GCK and glucose, all of which were in the wrong direction. Tibial nerve tissue had the most significant eQTL for this gene, so we analyzed this tissue further. In addition, we observed striking colocalization with thyroid tissue, so we analyzed this tissue as well. We identified no credible sets in pancreas, one credible set in thyroid, and one credible set in tibial nerve tissue. We concurrently identified 9 credible sets for blood glucose levels at the locus.

There was no evidence for colocalization between the pancreas Bayes factors and the GWAS signal due to a lack of eQTL signal in the pancreas (PPH2 > 0.8 for all credible sets). The credible set from the tibial nerve eQTL summary statistics had some evidence of colocalization with the second strongest GWAS signal (PPH4 = 0.51). The thyroid credible set colocalized with a high posterior probability (PPH4 = 0.99) to the primary GWAS credible set and plotting the association summary statistics against each other shows strong evidence for the same variants underlying both signals, but in the wrong direction.

CXCL2 and Neutrophil Percentage

The CXCL2 gene encodes chemokine (C-X-C motif) ligand 2 (CXCL2), a protein that acts as a chemoattractant for leukocytes during inflammation [10]. LoF variants in CXCL2 significantly reduce both neutrophil count and neutrophil percentage in blood [11, 12], consistent with the role of CXCL2 in recruiting neutrophils. We expect TWAS to have a positive Z-score, since decreased expression should reduce neutrophil percentage.

CXCL2 had significant TWAS associations with neutrophil percentage in 11 tissue groups, of

which only one (Small Intestine) was in the expected direction. The most significant eQTL for CXCL2 was in tibial nerve tissue. However, processing the tibial nerve eQTL summary statistics with SuSiE failed to identify any credible sets. In contrast, SuSiE identified seven credible sets from the GWAS summary statistics for neutrophil percentage at the locus. We tested the approximate Bayes factors from the eQTL summary statistics against the credible sets from the GWAS. The primary credible set from the GWAS colocalized with the eQTL data with high posterior probability (PPH4 = 0.87). Similar to GCK and glucose, the summary statistics visually show strong evidence for an association, but in the wrong direction.

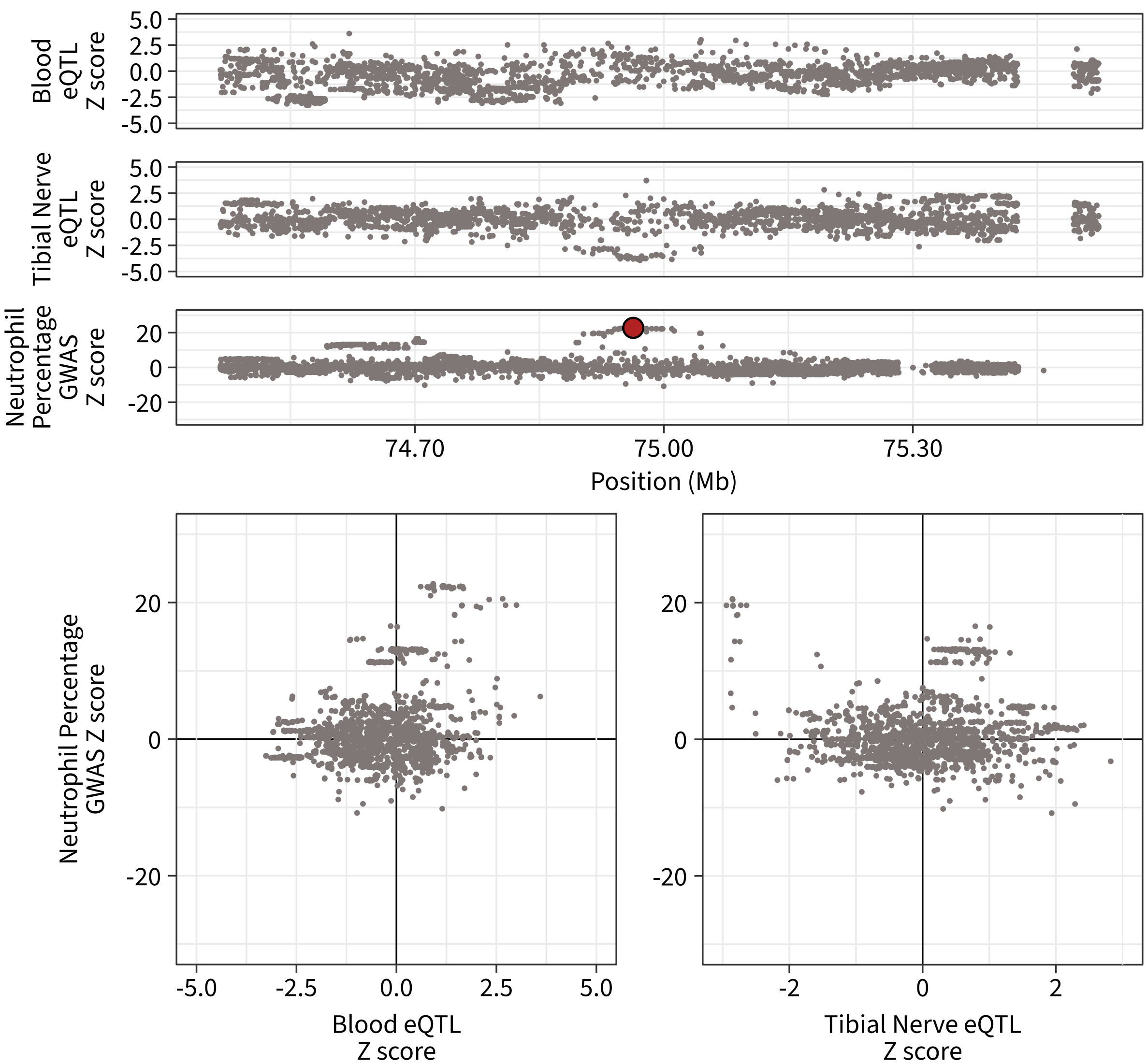

Supplementary Figure 6. TWAS associations at the CXCL2 Locus. CXCL2 is a chemokine that increases neutrophil levels. In whole blood, which is the closest to a causal tissue, there is a hint of a positive relationship, which is expected. In contrast, in tibial nerve, we observe a strong negative relationship that has both colocalization signal and results in a negative TWAS Z-score.

GMPR and Mean Corpuscular Hemoglobin

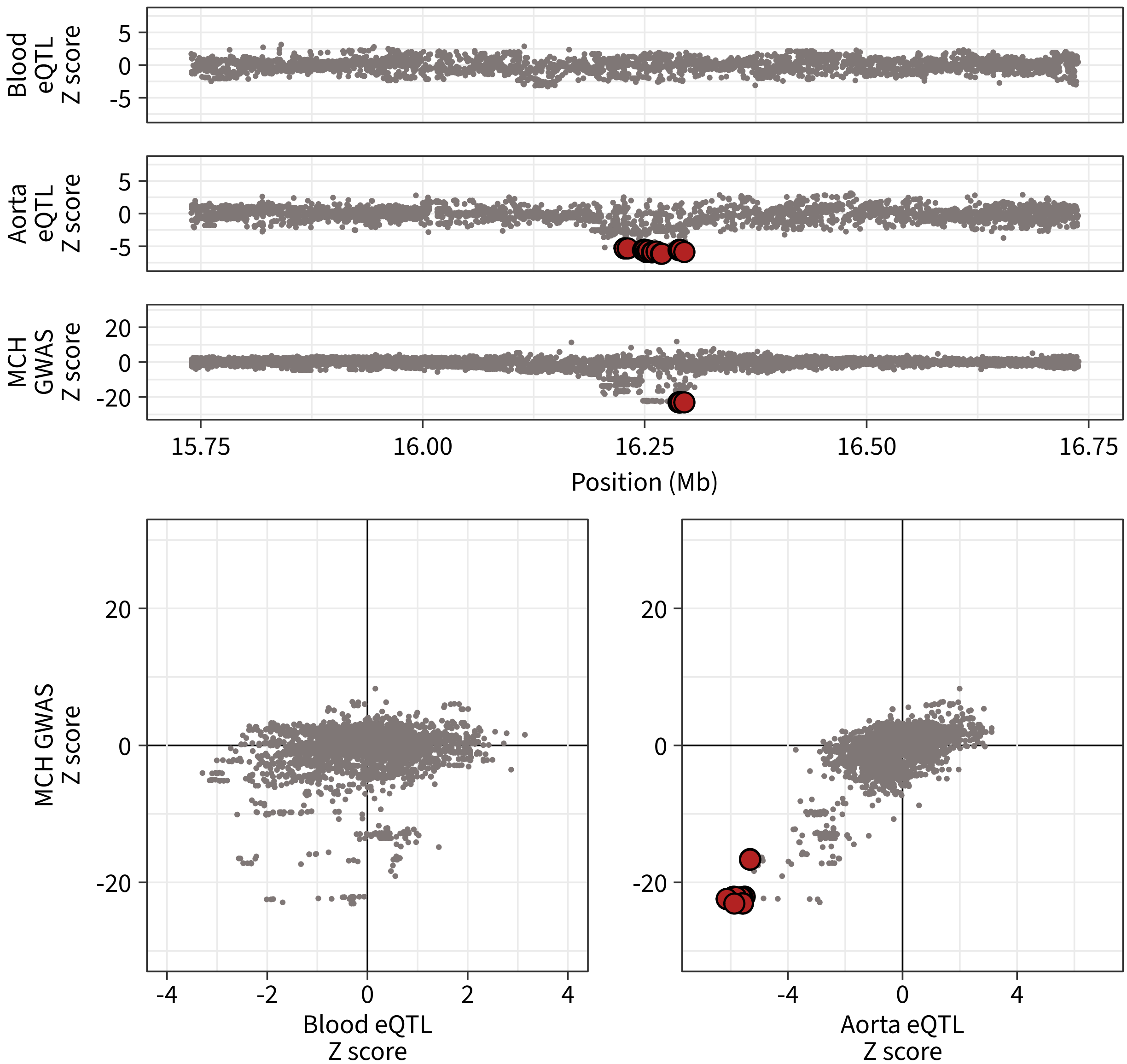

Supplementary Figure 7. TWAS associations at the GMPR Locus. GMPR deletion is associated with increased hemoglobin concentration, so we expect TWAS associations to be negative. TWAS reports a positive Z-score in blood, but a negative Z-score in aorta. The evidence for colocalization is much stronger in the aorta.

The GMPR gene encodes GMP reductase (GMPR), which has previously been associated with hemoglobin levels [11–13]. LoF variants in GMPR are associated with increased mean corpuscular hemoglobin (MCH). GMPR may be involved in cell cycle regulation [14], which is known to have a negative effect on MCH [15]. We expect TWAS to have a negative Z-score, since decreased GMPR is associated with increased hemoglobin levels.

GMPR had significant TWAS associations with MCH in 14 tissue groups, of which blood, brain, and kidney showed the correct direction-of-effect. Blood is the closest tissue to the trait

available in GTEx, and it is reassuring to see the correct direction-of-effect. The most significant eQTLs were present in artery tissue, which were significantly associated in TWAS but in the wrong direction. We analyzed eQTL summary statistics from blood and aorta to better understand this inconsistency. We identified no credible sets in blood, and a single credible set in the aorta. We identified nine credible sets for MCH at the locus. The blood eQTL showed no evidence of colocalization and visually did not show any evidence of association either. In contrast, the credible set from the aorta colocalized strongly with the primary GWAS credible set (PPH4 = 0.93), and the same variants from both the eQTL and GWAS summary statistics seem to explain the signal, but in the wrong direction

ADAMTS17 and Height

The ADAMTS17 gene encodes ADAM metallopeptidase with thrombospondin type 1 motif, 17 (ADAMTS17). LoF variants in this gene are associated with a reduction in height [11, 12]. Homozygous LoF genotypes are associated with Weill-Marchesani syndrome, which is a Mendelian disorder that presents with short stature [16]. Therefore, we expect TWAS to have a positive Z-score, since decreased expression should reduce height.

ADAMTS17 only had significant TWAS results in two tissue groups, both of which were in the wrong direction. The strongest eQTL was present in thyroid tissue, but this model was not available for TWAS. Of the two tissue groups with models (heart and brain), the atrial appendage tissue had the most significant eQTL, which we analyzed further. SuSiE did not detect any credible sets with the eQTL summary statistics, so we proceeded with approximate Bayes factors instead. We identified ten credible sets for height at the locus, of which none colocalized with the heart eQTL summary statistics (PPH3 > 0.99 for all credible sets). This is an example of an association that would be filtered out by using colocalization or SuSiE after TWAS to filter gene-trait pairs. However, visually, the variants are strikingly correlated, which explains the TWAS Z-score.

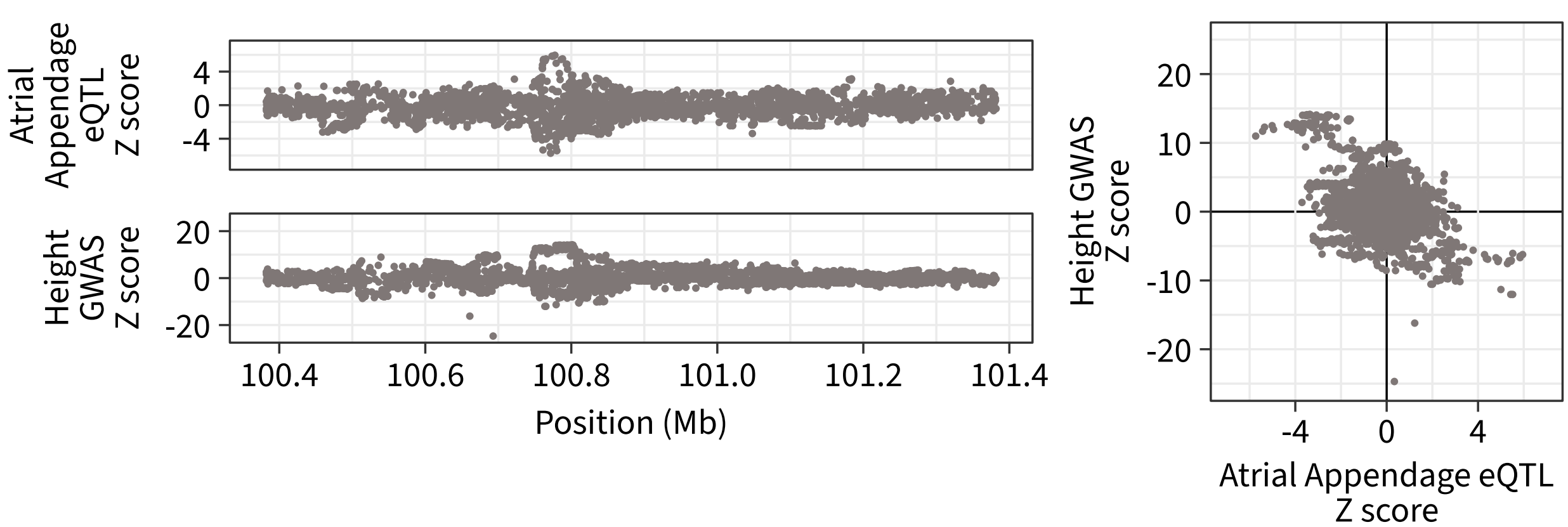

Supplementary Figure 8. TWAS associations at the ADAMTS17 Locus. ADAMTS17 is a positive modulator of height, so TWAS is expected to return positive Z-scores. However, TWAS returned a negative Z-score in heart tissue, although this association would be filtered by colocalization.

Supplementary Note 7: Linear model of FSR across gene-trait pairs

We used a regression strategy to test if any gene-level features explained why TWAS assigned a confidently incorrect sign at certain loci. We used four simple features: (1) the number of genes in a 20 kilobase region around the TSS, (2) the gene constraint as measured by $\log_{10}(s_{\text{het}})$, (3) the mean recombination rate in the 1 megabase window around the TSS in the EUR superpopulation from the 1000 genomes project [17], and (4) the mean of the *cis*-heritability estimates reported in the constructed FUSION TWAS models.

TWAS performance may also be explained by the power of the GWAS analysis. For this reason, we included the sample size of the GWAS for each trait as a covariate. Additionally, we have previously estimated trait-level monotonicity, which describes the average monotonicity of gene dosage response curves [11]. Since more non-monotone gene dosage response curves might result in more discordance of gene-level effect estimates, we also included our monotonicity estimates as covariates.

We analyzed the TWAS Z-scores that were calculated from the "filtered" gene list. We calculated the Bonferroni absolute Z-score threshold for a two-sided test based on the number of Z-scores available across all tissues and gene-trait pairs. We then used a majority vote strategy to assign a sign to each gene in its tissue group. For example, we assigned one sign to TWAS in the brain tissue group rather than using 13 brain tissues available in GTEx.

We counted the total number of significant Z-scores (N_{ij}) and the subset of those that were confidently in the wrong direction (Y_{ij}) for the ith gene and jth trait. Then, we modeled the counts as

$$Y_{ij} \sim \text{Binomial}\left(N_{ij}, p_{ij}\right) \ ,$$

with the link function

$$\text{logit}\left(p_{ij}\right) = \mathbf{X}_{ij}^{\top}\boldsymbol{\beta} + u_j \, ,$$

where

$$u_i \sim \mathcal{N}\left(0, \sigma_u^2\right) \ .$$

Here, $\mathbf{X}_{ij}$ represent the values of the various covariates that may explain the incorrect sign from TWAS, and $\boldsymbol{\beta}$ represents the vector of fixed effects. The random effect u_j for each trait should capture any trait-specific effects on TWAS that are not explained by GWAS power or monotonicity. This is a generalized linear mixed effects model in the binomial family with a logit link function, which can be fit using the `lme4` package in R. Specifically, using the formula language from `lme4`, we fit the model

$$\begin{aligned}\mathbb{M} : \texttt{cbind(negatives, total)} \sim \texttt{density\_20kb} + \texttt{log10\_s\_het} + \texttt{mean\_recomb\_rate} \\ + \texttt{mean\_hsq} + \texttt{GWAS\_N} + \texttt{monotonicity} + \texttt{(1|trait)} \, ,\end{aligned}$$

and compared it to a null model with trait-specific confounders,

$$\mathbb{M}_0 : \texttt{cbind(negatives, total)} \sim \texttt{GWAS\_N} + \texttt{monotonicity} + \texttt{(1|trait)}.$$

Using an analysis of variance (ANOVA), we found that $\mathbb{M}$ was a significantly better fit than $\mathbb{M}_0$ ($p = 3.6 \times 10^{-4}$), suggesting that gene density, gene constraint, *cis*-heritability, and mean recombination rate likely explain some of incorrect sign assignment. Using likelihood-based inference, the coefficients for gene constraint and mean recombination rate were significant in $\mathbb{M}$. A unit increase in $\log_{10}(s_{\text{het}})$ was estimated to reduce the odds of an incorrect assignment to 0.83 ($p = 0.01$), while a unit increase in $\log_{10}(\texttt{mean\_recomb\_rate})$ was estimated to increase the odds of an incorrect assignment to 1.51 ($p = 0.001$). This suggests that constrained genes tend to have fewer TWAS sign errors, and regions with higher mean recombination rate tend to have more TWAS sign errors. However, we interpreted these associations cautiously and conservatively because this analysis is based on only 383 gene-trait pairs from 186 unique genes and 68 unique traits. Therefore, we conclude that gene density, gene constraint, *cis*-heritability, and mean recombination rate may, in combination, explain some of the variation in TWAS sign inconsistency.

Supplementary Note 8: Strong anti-correlation between tissues may create false-positive evidence of colocalization

We hypothesize that much of the false-sign rate in TWAS is driven by gene-tissue associations in non-trait-relevant tissues, in which the gene has little or no causal effect on the trait, but its expression tags the same genetic variation that drives the true causal mechanism in an unobserved or underpowered trait-relevant tissue. To explore how co-regulation between a known causal tissue and other tissues affects FSR, we simulated GWAS for 1,000 genes using blood as the causal tissue, by setting the top eQTL in blood as the causal GWAS variant (Figure S9A). This simulation followed the same framework as the true-positive scenario in Supplementary Note 2, with GWAS summary statistics simulated in a 1 Mb window centered on the causal eQTL using UK Biobank LD matrices and allele frequencies. We assigned the causal GWAS variant a target Z-score of 30, reflecting the strong local GWAS signals observed in the protein summary statistics. When we evaluated FSR across all non-blood tissue categories, the estimated FSR was 21%. This suggests that a substantial fraction of false-sign TWAS associations arise from tagging tissues whose eQTL effects are anti-correlated with those in the causal blood tissue.

Next, we evaluated how false-sign rates were related to the degree of co-regulation between non-blood tissues and the causal blood tissue. For each gene-tissue pair, we calculated the correlation between the genetically predicted expression in that tissue and the corresponding predicted expression in blood (Methods). We then grouped gene-tissue pairs into 20 bins according to the tissue's correlation with blood and estimated the FSR within each bin (Figure S9B, left). As expected, we found that FSR decreased with increasing correlation between genetically predicted expression in non-blood tissues and the causal blood tissue. We observed a similar pattern after restricting to gene-tissue associations with coloc PPH4 > 0.5 (Figure S9B, right). Notably, while colocalization enriched for gene-tissue pairs with strong positive correlation to the causal blood tissue, it also enriched for strongly anti-correlated gene-tissue pairs. This pattern is consistent with our hypothesis that discordant signals in non-trait-relevant tissues can arise when GWAS and eQTL signals are driven by distinct variants in very high LD, which neither TWAS nor colocalization can reliably distinguish. In such a setting, the non-blood tissues may carry eQTL effects in the opposite direction from blood, yet still appear to colocalize because their eQTL tags the causal eQTL in blood through very high LD.

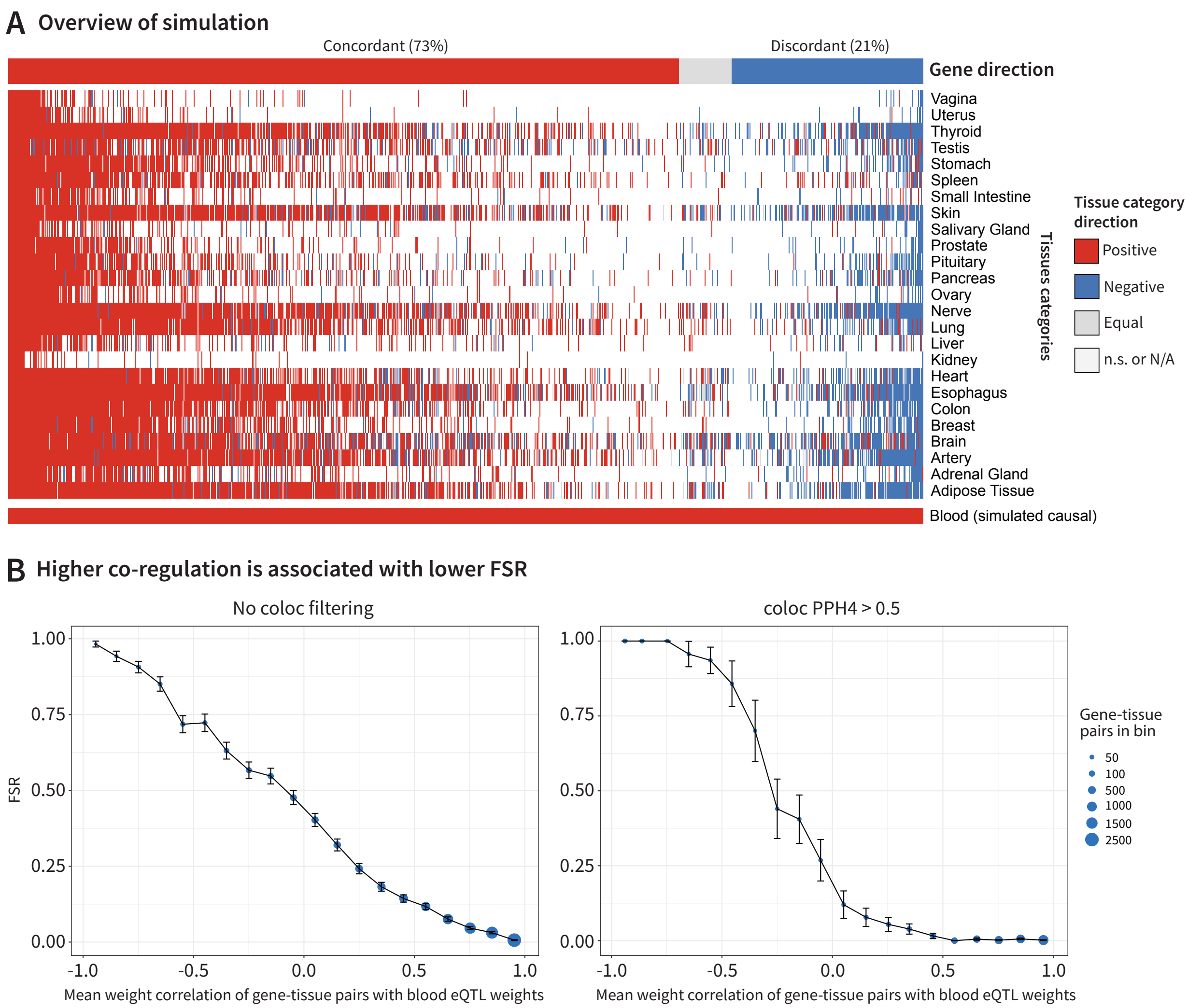

Supplementary Figure 9. Anti-correlated tagging tissues can produce false-positive evidence of colocalization. A. Simulation framework used to evaluate FSR when blood is the causal tissue. For each of 1,000 genes, we simulated GWAS summary statistics by setting the top blood eQTL as the causal GWAS variant. The gene direction was calculated as previously from a majority vote across gene-tissue categories but excluding blood. **B.** Gene-tissue pairs from non-blood tissues were binned by the correlation between their genetically predicted expression and genetically predicted expression in blood and FSR was calculated within each bin. The point size indicates the number of gene-tissue pairs within the bin. Error bars indicate ±1 standard error for the FSR estimate.

Supplementary Tables

GTEx tissue	Tissue category
Adipose Subcutaneous	Adipose Tissue
Adipose Visceral Omentum	Adipose Tissue
Adrenal Gland	Adrenal Gland
Artery Aorta	Artery
Artery Coronary	Artery
Artery Tibial	Artery
Brain Amygdala	Brain
Brain Anterior cingulate cortex BA24	Brain
Brain Caudate basal ganglia	Brain
Brain Cerebellar Hemisphere	Brain
Brain Cerebellum	Brain
Brain Cortex	Brain
Brain Frontal Cortex BA9	Brain
Brain Hippocampus	Brain
Brain Hypothalamus	Brain
Brain Nucleus accumbens basal ganglia	Brain
Brain Putamen basal ganglia	Brain
Brain Spinal cord cervical c-1	Brain
Brain Substantia nigra	Brain
Breast Mammary Tissue	Breast
Colon Sigmoid	Colon
Colon Transverse	Colon
Esophagus Gastroesophageal Junction	Esophagus
Esophagus Mucosa	Esophagus
Esophagus Muscularis	Esophagus
Heart Atrial Appendage	Heart
Heart Left Ventricle	Heart
Kidney Cortex	Kidney
Liver	Liver
Lung	Lung
Minor Salivary Gland	Salivary Gland
Muscle Skeletal	Muscle
Nerve Tibial	Nerve
Ovary	Ovary
Pancreas	Pancreas
Pituitary	Pituitary
Prostate	Prostate
Skin Not Sun Exposed Suprapubic	Skin
Skin Sun Exposed Lower leg	Skin
Small Intestine Terminal Ileum	Small Intestine
Spleen	Spleen
Stomach	Stomach
Testis	Testis
Thyroid	Thyroid
Uterus	Uterus
Vagina	Vagina
Whole Blood	Blood

Supplementary Table 1. Tissues from GTEx grouped into tissue categories.

UKB Field ID	Trait	UKB Field ID	Trait
102	Pulse rate automated reading	46	Hand grip strength (left)
20019	Speech-reception-threshold (SRT) estimate (left)	47	Hand grip strength (right)
20021	Speech-reception-threshold (SRT) estimate (right)	48	Waist circumference
21001	Body mass index (BMI)	49	Hip circumference
21002	Weight	50	Standing height
23099	Body fat percentage	5084	Spherical power (right)
23102	Whole body water mass	5085	Spherical power (left)
23105	Basal metabolic rate	5086	Cylindrical power (left)
23106	Impedance of whole body	5087	Cylindrical power (right)
2754	Age at first live birth	5088	Astigmatism angle (right)
2764	Age at last live birth	5089	Astigmatism angle (left)
30000	White blood cell (leukocyte) count	78	Heel bone mineral density (BMD)
30010	Red blood cell (erythrocyte) count	30600	Albumin
30020	Hemoglobin concentration	30700	Creatinine
30030	Haematocrit percentage	30610	Alkaline phosphatase
30040	Mean corpuscular volume	30620	Alanine aminotransferase
30050	Mean corpuscular hemoglobin	30630	Apolipoprotein A
30060	Mean corpuscular hemoglobin concentration	30640	Apolipoprotein B
30070	Red blood cell (erythrocyte) distribution width	30650	Aspartate aminotransferase
30080	Platelet count	30660	Direct bilirubin
30090	Platelet crit	30670	Urea
30100	Mean platelet (thrombocyte) volume	30680	Calcium
30110	Platelet distribution width	30690	Cholesterol
30120	Lymphocyte count	30710	C-reactive protein
30130	Monocyte count	30720	Cystatin C
30140	Neutrophil count	30730	Gamma glutamyltransferase
30180	Lymphocyte percentage	30740	Glucose
30190	Monocyte percentage	30750	Glycated hemoglobin (HbA1c)
30200	Neutrophil percentage	30760	HDL cholesterol
30210	Eosinophil percentage	30770	IGF-1
30220	Basophil percentage	30780	LDL direct
30240	Reticulocyte percentage	30790	Lipoprotein A
30250	Reticulocyte count	30800	Oestradiol
30260	Mean reticulocyte volume	30810	Phosphate
30270	Mean sphered cell volume	30820	Rheumatoid factor
30280	Immature reticulocyte fraction	30830	SHBG
30290	High light scatter reticulocyte percentage	30840	Total bilirubin
30300	High light scatter reticulocyte count	30850	Testosterone
3062	Forced vital capacity (FVC)	30860	Total protein
3063	Forced expiratory volume in 1-second (FEV1)	30870	Triglycerides
3064	Peak expiratory flow (PEF)	30880	Urate
4079	Diastolic blood pressure automated reading	30890	Vitamin D
4080	Systolic blood pressure automated reading		

Supplementary Table 2. Complex traits used for analysis.

References

1. Morrison, J. *GWASBrewer: Simulate Realistic GWAS Summary Statistics* R package version 0.3.0.0158 (2023). https://github.com/jean997/GWASBrewer.
2. Morrison, J. GWASBrewer: An R Package for Simulating Realistic GWAS Summary Statistics. *Genetic Epidemiology* **49,** e22594 (2025).
3. Zhao, S., Crouse, W., Qian, S., Luo, K., Stephens, M. & He, X. Adjusting for genetic confounders in transcriptome-wide association studies improves discovery of risk genes of complex traits. *Nature Genetics* **56,** 336–347 (2024).
4. Qian, S., Luo, K., Sun, X., Crouse, W., Liang, L., Gu, J., *et al.* Integrating multi-omics and multi-context QTL data with GWAS reveals the genetic architecture of complex traits and improves the discovery of risk genes. *medRxiv* (2026).
5. Froguel, P., Vaxillaire, M., Sun, F., Velho, G., Zouali, H., Butel, M. O., *et al.* Close linkage of glucokinase locus on chromosome 7p to early-onset non-insulin-dependent diabetes mellitus. *Nature* **356,** 162–164 (1992).
6. Hattersley, A. T., Turner, R. C., Permutt, M. A., Patel, P., Tanizawa, Y., Chiu, K. C., *et al.* Linkage of type 2 diabetes to the glucokinase gene. *The Lancet* **339,** 1307–1310 (1992).
7. Njølstad, P. R., Søvik, O., Cuesta-Muñoz, A., Bjørkhaug, L., Massa, O., Barbetti, F., *et al.* Neonatal Diabetes Mellitus Due to Complete Glucokinase Deficiency. *New England Journal of Medicine* **344,** 1588–1592 (2001).
8. Meglasson, M. D. & Matschinsky, F. M. New perspectives on pancreatic islet glucokinase. *American Journal of Physiology-Endocrinology and Metabolism* **246,** E1–E13 (1984).
9. Christesen, H. B. T., Tribble, N. D., Molven, A., Siddiqui, J., Sandal, T., Brusgaard, K., *et al.* Activating glucokinase (GCK) mutations as a cause of medically responsive congenital hyperinsulinism: prevalence in children and characterisation of a novel GCK mutation. *European Journal of Endocrinology* **159,** 27–34 (2008).
10. Griffith, J. W., Sokol, C. L. & Luster, A. D. Chemokines and Chemokine Receptors: Positioning Cells for Host Defense and Immunity. *Annual Review of Immunology* **32,** 659–702 (2014).
11. Milind, N., Smith, C. J., Zhu, H., Gjorgjieva, T., Spence, J. P. & Pritchard, J. K. Buffering of gene dosage response curves for human complex traits. *Cell Genomics* **6,** 101221 (2026).
12. Karczewski, K. J., Solomonson, M., Chao, K. R., Goodrich, J. K., Tiao, G., Lu, W., *et al.* Systematic single-variant and gene-based association testing of thousands of phenotypes in 394,841 UK Biobank exomes. *Cell Genomics* **2,** 100168 (2022).
13. Van Hout, C. V., Tachmazidou, I., Backman, J. D., Hoffman, J. D., Liu, D., Pandey, A. K., *et al.* Exome sequencing and characterization of 49,960 individuals in the UK Biobank. *Nature* **586,** 749–756 (2020).
14. Schmunk, L. J. Functional Investigation of Genetic Determinants of Red Blood Cell Traits. PhD thesis (University of Cambridge, 2020).
15. Ota, M., Spence, J. P., Zeng, T., Dann, E., Milind, N., Marson, A., *et al.* Causal modelling of gene effects from regulators to programs to traits. *Nature* **650,** 399–408 (2026).
16. Marzin, P., Rondeau, S., Alessandri, J.-L., Dieterich, K., Goff, C. l., Mahaut, C., *et al.* Weill-Marchesani syndrome: natural history and genotype-phenotype correlations from 18 news cases and review of literature. *Journal of Medical Genetics* **61,** 109–116 (2024).
17. Spence, J. P. & Song, Y. S. Inference and analysis of population-specific fine-scale recombination maps across 26 diverse human populations. *Science Advances* **5,** eaaw9206 (2019).
