# Supplementary material for: High false sign rates in transcriptome-wide association studies
Source: Nat Genet. 2026 Oct 22;58(11):3004–12. doi: 10.1038/s41588-026-02796-4 (PMC13754616; doi:10.1038/s41588-026-02796-4)
Supplement: Supplementary file 3 [file 41588_2026_2796_MOESM3_ESM.pdf]

nature portfolio

Peer Review File

High false sign rates in transcriptome-wide association studies

Corresponding Author: Mr Peter Gerlach

Version 0:

Decision Letter:

10th Mar 2026

Dear Mr Gerlach,

Your Article, "High false sign rates in transcriptome-wide association studies" has now been seen by 2 referees. You will see from their comments below that while they find your work of interest, some important points are raised. We are interested in the possibility of publishing your study in Nature Genetics, but would like to consider your response to these concerns in the form of a revised manuscript before we make a final decision on publication.

We therefore invite you to revise your manuscript taking into account all reviewer and editor comments. Please highlight all changes in the manuscript text file. At this stage we will need you to upload a copy of the manuscript in MS Word .docx or similar editable format.

We are committed to providing a fair and constructive peer-review process. Do not hesitate to contact us if there are specific requests from the reviewers that you believe are technically impossible or unlikely to yield a meaningful outcome.

When revising your manuscript:

*1) Include a “Response to referees” document detailing, point-by-point, how you addressed each referee comment. If no action was taken to address a point, you must provide a compelling argument. This response will be sent back to the referees along with the revised manuscript.

*2) If you have not done so already please begin to revise your manuscript so that it conforms to our Article format instructions, available
here.
Refer also to any guidelines provided in this letter.

*3) Include a revised version of any required Reporting Summary: https://www.nature.com/documents/nr-reporting-summary.pdf
It will be available to referees (and, potentially, statisticians) to aid in their evaluation if the manuscript goes back for peer review.
A revised checklist is essential for re-review of the paper.

Please be aware of our guidelines on digital image standards.

EXTENDED DATA FIGURES

When re-submitting your manuscript, please ensure that any supplementary figures and tables that are crucial to the manuscript’s conclusions are converted into Extended Data figures and tables to increase visibility of these data. Extended Data figures and tables are online-only (present in the online PDF and full-text HTML versions of the paper), peer-reviewed display items that provide essential background to the article but are not included in the main article due to space constraints. A maximum of ten Extended Data display items (figures and tables) is permitted.

Please use the link below to submit your revised manuscript and related files:

Link Redacted

Note: This URL links to your confidential home page and associated information about manuscripts you may have submitted, or that you are reviewing for us. If you wish to forward this email to co-authors, please delete the link to your homepage.

We hope to receive your revised manuscript within four to eight weeks. If you cannot send it within this time, please let us know.

Please do not hesitate to contact me if you have any questions or would like to discuss these revisions further.

Nature Genetics is committed to improving transparency in authorship. As part of our efforts in this direction, we are now requesting that all authors identified as 'corresponding author' on published papers create and link their Open Researcher and Contributor Identifier (ORCID) with their account on the Manuscript Tracking System (MTS), prior to acceptance. ORCID helps the scientific community achieve unambiguous attribution of all scholarly contributions. You can create and link your ORCID from the home page of the MTS by clicking on 'Modify my Springer Nature account'. For more information please visit please visit www.springernature.com/orcid.

We look forward to seeing the revised manuscript and thank you for the opportunity to review your work.

Sincerely,

Margot Brandt, PhD
Senior Editor
Nature Genetics
https://orcid.org/0000-0002-9434-794X

Referee expertise:

Referee #1: statistical genetics

Referee #2: statistical genetics/TWAS

Reviewers' Comments:

Reviewer #1 (Remarks to the Author):

Gerlach and colleagues present a very compelling set of analyses that directs caution toward the direction of effect of the gene-disease association statistics generated by TWAS. The authors cleverly associate gene expression prediction models with protein measurements, as a valid hypothesis is that variants that increase gene expression should also increase protein expression. This study has some particularly impactful results such as

• Quantifying the proportion of gene-disease associations (23%-33%) with the incorrect direction of effect determined by TWAS. This leads to incorrect biological interpretations regarding if more or less of the gene increases or decreases disease risk.
• 85.3% of protein-encoding genes showed at least one TWAS-significant nearby gene, suggesting that non-causal tagging signals are highly prevalent.
• While purifying selection acting more strongly on trait-relevant tissues, stronger TWAS signals tend to be discovered in non-trait relevant tissues. This is an impactful statement because for many years, general advice regarding TWAS has been to exploit proxy tissues when needed as they will likely tag the causal SNP-trait effects and permit the recovery of the gene-disease association. However, this is not helpful if the sign is wrong.

The authors also propose coloc-based filtering and the usage of trait relevant tissue to remove the sign issue, which serves as a workable solution (if the sample size is large enough that coloc works well and if the trait relevant tissue is known which may not be the case), but ultimately limits the power to detect disease-critical genes as so few genes have high posterior probabilities of colocalization. Overall, I think this study was concise and makes a clearly compelling and cautionary point. However, there needs to be further analysis done in simulations to back up the claims of the authors regarding selection and co-regulation between tissues to quantitatively assess their impact on FSR. Also, there needs to be further investigation with state-of-the-art variations of TWAS to understand just how pervasive this FSR problem is.

Major Comments

1. The majority vote approach taken on lines 84-85 obscures the variability of the directions of effect. It would be interesting to quantify the gene-level variability of FSR across tissues where the gene is expressed. This might allow even stronger claims about FSR, as the analysis would reveal genes with high variability even in the scenario where the majority vote indicates concordant directions of effect.
2. While there were several hypotheses regarding what gene-level architectures give rise to FSR, the results would be more compelling if the authors could recapitulate those statements using simulations of gene and corresponding protein expression. Specifically, having simulations of different causal variant architectures (i.e., shared causal variant(s) between

gene and protein traits versus independent causal variant(s) or different causal variant(s) in LD) could add more support the coloc-based filtering strategy. The analysis on lines 158-168 partially gets at this question, but analysis of simulated genetic architectures would be more compelling than guessing and testing different gene-level characteristics in an empirical linear model.

3. Related to the comment above, simulations could help understand the FSR in the context of tagging tissues, which have a strong genetic correlation with the causal tissue, but do not harbor the specific variants or specific effect sizes that gave rise to the protein trait. In simulations, how does the degree of co-regulation between tissues (i.e., correlation of eQTL effect sizes) change the likelihood of FSR?

4. The interpretation of stronger TWAS signals being discovered in non-trait relevant tissues because of purifying selection acting more strongly on trait-relevant tissues is a very impactful statement. Could the authors use simulations to show the degree of selection that corresponds to a particular increase in FSR, for instance, using realistic estimates of selection across the transcriptome?

5. It would be good if the authors could provide guidelines to the reader such as how varying the sample size of the eQTL study and separately the GWAS study affect the FSR vs recall rates during the coloc-based filtering step, based on empirical data or simulated data. Under what scenarios does the coloc or coloc-susie filtering step perform worse than the results presented in Figure 2B? For example, how do the effect sizes of the causal variants, the allele frequencies, and the average LD between the causal variants affect the coloc-filtering results?

6. Do TWAS frameworks that model uncertainty of predicted expression have decreased FSR? Or is the issue more about the (un)stated assumptions of TWAS? In a tagging tissue at infinite sample size, there is perfect certainty regarding the eQTL effects but because they only tag the causal effects in the correct tissue, FSR will still occur. At finite sample size, will models that consider the uncertainty of predicted expression improve the FSR?

7. Do recent TWAS methods that analyze association statistics across tissues improve FSR? There have been some recent methods published that the authors should compare their FSR calculations to:

Feng et al 2021 PLoS Genetics https://pubmed.ncbi.nlm.nih.gov/33831007/

Barbeira et al 2019 PLoS Genetics https://pubmed.ncbi.nlm.nih.gov/30668570/

Hu et al 2019 Nature Genetics https://pmc.ncbi.nlm.nih.gov/articles/PMC6788740/

8. Do methods that integrate TWAS with causal inference such as causal-TWAS (cTWAS, Zhao et al 2024, Nature Technical Reports) reduce the FSR?

Minor Comments

1. In the early paragraphs of the manuscript, it was not immediately clear that protein levels were being used as the complex traits in the GWAS and that gene expression levels were being used to build the predicted expression models. At first, it seemed like protein expression models were being built and then associated with GWAS sumstats for complex traits/diseases (i.e., a PWAS). The authors should clarify earlier in the introduction main text that GTEx data (with a report of the number of tissues and genes/tissue) is being used to construct the genetic prediction models. It is mentioned in Figure 1 and in the caption, but it should appear in the main text as well, probably near line 75 when the UKB-PPP was first introduced. Related to this, line 68 was confusing because “TWAS predictions of plasma protein” imply that you are predicting protein levels, but rather what was meant was “TWAS associations of plasma protein.”

2. In the discussion of Figure 2B, I believe attributing fault to TWAS entirely for the FSR is an overstatement. As demonstrated by Figure 2B, simple visualization of the eQTL and GWAS sumstats is sufficient to see that the directions of effect are opposing, and this can be unexpected. In other words, it is not the job of TWAS to correct this direction of effect issue, but rather to report the magnitude of the cis-genetic correlation. So I think the authors need to qualify the strength of their language in reporting that this is a shortcoming of TWAS. One could argue this is a shortcoming of eQTL associations as well, since they capture the effects of tagging tissues.

3. Could the authors quantify their statement in line 152: “TWAS was strikingly confident in the incorrect association direction in multiple tissues.” Does this imply that the magnitude of the TWAS association statistic and/or the p-values were particularly significant in these discordant directions of effect?

Reviewer #2 (Remarks to the Author):

Gerlach et al. benchmark the performance of transcriptome-wide association studies (TWAS) at predicting the direction of effect of causal genes at GWAS risk loci on the GWAS trait. They use two benchmarks where both the causal gene and its direction of effect are known with fairly high confidence, one based on proteomics GWAS and the other based on exome burden-test hits. This paper is important because it is (to my knowledge) the first paper to benchmark eQTL-based methods at direction-of-effect prediction from GWAS. Direction-of-effect prediction is something that eQTL-based methods like TWAS should in theory be stronger at than causal gene identification (where co-regulation is an inherent limitation), and is highly relevant to pharmaceutical development. A secondary reason the paper is important is because it provides a new orthogonal benchmark of the performance of eQTL-based methods more generally, since as the paper notes most prior benchmarking has focused on causal gene prioritization, not direction-of-effect prediction.

Comments:

1. Though RNA and protein expresion are correlated, the correlation is far from perfect, and there is also discordance between eQTLs and pQTLs, as shown in a paper co-authored by Dr. Pritchard (Battle et al., "Impact of regulatory variation from RNA to protein", Science 2014). The analysis described in the first paragraph of the results tries to allay this concern, but does not (see point #2). This matters because if there's discordance between RNA and protein expression, the false sign rate for TWAS in the UKB-PPP analysis may look worse than it actually is, since TWAS may have the correct sign for RNA expression, but a flipped sign for protein expression, merely because of this RNA-protein expression discordance.

Fortunately, this concern does not apply to the complex-trait analysis, so the headline result of the paper (that TWAS has a high false sign rate) still stands. Even so, the authors should either conduct a sensitivity analysis to address this (though it's not clear what such an analysis would look like), or mention and rebut this concern in the Discussion. (The rebuttal being that the complex-trait analysis shows the same general result.)
2. The importance of the analysis in the first paragraph of the results is not clear. It does not "support the expectation that gene expression is generally positively associated with protein expression" since it is just showing that LoF variants are associated with decreased protein expression and duplications are associated with increased protein expression; RNA expression is not part of this analysis. Thus, statements like "we expect all confident TWAS predictions of plasma protein to be positive" and "these results support the expectation that gene expression is generally positively associated with protein expression" seem out of place here. It's also not clear that this result "makes plasma protein levels an attractive phenotype to assess the false sign rate (FSR)", as the caption to Figure 1E mentions, since it would be an attractive benchmark even without this analysis.
3. The first paragraph of the results would be easier to follow if it's explicitly explained that a) this is using the UKB-PPP, b) the analysis is looking at associations between the level of each protein and LoF variants or duplications *in/of the same protein*. This would require some restructuring since the UKB-PPP currently isn't introduced until the following paragraph.
4. TWAS associations may not have the same direction of effect across tissues. To account for this, the authors take a majority vote within each tissue, and then another majority vote across tissues. The paper would benefit from some exploration of how often direction of effects disagree within and across tissues. It would also be worthwhile to look at the false sign rate when subsetting to genes where the TWAS results are consistent across tissues, or at least mostly consistent. For instance, they could subset to genes with significant TWAS associations in at least N distinct tissue categories, and where at least M <= N of these associations are in the same direction - but there are many possible formulations. This would likely remove fewer genes than colocalization-based filtering, and would avoid the problem of selecting a single "correct" tissue: as the authors note, "identifying the relevant tissues for complex traits remains an open challenge".
5. The abstract mentions that subsetting on coloc + trait-relevant tissues reduces the false sign rate to 0% - but this is on just 40 genes. It's also not consistent when using coloc-SuSiE instead of coloc, where the false sign rate is 7%. It doesn't seem like a practical solution and doesn't feel like it belongs in the abstract. What seems much more notable - and honestly an abstract-level conclusion - is that the false sign rate in the UKB-PPP analysis was 20% *even after subsetting to trait-relevant tissues*, nearly the same as the 23% false sign rate when using all tissues. And this is in the ideal scenario where the correct tissue is known; that 3% gap would easily disappear for complex traits where there is ambiguity about the correct tissue. In other words, filtering to trait-relevant tissues has essentially no detectable effect on the ability of TWAS to predict the correct direction of effect. This seems greatly underemphasized in the paper relative to how important it is.
6. Related to point #5, it would be nice to know whether the 3% gap is a significant difference. It would be useful to calculate 95% confidence intervals for all of the false sign rates in the paper. These confidence intervals would be particularly large for the 40-gene and 58-gene groups - probably the 0% from coloc is not significantly different from the 7% from coloc-SuSiE.
7. We often focus on tissues rather than cell types for TWAS because that is where the models have been developed. But heterogeneity among the cell types that make up a tissue is an important confounder, and as the field moves towards single-cell TWAS, it would be interesting to hear the authors' thoughts on how this confounding contributes to the false sign rate issue and the extent to which single-cell data can realistically mitigate it.

Version 1:

Decision Letter:

Our ref: NG-A71591R

1st Jul 2026

Dear Dr. Gerlach,

Thank you for submitting your revised manuscript "High false sign rates in transcriptome-wide association studies" (NG-A71591R). It has now been seen by the original referees and their comments are below. The reviewers find that the paper has improved in revision, and therefore we'll be happy in principle to publish it in Nature Genetics, pending minor revisions to satisfy the referees' final requests and to comply with our editorial and formatting guidelines.

If the current version of your manuscript is in a PDF format, please email us a copy of the file in an editable format (Microsoft Word or LaTex)-- we can not proceed with PDFs at this stage.

We are now performing detailed checks on your paper and will send you a checklist detailing our editorial and formatting requirements soon. Please do not upload the final materials and make any revisions until you receive this additional information from us.

Thank you again for your interest in Nature Genetics Please do not hesitate to contact me if you have any questions.

Sincerely,

Margot Brandt, PhD
Senior Editor
Nature Genetics
https://orcid.org/0000-0002-9434-794X

Reviewer #1 (Remarks to the Author):

The authors have fully addressed my comments and concerns. I appreciate the detailed responses and new analyses that they provided. I have no additional concerns.

Reviewer #2 (Remarks to the Author):

The authors have satisfactorily addressed my concerns. Congratulations on an innovative manuscript!

INTERNAL USE ONLY AIP modules required: ["Clinical","Life Science"]

Open Access This Peer Review File is licensed under a Creative Commons Attribution 4.0 International License, which permits use, sharing, adaptation, distribution and reproduction in any medium or format, as long as you give appropriate credit to the original author(s) and the source, provide a link to the Creative Commons license, and indicate if changes were made.
In cases where reviewers are anonymous, credit should be given to 'Anonymous Referee' and the source.
The images or other third party material in this Peer Review File are included in the article's Creative Commons license, unless indicated otherwise in a credit line to the material. If material is not included in the article's Creative Commons license and your intended use is not permitted by statutory regulation or exceeds the permitted use, you will need to obtain permission directly from the copyright holder.
To view a copy of this license, visit https://creativecommons.org/licenses/by/4.0/

We thank both reviewers for their positive and constructive feedback. In response to their comments, we have substantially revised the manuscript to clarify the motivation for our analyses, moderated language where appropriate, and added several new analyses to support our findings and proposed mechanisms.

Most notably, we have added several extensive analyses, including:

- **An analysis of gene-level direction heterogeneity** and the effect of majority-vote thresholds on FSR and recall.
- **Simulations examining tissue tagging**, showing how non-relevant tissues can tag the GWAS signal.
- **Simulations assessing genetic architecture**, including the effects of LD, MAF, effect size, and eQTL sample size on both FSR and recall.
- **A reanalysis of the protein expression results using newer TWAS methods.**

Together, these additions further support our conclusion that high false sign rates are common across TWAS-related methods, and that these false sign rates often arise when non-relevant tissues tag the GWAS signal.

Reviewer #1 responses:

Gerlach and colleagues present a very compelling set of analyses that directs caution toward the direction of effect of the gene-disease association statistics generated by TWAS. The authors cleverly associate gene expression prediction models with protein measurements, as a valid hypothesis is that variants that increase gene expression should also increase protein expression. This study has some particularly impactful results such as

• Quantifying the proportion of gene-disease associations (23%-33%) with the incorrect direction of effect determined by TWAS. This leads to incorrect biological interpretations regarding if more or less of the gene increases or decreases disease risk.

• 85.3% of protein-encoding genes showed at least one TWAS-significant nearby gene, suggesting that non-causal tagging signals are highly prevalent.

• While purifying selection acting more strongly on trait-relevant tissues, stronger TWAS signals tend to be discovered in non-trait relevant tissues. This is an impactful statement because for many years, general advice regarding TWAS has been to exploit proxy tissues when needed as they will likely tag the causal SNP-trait effects and permit the recovery of the gene-disease association. However, this is not helpful if the sign is wrong.

The authors also propose coloc-based filtering and the usage of trait relevant tissue to remove the sign issue, which serves as a workable solution (if the sample size is large enough that coloc works well and if the trait relevant tissue is known which may not be the case), but ultimately limits the power to detect disease-critical genes as so few genes have high posterior probabilities of colocalization. Overall, I think this study was concise and makes a clearly compelling and cautionary point. However, there needs to be further analysis done in simulations to back up the claims of the authors regarding selection and co-regulation between tissues to quantitatively assess their impact on FSR. Also, there needs to be further investigation with state-of-the-art variations of TWAS to understand just how pervasive this FSR problem is.

We thank the reviewer for their positive comments on our paper.

Major Comments

1. The majority vote approach taken on lines 84-85 obscures the variability of the directions of effect. It would be interesting to quantify the gene-level variability of FSR across tissues where the gene is expressed. This might allow even stronger claims about FSR, as the analysis would reveal genes with high variability even in the scenario where the majority vote indicates concordant directions of effect.

We thank the reviewer for this suggestion. We agree that a simple majority-vote approach can obscure variability of the direction of effect across tissue categories. To address this, we quantified gene-level variability across tissue categories (bottom of panel A below). We found that 36.4% of genes had both positive and negative tissue-category directions, highlighting substantial within-gene heterogeneity in direction across tissue categories.

Furthermore, to test the effect of gene-level variability on FSR and recall, we varied the majority threshold used to assign an overall gene direction across tissue categories. Specifically, we compared the default 50% threshold with more stringent thresholds of 60%, 70%, 80%, 90%, and 100%, and then combined these direction assignments with colocalization filtering to evaluate gene-level FSR and recall (panel B below). We found that increasing the majority-vote threshold modestly decreased FSR while having little effect on recall.

We have revised the manuscript to include this analysis in Supplementary Note 1 and clarified the effects of stricter majority thresholds in the main text (page 5, lines 12-15):

> *"Requiring stronger directional consistency across tissue categories modestly reduced but did not eliminate high FSR, indicating that the observed FSR is not driven solely by ambiguous majority-vote assignments (Supplementary Note 1)."*

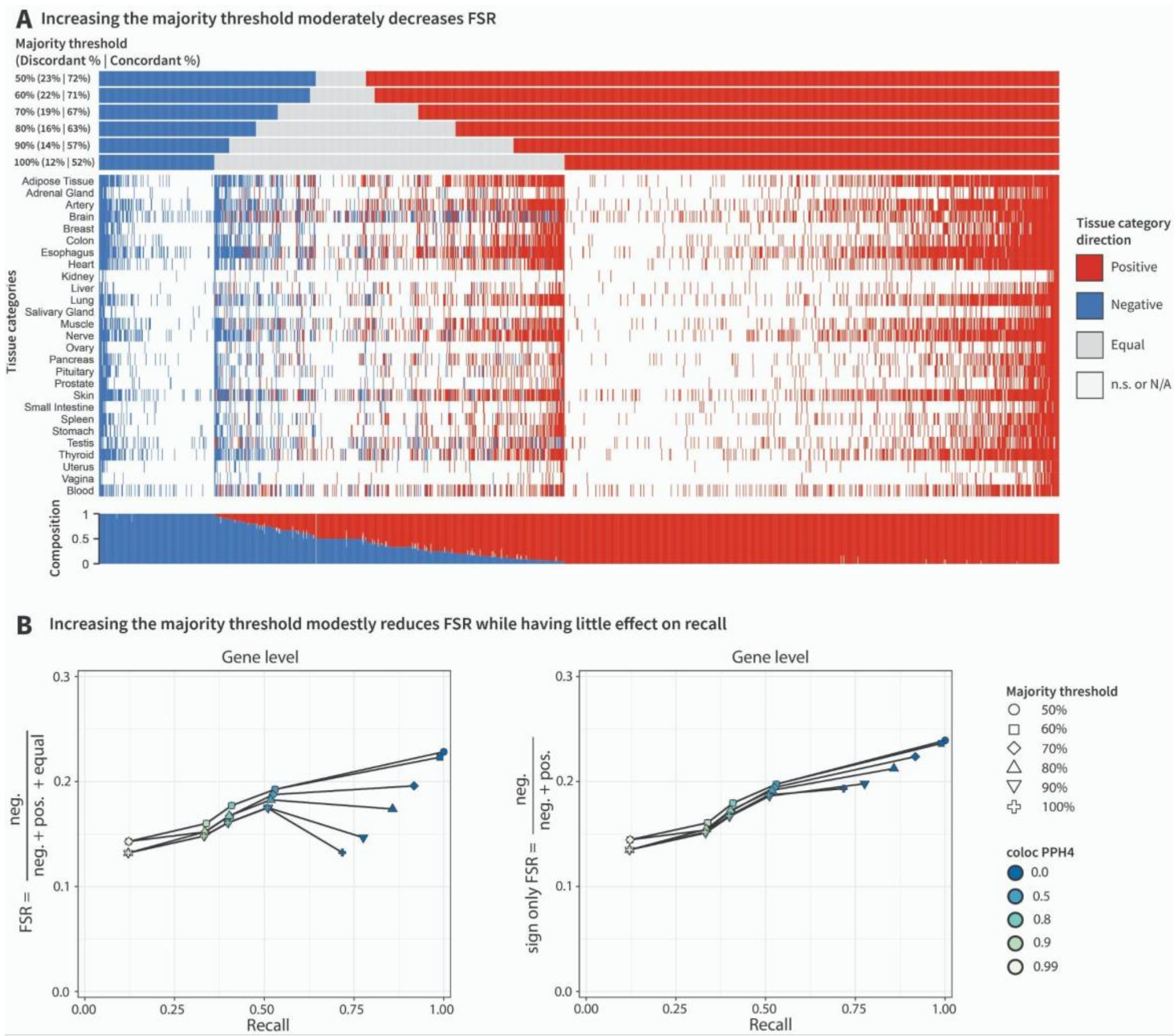

Supplementary Fig.1: Stricter majority-vote thresholds modestly decrease FSR while highlighting variability in direction across tissue categories. A, *Heatmap of TWAS direction-of-effect estimates for 1,543 plasma proteins (columns) across 27 tissue categories (rows), shown together with gene-level direction assignments under majority-vote thresholds of 50%, 60%, 70%, 80%, 90%, and 100%. Gene-level directions were assigned across tissue categories using the indicated majority-vote thresholds. The bottom composition bar shows, for each gene, the proportions of tissue categories with positive, negative, or equal directional evidence.* ***B,*** *FSR versus recall for gene-level directions after filtering by coloc PPH4 and assigning gene direction using majority-vote thresholds of 50%, 60%, 70%, 80%, 90%, or 100%. In the left panel, FSR was calculated as the proportion of negative genes among all retained genes, including neutral genes. In the right panel, FSR was calculated as the proportion of negative genes among retained signed genes only.*

2. While there were several hypotheses regarding what gene-level architectures give rise to FSR, the results would be more compelling if the authors could recapitulate those statements using simulations of gene and corresponding protein expression. Specifically,

having simulations of different causal variant architectures (i.e., shared causal variant(s) between gene and protein traits versus independent causal variant(s) or different causal variant(s) in LD) could add more support the coloc-based filtering strategy. The analysis on lines 158-168 partially gets at this question, but analysis of simulated genetic architectures would be more compelling than guessing and testing different gene-level characteristics in an empirical linear model.

We agree that simulations of distinct causal variant architectures are useful for evaluating when TWAS associations should or should not be retained by colocalization-based filtering. We note that these simulations were already included in the current Supplementary Note 2 to motivate the coloc and coloc-SuSiE filtering, but we recognize that this was unclear in the main text.

Specifically, we had run three scenarios similar to those suggested by the reviewer: a true-positive setting in which the causal GWAS and eQTL variants are the same, a false-positive setting in which the GWAS and eQTL causal variants are distinct but in LD, and a multi-signal setting in which one GWAS causal variant colocalizes with the eQTL while another independent GWAS variant has a larger effect. These simulations show that coloc-based filtering removes TWAS associations when the GWAS and eQTL signals are driven by distinct causal variants and are in moderate LD, while coloc-SuSiE can retain associations when one of multiple GWAS signals truly colocalizes with the eQTL.

We have revised the main text to more clearly connect the interpretation of each filtering step to the underlying causal variant architecture (page 7, lines 14-19):

> *"We show the expected behavior of these filters using simulations of three distinct causal architectures: (i) coloc-based filtering retains associations when the GWAS and eQTL signals share a causal variant; (ii) coloc-based filtering removes associations when the signals are driven by distinct causal variants in moderate LD; and (iii) coloc-SuSiE-based filtering can improve recall when the eQTL colocalizes with one of multiple GWAS signals (Supplementary Note 2)."*

3. Related to the comment above, simulations could help understand the FSR in the context of tagging tissues, which have a strong genetic correlation with the causal tissue, but do not harbor the specific variants or specific effect sizes that gave rise to the protein trait. In simulations, how does the degree of co-regulation between tissues (i.e., correlation of eQTL effect sizes) change the likelihood of FSR?

We agree with the reviewer that simulations would be useful for evaluating our proposed mechanism that non-trait-relevant tissues can produce false-sign TWAS associations when they tag the causal genetic signal from the relevant tissue.

To address this, we simulated GWAS summary statistics for 1,000 genes using blood as the causal tissue by assigning the top blood eQTL as the causal GWAS variant. We then ran TWAS across the rest of the tissues (see Panel A below). Using these estimates, we binned gene-tissue pairs based on the degree of co-regulation to the causal blood tissue (panel B below). As expected we found that the FSR was strongly dependent on co-regulation, with FSR decreasing when the correlation increased between genetically predicted expression in non-blood tissues and the causal blood tissue.

Notably, we found that colocalization filtering (panel B, right) not only enriched for gene-tissue category pairs that were strongly correlated with the causal blood tissue, but also enriched for gene-tissue category pairs that were strongly anti-correlated with the causal blood tissue. This pattern is consistent with our hypothesis that FSR is driven by scenarios where the GWAS and eQTL variants are distinct but in extreme LD, which neither TWAS nor colocalization can distinguish. As a result, non-blood eQTLs may have the opposite direction of effect from the blood eQTL, but still pass colocalization filtering because they tag the same GWAS-associated haplotype through very high LD.

We have now added this analysis as Supplementary Note 8 and revised the manuscript to discuss how the degree and direction of cross-tissue co-regulation influence FSR (page 14, lines 6-19):

> *"To explore whether this mechanism could contribute to FSR, we simulated GWAS summary statistics for 1,000 genes under a model in which blood was the known causal tissue (Figure S9A; Supplementary Note 8). We then ran TWAS across all tissues and evaluated the FSR of non-blood associations as a function of their co-regulation with blood (Figure S9B). As expected, FSR was highest for strongly anti-correlated tissues and decreased with increasing co-regulation with blood, tissues with anti-correlated genetic effects on expression were most likely to produce false-sign associations. Notably, after restricting to gene-tissue associations with coloc PPH4 > 0.5, we observed enrichment not only for tissues positively correlated with blood, but also for strongly anti-correlated tissues. This pattern is consistent with a model in which non-causal tissue eQTLs can pass colocalization filtering when they tag the same GWAS-associated haplotype through very high LD. In such cases, the non-blood eQTL may have the opposite direction of effect from the causal blood eQTL, while still appearing to colocalize with the GWAS signal because of high LD."*

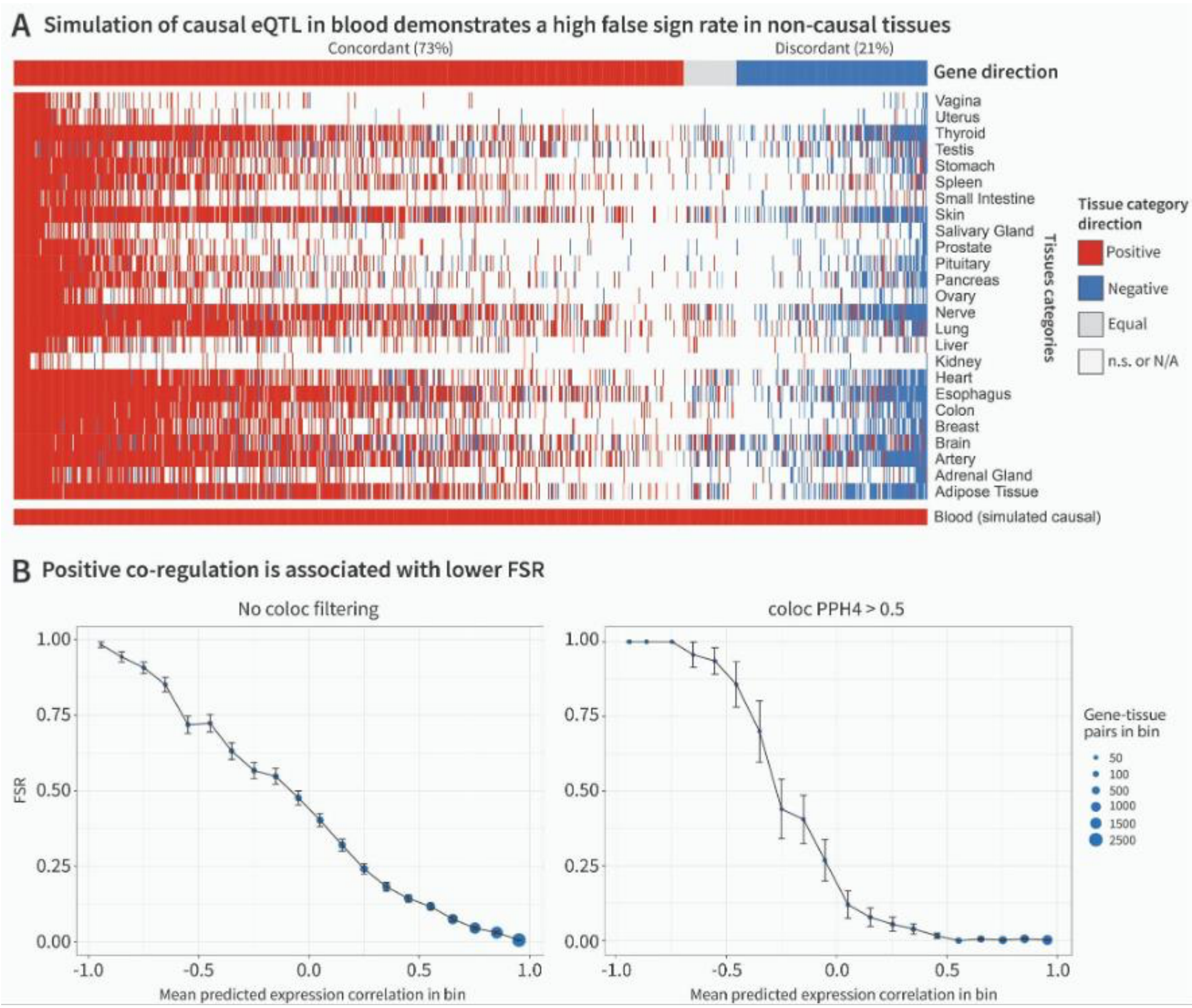

Supplementary Figure 9. Anti-correlated tagging tissues can produce false-positive evidence of colocalization. A. *Simulation framework used to evaluate FSR when Blood is the causal tissue. For each of 1,000 genes, we simulated GWAS summary statistics by setting the top Blood eQTL as the causal GWAS variant. The gene direction was calculated as previously from a majority vote across gene-tissue categories, but excluding blood.* ***B.*** *Gene-tissue pairs from non-Blood tissues were binned by the correlation between their genetically predicted expression and genetically predicted expression in Blood and FSR was calculated within each bin. The point size indicates the number of gene-tissue pairs within the bin. Error bars indicate ±1 standard error for the FSR estimate.*

4. The interpretation of stronger TWAS signals being discovered in non-trait relevant tissues because of purifying selection acting more strongly on trait-relevant tissues is a very impactful statement. Could the authors use simulations to show the degree of

selection that corresponds to a particular increase in FSR, for instance, using realistic estimates of selection across the transcriptome?

Unfortunately, we do not currently have realistic estimates of selection across the transcriptome to perform such a simulation. TWAS signals are driven by eQTL that are generally common, non-coding variants with tissue-specific effects on genes. In recent work, we have estimated the distribution of fitness effects for GWAS variants (Simons *et al.* 2025; Zhu *et al.* 2026), but these are not linked with causal genes or tissues. We and others have also estimated gene-level selection coefficients (Zeng *et al.* 2024), and while these are linked to genes, they are also not tissue-specific.

Our claim that purifying selection in trait-relevant tissues may make it more likely to detect TWAS signals in non-trait-relevant tissues is based on qualitative analysis we have conducted previously (Mostafavi *et al.* 2023). In Supplementary Figure 14 of Mostafavi *et al.* 2023 (copied in below), we review the model predictions for the discovery regions of eQTL and GWAS variants under selection. In this figure, the “eQTL” can be thought of as an eQTL in a non-trait-relevant tissue, while the “GWAS hit” can be thought of as an eQTL in a

trait-relevant tissue that results in the trait effect captured by GWAS.

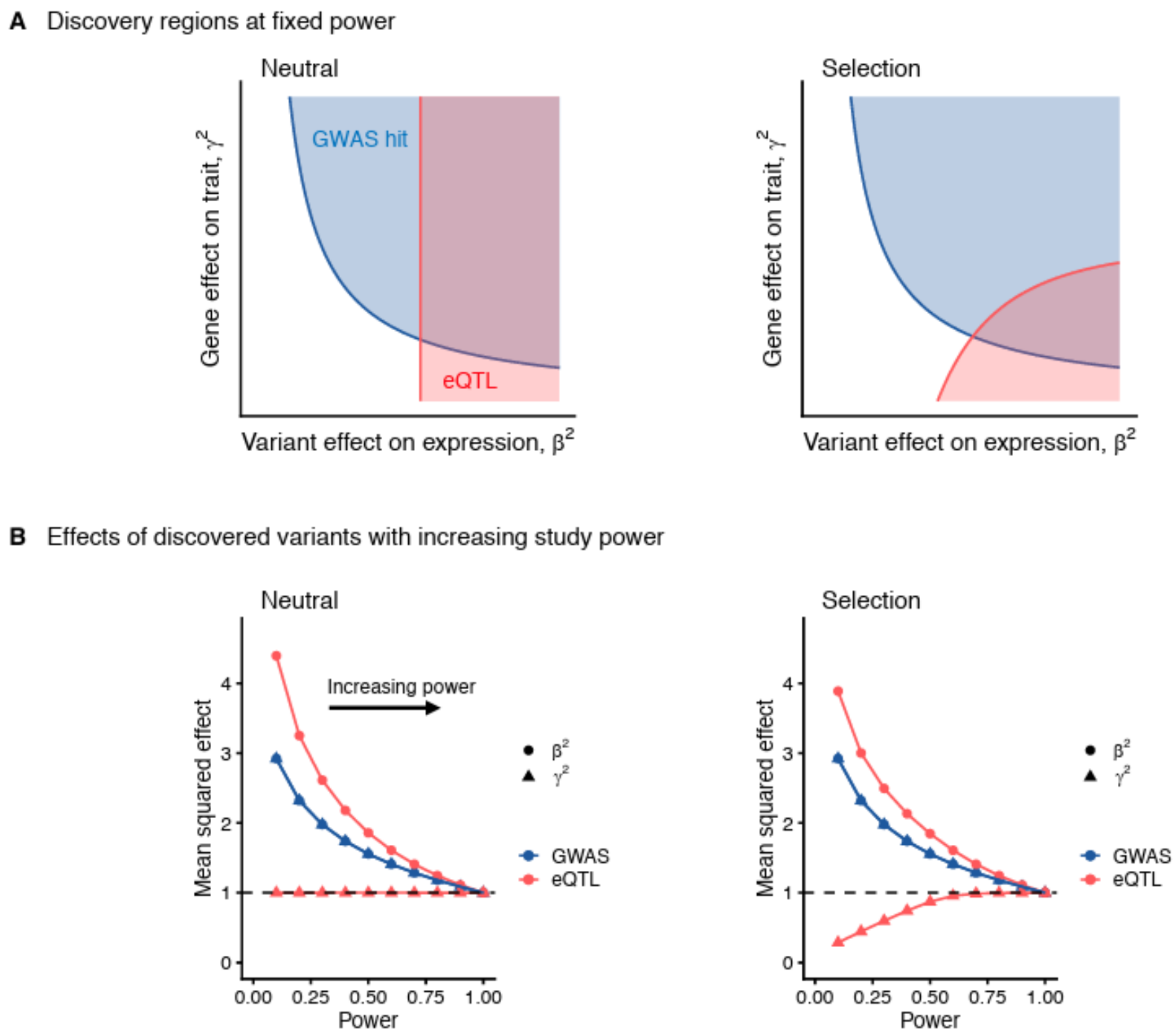

Supplementary Fig. 14: **Main qualitative model predictions.** *Model predictions under our baseline assumptions and choices of parameters: (i) a single cell type affecting a single phenotype, (ii) the SNP effect on gene expression,* β*, and the gene effect on phenotype,* γ*, drawn from independent standard Normal distributions, (iii) modeling selection to have a flattening effect formulated as* $E[2p(1-p)\beta^2\gamma^2|\beta,\gamma] \propto \kappa(1-e^{-\beta^2\gamma^2/\kappa})$*, where* p *is the allele frequency, and* κ *determines the strength of selection tuned to give* $\sim$*10% reduction in the average contribution to phenotypic variance across all causal SNPs relative to the neutral scenario. All results are based on 10 million simulated causal SNPs. See Supplementary Methods for details.* ***A)*** *Shading colors represent parameter space for the discovery of GWAS hit only (blue), eQTL only (red), and both types (purple) for the case of a neutrally evolving phenotype (left panel) and the case of a phenotype under selection (right panel). The discovery lines are derived assuming 15% power in both assays (see Online Methods).* ***B)*** *The mean properties of variants discovered as GWAS hits (blue) or eQTLs (red) with progressively increasing the discovery power in either assay. For a given study power* X*, points show the mean expression effect (mean* β^2 *values, circle) and the mean gene effect (mean* γ^2 *values, triangle) of the top* X*% of variants, ranked based on their strength of association signal in either assay, that is* $2p(1-p)\beta^2\gamma^2$ *for GWAS and* $2p(1-p)\beta^2$ *for eQTL mapping. The dashed lines show the mean values for all simulated causal SNPs.*

Under our model (which we test for various forms of misspecification in Supplementary Figures 15-20), the overlap of the discovery region for the two signals decreases when selection is present. We extended this model to a multi-context scenario, where each SNP

has an effect on multiple contexts, which have different effects on the trait (Supplementary Figure 23; copied in from [Mostafavi *et al.* 2023] below). This is the model that is most relevant to our intuition about the effect of selection on TWAS signals from trait-relevant and non-trait-relevant tissues.

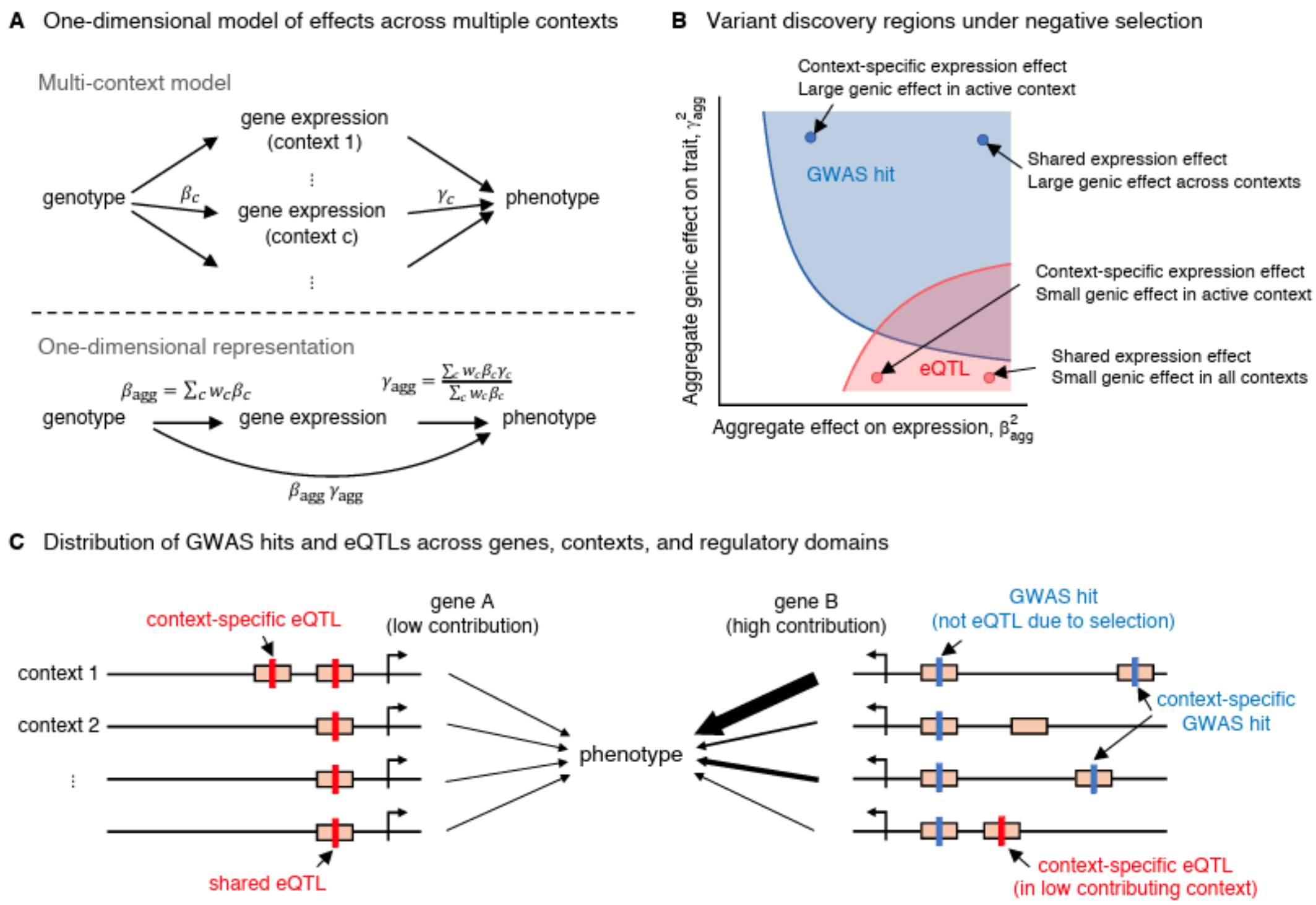

Supplementary Fig. 23: **A model for variant discovery across multiple contexts.** ***A)*** *One-dimensional representation of the pathway from genotype to phenotype mediated by gene expression across multiple contexts (e.g., cell types in a tissue), by defining parameters* β_{agg} *and* γ_{agg}. ***B)*** *Variant discovery in the space defined by* β_{agg} *and* γ_{agg} *for a phenotype under selection; same as in main Fig. 6B. Shading colors represent parameter space for the discovery of GWAS hit only (blue), eQTL only (red), and both types (purple).* ***C)*** *Schematic of variant discovery across multiple contexts mapped to regulatory domains (represented by orange rectangles) for two genes at different ends of the phenotypic importance axis.*

In this multi-context scenario, our model predicts that GWAS hits will be skewed towards genes with large effects on the trait, and towards trait-relevant tissue contexts. In contrast, eQTL in general will be skewed towards unimportant genes for traits. In addition, eQTL discovered at important genes will be skewed towards non-trait-relevant tissue contexts. Thus, we expect that eQTL in non-trait-relevant tissues will be easier to discover than the trait-relevant eQTL due to selection, which may contribute to false TWAS discoveries in non-trait-relevant tissues.

5. It would be good if the authors could provide guidelines to the reader such as how varying the sample size of the eQTL study and separately the GWAS study affect the FSR vs recall rates during the coloc-based filtering step, based on empirical data or simulated data. Under what scenarios does the coloc or coloc-susie filtering step perform worse than the results presented in Figure 2B? For example, how do the effect sizes of the causal variants, the allele frequencies, and the average LD between the causal variants affect the coloc-filtering results?

We agree that additional simulations are useful for understanding under which genetic architectures colocalization filtering is expected to reduce FSR, and when it may substantially reduce recall. To address this, we added two new simulation analyses in Supplementary Notes 3 and 4.

First, in Supplementary Note 3, we evaluated how different genetic architectures affected FSR, including effect size of causal variants, the minor allele frequency (MAF), the LD between the causal variants, and the eQTL study size of a tissue. We simulated GWAS summary statistics for a total of 1,500 genes across three tissues with different eQTL sample sizes (500 genes for each tissue). For all genes, we simulated the causal GWAS variant to be different from the causal eQTL, which was assumed to be the top eQTL from the top1 model in the GTEx expression models. For each gene, we varied the LD between the causal eQTL and GWAS variant, the MAF of the GWAS variant, and the effect size of the GWAS variant. We then ran FUSION TWAS using these simulated GWAS summary statistics together with the GTEx expression models (see figure below).

We found that after colocalization-filtering, LD between the causal GWAS variant and the causal eQTL was the only parameter that affected FSR. Specifically, we observe that variants in high LD have increased FSR compared to when the causal variants are in modest to low LD, even after colocalization filtering (Panel B below). Notably, this supports our hypothesis that FSR is driven by eQTLs in non-causal tissues tagging the causal GWAS variant through very high LD.

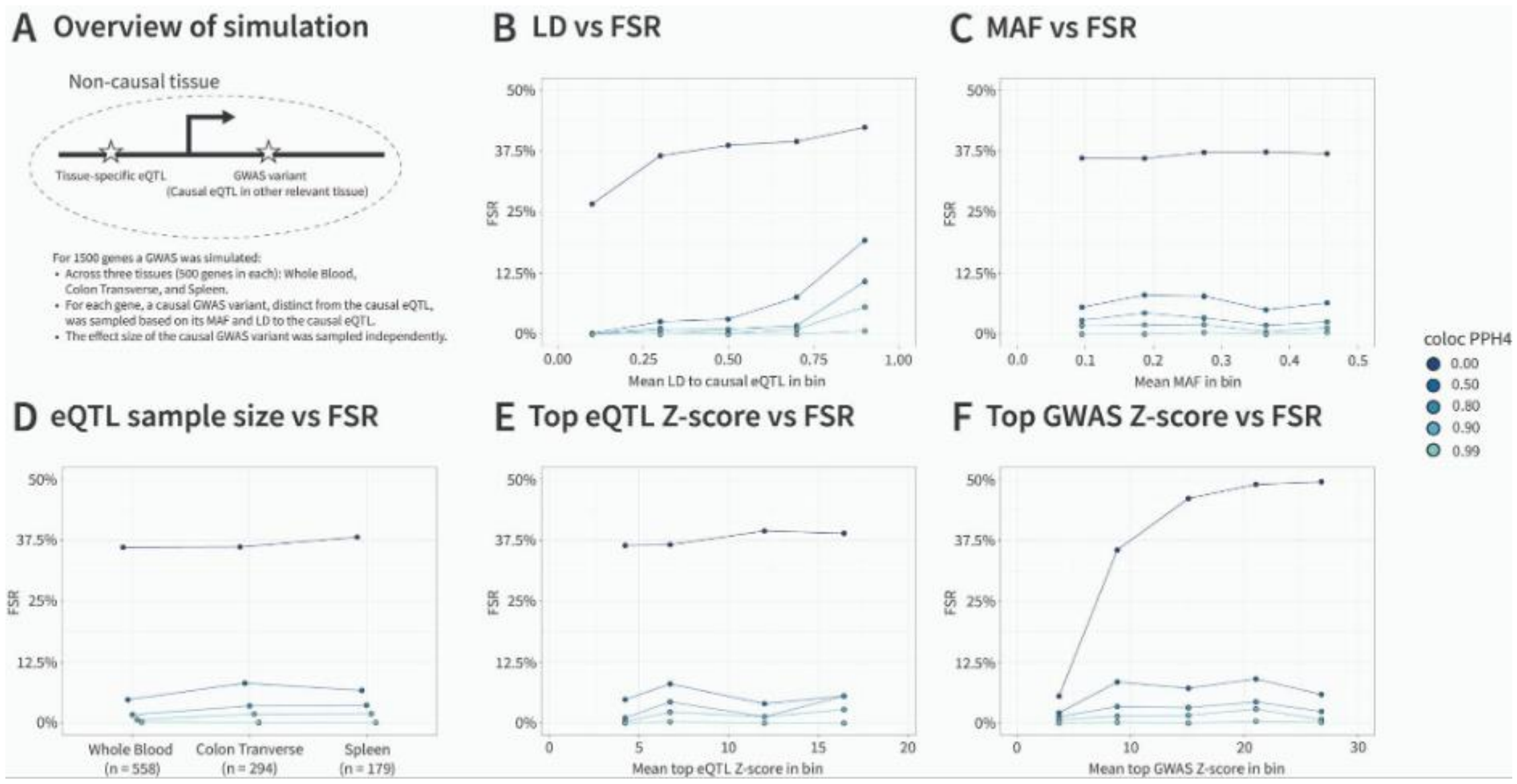

Supplementary Figure 3. The effect of genetic architecture on FSR and colocalization filtering. A. *Simulation framework. For each gene, a causal GWAS variant was sampled to be distinct from the causal eQTL, with varying LD to the causal eQTL, MAF, and effect size. Simulations were performed across Whole Blood, Colon Transverse, and Spleen.* ***B–F.*** *FSR was estimated after binning gene–tissue pairs by mean LD to the causal eQTL (**B**), MAF (**C**), eQTL sample size (**D**), top eQTL Z-score (**E**), and top GWAS Z-score (**F**). Colors indicate coloc PPH4 filtering thresholds.*

Next, in Supplementary Note 4, we evaluated how genetic architecture affected recall after colocalization filtering. For each gene, we simulated two causal GWAS variants: one that is the top eQTL in the focal tissue, and one distinct, non-shared variant that acts as a confounder from a non-focal tissue. As in the previous simulation, we varied the LD between the two causal variants and the MAF of the non-shared variant. We applied this framework to the same 1,500 genes across three tissues. Unlike the previous simulation, however, each gene was simulated under three relative effect-size scenarios: equal-strength GWAS effects for the shared and non-shared variants, a stronger shared eQTL/GWAS signal, and a stronger non-shared GWAS signal.

This simulation showed that stringent colocalization filtering can substantially reduce recall, even when a true shared eQTL/GWAS signal is present. In particular, filtering at coloc PPH4 ≥ 0.99 reduced recall to below 10% in most scenarios, except when the eQTL signal was strong in the equal-strength or shared eQTL/GWAS-variant-stronger scenarios

(Panel E below). This indicates that true-positive TWAS associations can be removed by stringent colocalization thresholds, especially when the shared eQTL/GWAS signal is modest.

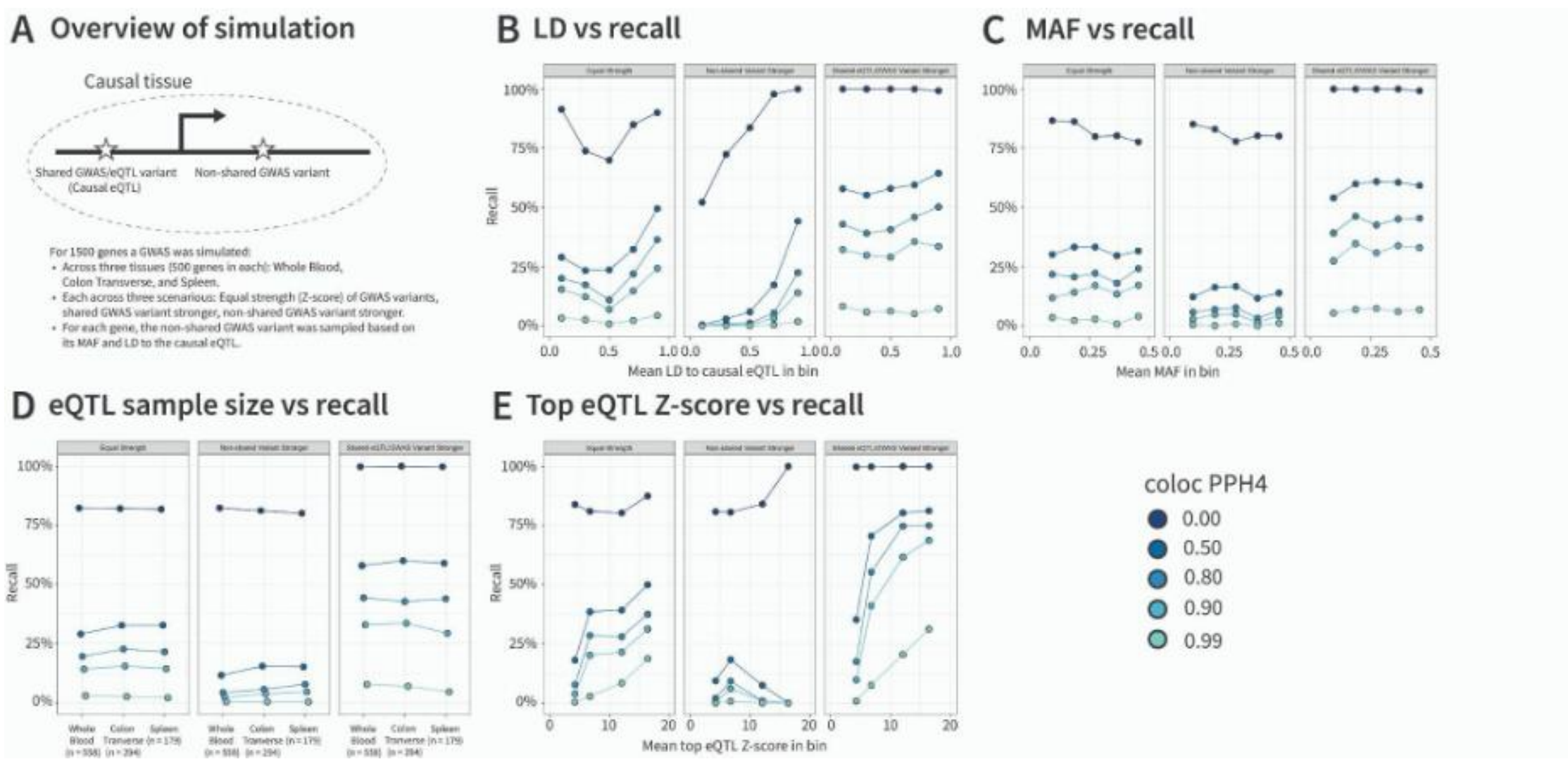

***Supplementary Figure 4. The effect of genetic architecture on recall and colocalization filtering. A.** Simulation framework. For each gene, we simulated two causal GWAS variants: one variant shared with the causal eQTL, assumed to be the top eQTL, and one non-shared GWAS variant sampled to vary in LD with the causal eQTL, MAF, and effect size. Simulations were performed across Whole Blood, Colon Transverse, and Spleen under three effect-size scenarios: equal strength of the shared and non-shared GWAS variants, stronger signal of the non-shared GWAS variant, and stronger signal of the shared eQTL/GWAS variant. **B–E.** Recall was estimated after binning gene–tissue pairs by mean LD between the non-shared GWAS variant and the causal eQTL (**B**), MAF of the non-shared GWAS variant (**C**), eQTL sample size (**D**), and top eQTL Z-score (**E**). Columns show the three strength scenarios. Colors indicate coloc PPH4 filtering thresholds.*

Together, these simulations provide several useful guidelines. They highlight that the FSR is primarily driven by non-relevant-tissues tagging the causal GWAS variant, which even colocalization filtering cannot resolve. At the same time, colocalization filtering leads to substantial loss of recall, even when a true colocalizing signal is present but modest. We have added these analyses as Supplementary Notes 3 and 4 and Supplementary Figures 3

and 4, and revised the manuscript to discuss how genetic architectures affect FSR and recall after colocalization filtering (page 7, lines 35-41):

> *"To further characterize how different genetic architectures affected FSR and recall after colocalization filtering, we performed different simulations with varying minor allele frequency (MAF), effect sizes, eQTL sample sizes, and LD between causal variants (Supplementary Notes 3-4). We found that high LD between the causal GWAS and causal eQTL was the strongest contributor to high FSR after colocalization filtering (Figure S3). Meanwhile, we found that stringent colocalization filtering also substantially reduced recall in scenarios with true shared GWAS/eQTL causal variant (Figure S4)."*

6. Do TWAS frameworks that model uncertainty of predicted expression have decreased FSR? Or is the issue more about the (un)stated assumptions of TWAS? In a tagging tissue at infinite sample size, there is perfect certainty regarding the eQTL effects but because they only tag the causal effects in the correct tissue, FSR will still occur. At finite sample size, will models that consider the uncertainty of predicted expression improve the FSR?

We believe that the inflated FSR arises primarily from tight LD between eQTL that cannot be faithfully distinguished by TWAS. From the Mendelian randomization (MR) perspective, this is a violation of exchangeability (Sanderson *et al.* 2022, Figure 6A) rather than an issue of error in predicted expression. Therefore, we expect that TWAS frameworks that model uncertainty of predicted expression will not have a decreased FSR.

In general, measurement error biases the TWAS estimate towards zero and reduces the power of the test with a null hypothesis of no effect (Fuller 1987, Section 1.1.1). However, as long as the measurement error is independent of the trait, which is a reasonable assumption (Liang *et al.* 2026), it does not increase type 1 errors. Thus, the measurement error should reduce power, but should not bias the sign of the estimate. Even if we model the uncertainty in expression, it would not abrogate the confounding from linkage. Thus, we would expect the FSR problem to persist even for models that consider uncertainty in expression.

7. Do recent TWAS methods that analyze association statistics across tissues improve FSR? There have been some recent methods published that the authors should compare their FSR calculations to:

- Feng et al 2021 PLoS Genetics https://pubmed.ncbi.nlm.nih.gov/33831007/

• Barbeira et al 2019 PLoS Genetics https://pubmed.ncbi.nlm.nih.gov/30668570/

• Hu et al 2019 Nature Genetics https://pmc.ncbi.nlm.nih.gov/articles/PMC6788740/

We thank the reviewer for the suggestion and agree that recent multi-tissue TWAS methods provide an important comparison point. However, one challenge is that these TWAS aggregation methods are designed primarily to improve power and do not provide a single interpretable direction of effect for the combined gene-level test. All of the methods mentioned above (sCCA+ACAT, S-MultiXcan, and UTMOST) provide a p-value, but not a direction-of-effect estimate for genes across tissues.

For this reason, we instead repeated the protein expression analysis using multigroup causal TWAS (M-cTWAS; Qian et al. 2026), which can jointly analyze multiple expression models. As M-cTWAS produces gene PIPs for each tissue included in the model, we assigned the direction for each gene using the effect estimate in the tissue with the highest PIP. We found that M-cTWAS showed similar levels of FSR and recall compared to FUSION and cTWAS (see panel C in the figure for the comment below), suggesting that high FSR persists even when tissue-specific expression models are analyzed jointly. We have added this analysis to Supplementary Note 5 in the manuscript and explained the results in the main text (page 31, lines 8-17) together with the cTWAS analysis (see comment below).

8. Do methods that integrate TWAS with causal inference such as causal-TWAS (cTWAS, Zhao et al 2024, Nature Technical Reports) reduce the FSR?

We thank the reviewer for this suggestion. We have now repeated the protein-expression analysis using causal TWAS (cTWAS). We evaluated both unfiltered cTWAS gene Z-scores (panel A below) and PIP-filtered cTWAS associations (panel B below). We found that unfiltered cTWAS estimates resulted in similar levels of discordance to FUSION estimates (24% for cTWAS and 23% for FUSION). Filtering gene-tissue associations by cTWAS PIP moderately reduced FSR, but this reduction was accompanied by a substantial loss of recall, similar to when we filtered FUSION TWAS by coloc PPH4. Thus, our results suggest that the high FSR observed in the protein expression analysis is not specific to FUSION and persists even when using more recent TWAS methods that account for local genetic confounding. We have added these analyses to Supplementary Note 5 and explained the results in the main text (page 8, lines 1-8) together with M-cTWAS:

> *"Recent TWAS methods model (i) local genetic confounding and (ii) multiple tissues simultaneously. We first assessed whether more recent TWAS approaches that explicitly model local genetic confounding could further reduce FSR*

(Supplementary Note 5). To test this, we repeated the protein-expression analysis using cTWAS35, which jointly models molecular traits and nearby genetic variants within a locus. Applying the same tissue-category and gene-level majority-vote strategy as for FUSION, unfiltered cTWAS gene Z-scores showed similar discordance to FUSION: 24% (95% CI: 22-27%) vs. 23% (95% CI: 20-25%) for cTWAS and FUSION, respectively (Figure S5A). While filtering associations by cTWAS PIP modestly reduced FSR, it was accompanied by a substantial loss of recall (Figure S5B). Next, to assess whether multi-tissue modeling improved FSR, we applied multigroup cTWAS (M-cTWAS)36, an extension of cTWAS designed to jointly analyze multiple QTL datasets. Because M-cTWAS produces tissue-specific gene PIPs rather than a single gene-level direction, we assigned each gene's direction using the effect estimate in the tissue where that gene had the highest PIP. M-cTWAS showed similar FSR and recall to single-tissue cTWAS and FUSION (Figure S5C), indicating that integrating tissue-specific expression models did not substantially reduce the high FSR observed in the protein-expression analysis. These results suggest that the high FSR is not specific to FUSION and persists with newer TWAS approaches."

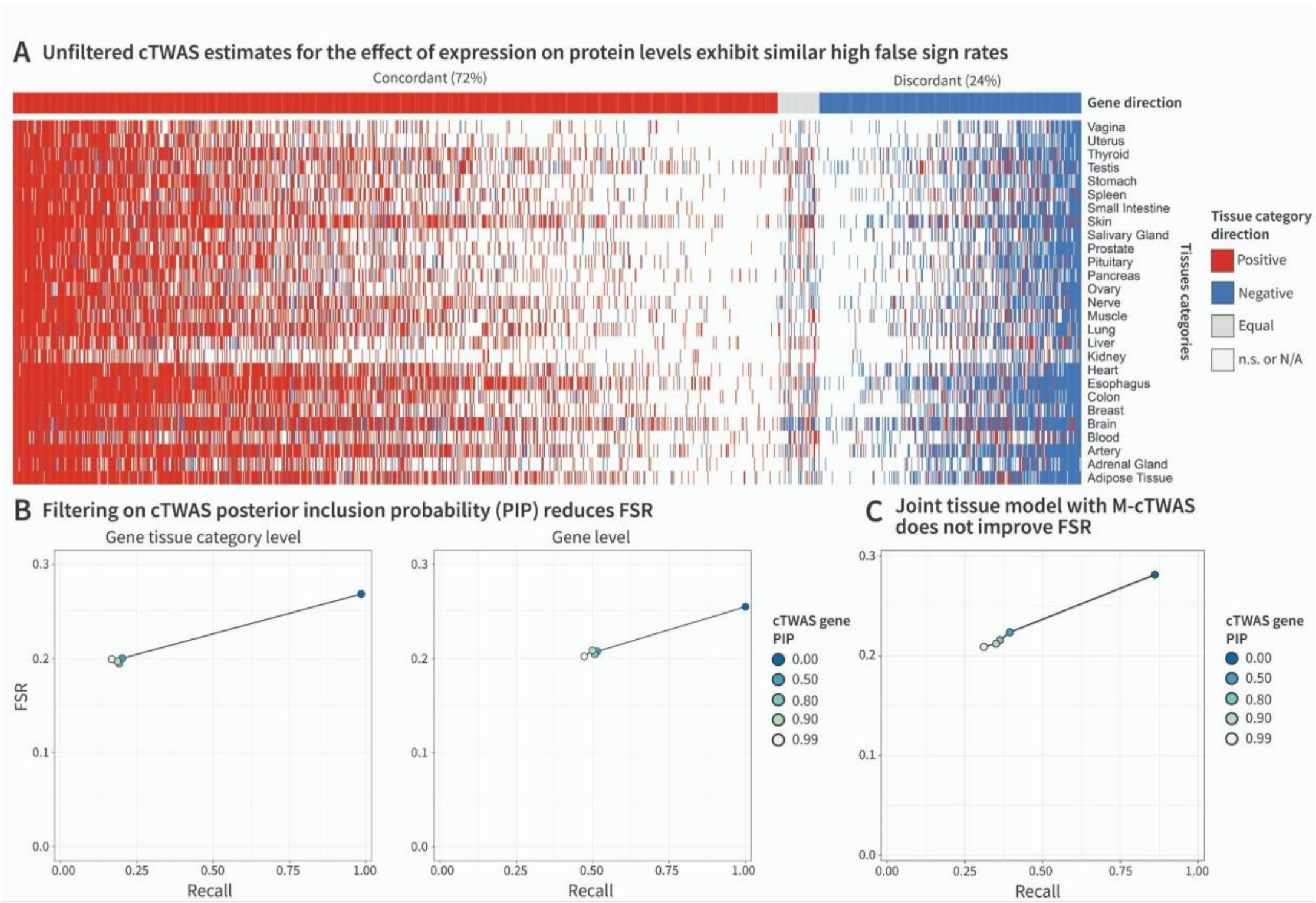

Supplementary Figure 5. cTWAS and M-cTWAS show similar high levels of false sign rates. A. *Heatmap of direction-of-effect estimates from unfiltered cTWAS gene Z-scores for protein expression associations. Columns represent protein-coding genes and rows represent tissue categories. For each gene-tissue category, directions were assigned by majority vote across tissues within the category, with positive effects shown in red, negative effects in blue, equal evidence in gray, and non-significant or unavailable estimates in white. The top annotation shows the final gene-level direction assigned by majority vote (50%) across tissue categories.* ***B.*** *FSR versus recall for cTWAS after filtering gene-tissue associations by cTWAS posterior inclusion probability (PIP). Results are shown at the gene-tissue category level (left) and gene level (right) across PIP thresholds of 0, 0.5, 0.8, 0.9, and 0.99.* ***C.*** *FSR versus recall for M-cTWAS. For each gene, direction was assigned using the tissue-specific effect estimate with the highest PIP, and results are shown across the same PIP thresholds as for* ***B****.*

Minor Comments

1. In the early paragraphs of the manuscript, it was not immediately clear that protein levels were being used as the complex traits in the GWAS and that gene expression levels were being used to build the predicted expression models. At first, it seemed like protein

expression models were being built and then associated with GWAS sumstats for complex traits/diseases (i.e., a PWAS). The authors should clarify earlier in the introduction main text that GTEx data (with a report of the number of tissues and genes/tissue) is being used to construct the genetic prediction models. It is mentioned in Figure 1 and in the caption, but it should appear in the main text as well, probably near line 75 when the UKB-PPP was first introduced. Related to this, line 68 was confusing because "TWAS predictions of plasma protein" imply that you are predicting protein levels, but rather what was meant was "TWAS associations of plasma protein."

Thanks for the suggestion. We have clarified that plasma protein abundance was used as the GWAS phenotype, while genetically predicted gene expression was estimated using GTEx-trained expression prediction models across 47 tissues, and revised the wording from "TWAS predictions of plasma protein" to "TWAS associations between the expression of a gene and its protein abundance" .

> *"To evaluate the FSR of TWAS direction-of-effect estimates, we used plasma protein expression as a positive-control GWAS phenotype, since higher genetically predicted expression of a gene is generally expected to increase the abundance of its encoded protein."*
>
> *"As a result, imperfect RNA–protein correlation would be expected to reduce power to detect TWAS associations between the expression of a gene and its protein abundance, but not reverse the estimated direction-of-effect."*
>
> *"To quantify the FSR of TWAS direction-of-effect estimates for plasma proteins, we applied the FUSION TWAS framework with expression prediction models trained in GTEx across 47 tissues. We applied these models to the same 2,923 plasma proteins from the UKB-PPP, to test whether genetically predicted expression of each gene was associated with the protein expression of its encoded plasma protein."*

2. In the discussion of Figure 2B, I believe attributing fault to TWAS entirely for the FSR is an overstatement. As demonstrated by Figure 2B, simple visualization of the eQTL and GWAS sumstats is sufficient to see that the directions of effect are opposing, and this can be unexpected. In other words, it is not the job of TWAS to correct this direction of effect issue, but rather to report the magnitude of the cis-genetic correlation. So I think the authors need to qualify the strength of their language in reporting that this is a shortcoming of TWAS. One could argue this is a shortcoming of eQTL associations as well, since they capture the effects of tagging tissues.

We agree that our findings should not be interpreted as evidence that TWAS itself introduces discordant associations, but rather it can arise from the underlying eQTL data used by TWAS and similar methods. However, the high FSR remains a practical limitation when TWAS signs are interpreted as biological direction-of-effect estimates. We have now clarified this distinction in the Discussion section (page 15, lines 23-27):

> *"We note that the observed discordance should not be interpreted as evidence that TWAS itself introduces erroneous signs, but rather that the discordance can arise from the underlying eQTL data used by TWAS and other eQTL-based methods. Nevertheless, it remains a practical limitation when TWAS signs are interpreted as biological direction-of-effect estimates."*

3. Could the authors quantify their statement in line 152: "TWAS was strikingly confident in the incorrect association direction in multiple tissues." Does this imply that the magnitude of the TWAS association statistic and/or the p-values were particularly significant in these discordant directions of effect?

Thank you for highlighting this imprecise statement. Here, we meant for readers to note that the proportion of tissues with significant TWAS associations in the incorrect direction was striking. We have now rephrased this statement and quantified it as follows:

> *"Strikingly, for 12% of the total gene-trait pairs (Figure 3A, far right of the heatmap), TWAS was confident in the incorrect direction in greater than 90% of tissues."*

Reviewer #2 (Remarks to the Author):

Gerlach et al. benchmark the performance of transcriptome-wide association studies (TWAS) at predicting the direction of effect of causal genes at GWAS risk loci on the GWAS trait. They use two benchmarks where both the causal gene and its direction of effect are known with fairly high confidence, one based on proteomics GWAS and the other based on exome burden-test hits. This paper is important because it is (to my knowledge) the first paper to benchmark eQTL-based methods at direction-of-effect prediction from GWAS. Direction-of-effect prediction is something that eQTL-based methods like TWAS should in theory be stronger at than causal gene identification (where co-regulation is an inherent limitation), and is highly relevant to pharmaceutical development. A secondary reason the paper is important is because it provides a new orthogonal benchmark of the performance of eQTL-based methods more generally, since as the paper notes most prior benchmarking has focused on causal gene prioritization, not direction-of-effect prediction.

We thank the reviewer for their positive comments on our paper.

Comments:

1. Though RNA and protein expresion are correlated, the correlation is far from perfect, and there is also discordance between eQTLs and pQTLs, as shown in a paper co-authored by Dr. Pritchard (Battle et al., "Impact of regulatory variation from RNA to protein", Science 2014). The analysis described in the first paragraph of the results tries to allay this concern, but does not (see point #2). This matters because if there's discordance between RNA and protein expression, the false sign rate for TWAS in the UKB-PPP analysis may look worse than it actually is, since TWAS may have the correct sign for RNA expression, but a flipped sign for protein expression, merely because of this RNA-protein expression discordance. Fortunately, this concern does not apply to the complex-trait analysis, so the headline result of the paper (that TWAS has a high false sign rate) still stands. Even so, the authors should either conduct a sensitivity analysis to address this (though it's not clear what such an analysis would look like), or mention and rebut this concern in the Discussion. (The rebuttal being that the complex-trait analysis shows the same general result.)

We thank the reviewer for the clarification. While we agree that RNA and protein expression are imperfectly correlated as shown in Battle et al., this is due to attenuation of eQTL effects at the protein expression level rather than due to reversal of effect direction. Thus, the imperfect correlation of RNA and protein expression likely reduces the number of significant TWAS associations, but it would not be expected to generate highly significant discordant direction-of-effect estimates as we observe in this study. We have clarified this distinction in the start of the result section (page 4, lines 15-21):

> *"While RNA and protein expression is imperfectly correlated, this reflects attenuation or buffering of RNA-level regulatory effects at the protein level, rather than reversal of their direction. As a result, imperfect RNA-protein correlation would be expected to reduce power to detect TWAS associations between the expression of a gene and its protein abundance, but not reverse the estimated direction-of-effect. We therefore expect genetically predicted expression of the encoding gene to be positively associated with its protein expression (Figure 1D)."*

2. The importance of the analysis in the first paragraph of the results is not clear. It does not "support the expectation that gene expression is generally positively associated with

protein expression" since it is just showing that LoF variants are associated with decreased protein expression and duplications are associated with increased protein expression; RNA expression is not part of this analysis. Thus, statements like "we expect all confident TWAS predictions of plasma protein to be positive" and "these results support the expectation that gene expression is generally positively associated with protein expression" seem out of place here. It's also not clear that this result "makes plasma protein levels an attractive phenotype to assess the false sign rate (FSR)", as the caption to Figure 1E mentions, since it would be an attractive benchmark even without this analysis.

We thank the reviewer for this suggestion. We agree that the LoF and duplication burden analyses do not directly measure RNA expression. However, LoF variants and duplications are well-motivated genetic perturbations that affect gene expression: LoF variants decrease functional gene dosage and thereby reduce effective gene expression, whereas duplications increase gene dosage and thereby increase expression (*Chiang et al. 2017*; *Milind et al. 2026*). Thus, although RNA expression is not directly measured in this analysis, the observation that LoF burden decreases plasma protein abundance while duplication burden increases plasma protein abundance supports the expected positive direction between gene and protein expression. The purpose of this analysis was therefore to establish the expected direction for TWAS associations. Because genetically predicted expression captures subtler variation in dosage of the encoding gene, confident TWAS associations with abundance of the encoded protein should generally be positive, consistent with the direction implied by the LoF and duplication burden tests.

We have now revised the relevant paragraph in the result section (page 4, lines 24-29):

> *"Using protein expression measurements of 2,923 plasma proteins from the UK Biobank Pharma Proteomics Project (UKB-PPP), we tested whether LoF variants or duplications in the encoding gene were associated with altered protein expression of that same protein. Because LoF variants and duplications are expected to decrease and increase gene dosage, and consequently effective gene expression, they are expected to decrease and increase protein expression of the encoded protein, respectively."*

3. The first paragraph of the results would be easier to follow if it's explicitly explained that a) this is using the UKB-PPP, b) the analysis is looking at associations between the level of each protein and LoF variants or duplications *in/of the same protein*. This would require some restructuring since the UKB-PPP currently isn't introduced until the following paragraph.

We have now specified that a) the LoF and duplication burden tests used UKB-PPP and b) clarified that the analysis associates LoF variants or duplications in each encoding gene and the abundance of the corresponding encoded protein (page 4, lines 24-26):

> *"Using protein expression measurements of 2,923 plasma proteins from the UK Biobank Pharma Proteomics Project (UKB-PPP), we tested whether LoF variants or duplications in the encoding gene were associated with altered protein expression of that same protein."*

4. TWAS associations may not have the same direction of effect across tissues. To account for this, the authors take a majority vote within each tissue, and then another majority vote across tissues. The paper would benefit from some exploration of how often direction of effects disagree within and across tissues. It would also be worthwhile to look at the false sign rate when subsetting to genes where the TWAS results are consistent across tissues, or at least mostly consistent. For instance, they could subset to genes with significant TWAS associations in at least N distinct tissue categories, and where at least M <= N of these associations are in the same direction - but there are many possible formulations. This would likely remove fewer genes than colocalization-based filtering, and would avoid the problem of selecting a single "correct" tissue: as the authors note, "identifying the relevant tissues for complex traits remains an open challenge".

We agree with the reviewer that gene-level variability in directional estimates across tissue categories warrants additional exploration. To address this, we quantified gene-level variability of directional estimates across tissue categories (bottom of panel A below). We found that 36.4% of genes had both positive and negative tissue-category directions, highlighting substantial within-gene heterogeneity in direction across tissue categories.

As suggested by the reviewer, we next examined whether greater directional consistency across tissue categories was associated with lower FSR. To test this, we increased the majority-vote threshold required to assign an overall gene direction across tissue categories. Specifically, we compared the 50% threshold used in the main analysis with thresholds of 60%, 70%, 80%, 90%, and 100%, and combined these alternative direction assignments with colocalization filtering to evaluate gene-level FSR and recall (Supplementary Figure 1B). We first calculated FSR and recall using all retained genes in the denominator (as done in prior analyses), including genes classified as neutral (Supplementary Figure 1B, left). Because a substantial fraction of genes become neutral at higher majority thresholds, this formulation can be somewhat misleading, making stricter majority thresholds appear to achieve FSR values comparable to very stringent colocalization filtering while retaining much higher recall. We therefore also calculated

FSR using only positively and negatively classified genes in the denominator (Supplementary Figure 1B, right). Under this alternative formulation, increasing the majority-vote threshold modestly decreased FSR while having little effect on recall.

We revised the manuscript to include this analysis in Supplementary Note 1 and have clarified the effects of stricter majority thresholds in the main text (page 5, lines 13-16):

> *"Requiring stronger directional consistency across tissue categories modestly reduced but did not eliminate high FSR, indicating that the observed FSR is not driven solely by ambiguous majority-vote assignments (Supplementary Note 1)."*

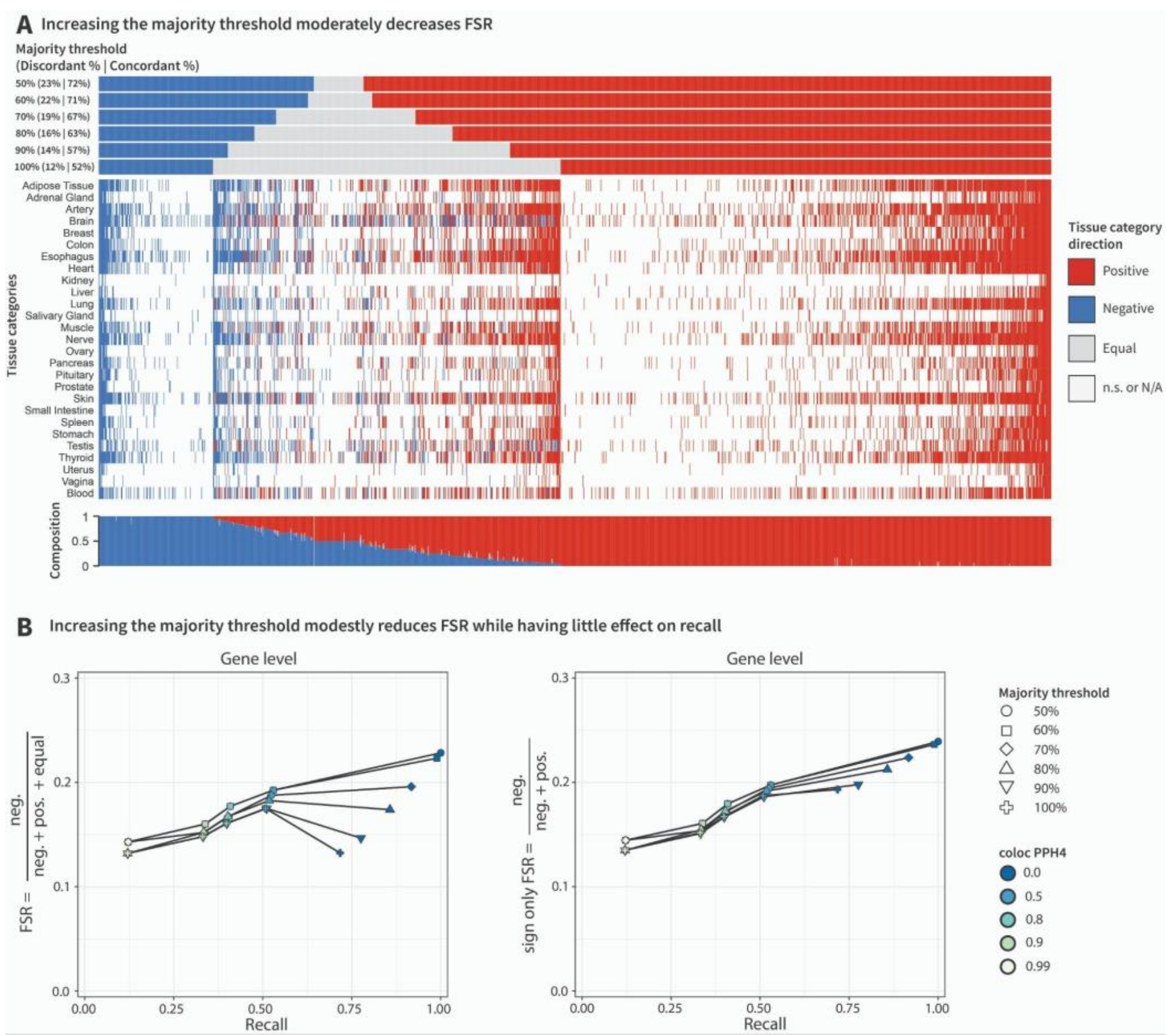

Supplementary Fig.1: Stricter majority-vote thresholds modestly decrease FSR while highlighting variability in direction across tissue categories. A, *Heatmap of TWAS direction-of-effect estimates for 1,543 plasma proteins (columns) across 27 tissue categories (rows), shown together with gene-level direction assignments under majority-vote thresholds of 50%, 60%, 70%, 80%, 90%, and 100%. Gene-level directions were assigned across tissue categories using the indicated majority-vote thresholds. The bottom composition bar shows, for each gene, the proportions of tissue categories with positive, negative, or equal directional evidence.* ***B,*** *FSR versus recall for gene-level directions after*

filtering by coloc PP.H4 and assigning gene direction using majority-vote thresholds of 50%, 60%, 70%, 80%, 90%, or 100%. In the left panel, FSR was calculated as the proportion of negative genes among all retained genes, including neutral genes. In the right panel, FSR was calculated as the proportion of negative genes among retained signed genes only.

5. The abstract mentions that subsetting on coloc + trait-relevant tissues reduces the false sign rate to 0% - but this is on just 40 genes. It's also not consistent when using coloc-SuSiE instead of coloc, where the false sign rate is 7%. It doesn't seem like a practical solution and doesn't feel like it belongs in the abstract. What seems much more notable - and honestly an abstract-level conclusion - is that the false sign rate in the UKB-PPP analysis was 20% *even after subsetting to trait-relevant tissues*, nearly the same as the 23% false sign rate when using all tissues. And this is in the ideal scenario where the correct tissue is known; that 3% gap would easily disappear for complex traits where there is ambiguity about the correct tissue. In other words, filtering to trait-relevant tissues has essentially no detectable effect on the ability of TWAS to predict the correct direction of effect. This seems greatly underemphasized in the paper relative to how important it is.

We agree with the reviewer that subsetting to trait-relevant tissues is not a practical solution and have revised the abstract to remove the sentence emphasizing the 0% FSR after combined trait-relevant tissue and colocalization filtering. We also agree that the limited effect of trait-relevant tissue restriction alone is an important result. We have revised the results to more clearly state that restricting to trait-relevant tissues alone only modestly reduced the FSR in the UKB-PPP analysis, from 23% across all tissues to 20%, despite this being an unusually favorable setting in which the relevant tissue can often be defined more clearly than for complex traits (page 10, lines 36-40).

> *"Overall, restricting to the trait-relevant tissue only modestly reduced discordance, with 20% (95% CI: 15-26%) of proteins discordant using this approach (Figure 4A), compared to 23% (95% CI: 20-25%) when using a majority vote strategy across tissues (Figure 2A). However, discordance was particularly high for brain-specific proteins and excluding these associations reduced the FSR to 14% (95% CI: 9-20%)."*

We have also clarified that the purpose of the combined trait-relevant tissue and colocalization analysis is not to present a broadly practical filtering strategy, but to show that these two filters provide complementary evidence. Trait relevance supports the biological context of the eQTL, while colocalization supports a shared genetic signal between the eQTL and GWAS association (page 11, lines 5-8):

"Together, these results suggest that combining trait-relevant tissue information with colocalization-based filtering provide complementary evidence to improve the directional accuracy of TWAS effect estimates: tissue relevance supports the biological context of the association, while colocalization supports a shared genetic signal between the eQTL and GWAS. However, we recognize that identifying the relevant tissues for complex traits remains an open challenge23,31,40."

6. Related to point #5, it would be nice to know whether the 3% gap is a significant difference. It would be useful to calculate 95% confidence intervals for all of the false sign rates in the paper. These confidence intervals would be particularly large for the 40-gene and 58-gene groups - probably the 0% from coloc is not significantly different from the 7% from coloc-SuSiE.

We now report confidence intervals for all FSR estimates in the paper. As the reviewer suggests, the confidence intervals indicate that the overall FSR after assigning directions using trait-relevant tissues was not significantly different from the FSR obtained using the majority-vote strategy across all tissues: 20% (95% CI: 15–26%) versus 23% (95% CI: 20–25%). However, we note that when we assign effects based on the relevant tissues and exclude the brain tissue we observe a FSR of 14% (95% CI: 9–20%), which is significantly different from the majority-vote estimate across all tissues ($p = 0.01$). This suggests that relevant-tissue assignment may reduce FSR in some settings, although we agree that it should not be presented as a complete or broadly practical solution on its own.

7. We often focus on tissues rather than cell types for TWAS because that is where the models have been developed. But heterogeneity among the cell types that make up a tissue is an important confounder, and as the field moves towards single-cell TWAS, it would be interesting to hear the authors' thoughts on how this confounding contributes to the false sign rate issue and the extent to which single-cell data can realistically mitigate it.

We agree with the author that cell heterogeneity within tissues can confound TWAS analysis. If specific populations of cells exert the causal effect of the variant, then it stands to reason that we need to train cell-type-specific expression models for TWAS. Our analysis in Figure 4 suggests that, indeed, if the correct context is used for TWAS, we should expect to observe the correct direction-of-effect.

Crucially, however, this relies on already knowing which cell type is important for a trait. If the cell type of interest is unknown, or in the interest of identifying novel cell type associations with traits, investigators often test against all cell-type-specific expression models (Zhou *et al.* 2025; Lin and Su 2026; Mai, Qian, Gao, *et al.* 2024). In this case, we

expect the false sign rate problem to occur similarly to how it occurs across tissues. eQTL in tight linkage, which are casual in different cell types, would still be expected to create misleading associations in this type of approach.

Thus, cell-type-specific expression models are crucial for modeling the causal signal in the correct context, especially if the effect of the variant is exerted via a specific subpopulation of cells. However, if the causal cell type is unknown, we expect that it will continue to be challenging to assign the correct direction-of-effect, even with the increased granularity of single cell expression data. We have revised the manuscript to clarify this in the discussion (page 14 line 40 and page 15 lines 1-3):

> *"The same issue may arise for cell-type-specific TWAS using single cell data – these models may improve direction-of-effect estimation when they capture the causal cell type, but testing many non-causal cell types can still produce discordant associations through linked eQTLs."*
